# Suppressing Polyamide Crystallization Unlocks Ultrapermeable and Highly Selective Nanofiltration Membranes

**DOI:** 10.1038/s41467-026-76628-8

**Published:** 2026-09-03

**Authors:** Hao Zhang, Jishan Wu, Xin Cao, Jingjing Guo, Menachem Elimelech, Yunkun Wang

**Affiliations:** 1 State Key Laboratory of Advanced Environmental Technology, School of Environment, University of Science and Technology of China, Hefei, 230026 China; 2 Department of Civil and Environmental Engineering, Rice University, Houston, TX 77005 USA; 3 Rice Center for Membrane Excellence (RiCeME), Rice University, Houston, TX 77005 USA; 4 Department of Chemical and Biomolecular Engineering, Rice University, Houston, TX 77005 USA

**Keywords:** Polymers, Pollution remediation, Polymer synthesis

## Abstract

Nanofiltration (NF) membranes are essential for water purification. However, their performance is still largely optimized through empirical methods, as polyamide crystallinity remain difficult to control in scalable interfacial polymerization processes. In this study, we introduce an ultra-low-temperature interfacial polymerization strategy at −20 °C that enables direct polyamide chain assembly, suppresses crystallization, and forms a smooth, defect-free, fully amorphous selective layer. Comprehensive characterization confirms the formation of a homogeneous amorphous network, enabling a fourfold increase in water permeability and a threefold improvement in Cl^-^/SO_4_^2-^ selectivity compared with room-temperature controls, surpassing high-performance NF membranes. The smooth surface further improves the antifouling performance of the NF membrane. Life cycle assessment based on experiments with real industrial wastewater, demonstrate that using the fabricated membrane can reduce treatment costs by 79.1%. This work establishes temperature-controlled chain assembly as a scalable lever for NF design and provides a practical pathway toward next-generation membranes for water treatment.

## Abstract (web summary)

Nanofiltration membranes are promising for industrial wastewater treatment, but it is challenging to optimize performance. Here the authors design an ultra-low-temperature interfacial polymerization suppressing crystallization optimizing membrane properties.

## Introduction

Nanofiltration (NF) membranes are widely applied in water purification and industrial wastewater treatment due to their outstanding separation performance and energy efficiency^1,2^. Over the past decades, extensive efforts have been devoted to regulating the structure of NF membranes through manipulation of the interfacial polymerization process to achieve optimal membrane performance^3,4^. The ideal NF membrane design should aim to improve water permeability, selectivity, and long-term antifouling performance, which are highly advantageous in separation processes^5,6^. Current research has made important advances in the morphology and composition of membranes, focusing on macroscopic and mesoscopic structural features such as surface morphology, thickness, and pore-size distribution, and has established structure-performance relationships^7,8^. However, the intrinsic polymer structure of the polyamide layer, such as chain conformation and crystallinity^9,10^, which is fundamental to its separation behavior, has received limited attention, leaving a critical gap in the rational design of next-generation high-performance NF membranes.

From a polymer-physics perspective, the performance of NF membranes is fundamentally governed by the intrinsic structure of the polyamide selective layer^11^. As a polymeric material, the polyamide layer exhibits an inherent tendency toward chain ordering and crystallization, which critically determines chain packing and nanoscale transport^12^. However, the role of these crystallographic features in determining membrane performance has not been systematically examined. Conventional interfacial polymerization proceeds rapidly and is highly sensitive to local interfacial conditions, often resulting in rough surface morphologies, heterogeneous chain organization, and localized microcrystalline domains^13,14^. These microcrystalline regions may act as structural defects, as their rigid and ordered nature introduces local transport barriers and tortuous pathways that hinder efficient water and solute transport^15^. Achieving precise control over interfacial polymerization to suppress microcrystallinity and construct a structurally uniform, defect-minimized polyamide layer, therefore, remains a central scientific and technological challenge in NF membrane design.

Despite extensive efforts to regulate interfacial polymerization, direct control over the crystalline structure of polyamide selective layers remains elusive. Reaction temperature, a primary parameter controlling polymer mobility, chain relaxation, and crystallization kinetics, has largely been constrained to a narrow window near room temperature in conventional interfacial polymerization^16,17^. Because the aqueous phase in interfacial polymerization is prone to freezing at temperatures below 0 °C, synthesis temperatures are inherently limited. Although several studies have shown that interfacial polymerization at a liquid–solid interface formed by an ultra-low temperature ice phase and a liquid oil phase can further enhance NF performance^18^, monomers can hardly undergo mass transfer within solid ice. Consequently, interfacial polymerization primarily occurs between the liquid phase formed by melting the ice phase and the organic phase. This physical constraint implies that the temperature of the interfacial polymerization process cannot be lowered below the freezing point of water. Hence, under existing control methods, the formation of microcrystalline domains is often unavoidable. Developing a synthesis strategy that enables precise regulation of polymer crystallization during interfacial polymerization, therefore, represents a critical step toward fundamentally advancing NF membrane design.

In this work, high-performance NF membranes were designed by adjusting the aqueous-phase composition to lower the liquid–liquid interfacial polymerization temperature to −20 °C. A combination of polymer characterization techniques, including small-angle X-ray scattering, differential scanning calorimetry, and X-ray diffraction, was employed to analyze the polyamide selective layer and quantify temperature-dependent changes in its crystalline structure. The water permeability, ion selectivity, and antifouling performance of the ultra-low-temperature NF membrane were evaluated through comparison with room-temperature NF membranes and representative state-of-the-art NF membranes reported in the literature. In addition, real high-salinity industrial wastewater was used to assess the membrane potential for practical wastewater treatment applications. This work provides a perspective on NF membrane design by shifting the focus toward the intrinsic physicochemical properties of polyamide materials and highlighting the fundamental role of polymer structure regulation in the fabrication of high-performance membranes.

## Results

### Synthesis of Amorphous NF membrane

**Fig. 1:**
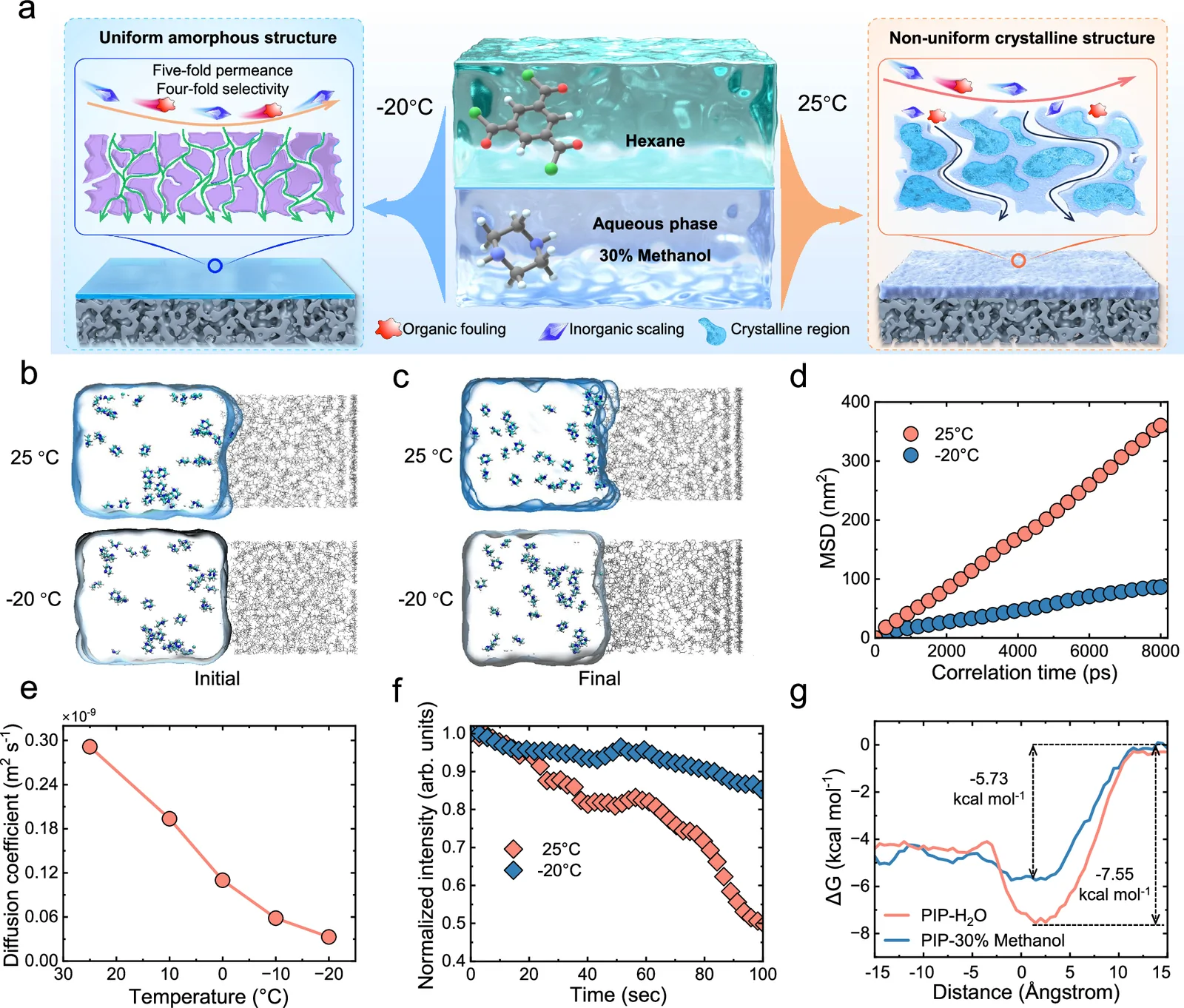
Synthesis of amorphous NF membranes. **a** Schematic diagram of the preparation and properties of amorphous NF membranes by ultra-low temperature liquid-liquid interfacial polymerization compared to membranes fabricated at room-temperature. Snapshots of the initial (**b**) and final state (**c**) models of the molecular dynamics simulations of the interfacial polymerization process. **d** Mean square displacement (MSD) curves of PIP monomers at different temperatures, highlighting the kinetics of interfacial polymerization. **e** The diffusion coefficient of PIP in 30% methanol aqueous solution at different temperatures as quantified by 2D DOSY NMR. **f** Changes of PIP concentration over time during interfacial polymerization at different temperatures. **g** The free energy (ΔG) of PIP versus the z-coordinate perpendicular to the interface in different models. The zero point on the horizontal (z-axis) corresponds to the interface between the aqueous phase and the organic phase. Negative z values represent the aqueous phase, while positive z values correspond to the organic phase.

The diffusion of piperazine (PIP) molecules from the aqueous phase to the organic phase has a decisive influence on the structure of NF membranes prepared by interfacial polymerization^19^. The system temperature directly affects the average kinetic energy of the monomers during interfacial polymerization, thereby affecting their diffusion in the liquid phase. During the synthesis of the NF membrane, a 30% methanol aqueous solution was used as the aqueous phase, allowing the reaction medium to remain liquid at an ultra-low temperature of −20 °C (Fig. 1a). This approach suppresses molecular diffusion while avoiding the uncontrolled transport limitations inherent to ice-assisted liquid–solid interfacial polymerization, thereby establishing a well-defined, kinetically constrained polymerization environment.

Molecular dynamics simulations were used to investigate the diffusion behavior of PIP molecules at both room temperature and ultra-low temperatures. As shown in Figs. 1b and c, hexane phase (right side) and water phase (left side) were constructed at different temperatures. Initially, PIP was uniformly dispersed in the aqueous phase. As the reaction proceeded, driven by diffusion, PIP tended to move towards the water-organic interface. However, low temperatures limit the diffusion of PIP. At the end of the simulation, the PIP molecules in the room-temperature system diffused to the interface but not in the ultra-low temperature system, visually demonstrating the difference in diffusion rates. The mean square displacement curves of PIP molecules in the different temperature systems show that the diffusion coefficient is reduced by 75% at the ultra-low temperature system (Fig. 1d).

These trends were independently validated by two-dimensional diffusion-ordered spectroscopy (DOSY) NMR measurements^20^. As shown in Fig. 1e, the diffusion coefficient of PIP molecules decreases with decreasing temperature. Notably, the diffusion rate at −20 °C is only 10% of that at room temperature, which is consistent with the results of molecular dynamics simulations.

Changes of PIP concentration were observed in situ by using a microplate reader to determine the effect of temperature (Fig. 1f). The concentration of PIP molecules in the room temperature system decreased rapidly as the reaction proceeded, while it decreased slowly in the ultra-low temperature system, indicating that ultra-low temperature successfully regulated the interfacial polymerization. The absorption spectra of PIP diffusing from the aqueous phase to the organic phase during interfacial polymerization at different temperatures were characterized using a UV-Vis spectrophotometer, as shown in Supplementary Fig. 1. As the temperature decreased, the amount of PIP diffused into the organic phase decreased, corroborating the results of the microplate reader assay.

In addition to temperature effects, methanol plays a critical role in modulating interfacial thermodynamics. Methanol molecules weaken the hydrogen bond interactions between water molecules, reducing the cohesive force of the system. Therefore, the surface tension of the methanol solution decreases with increasing methanol concentration. MD simulations demonstrated that the reduced interfacial tension could lower the energy barrier for the diffusion of PIP from the aqueous phase to the organic phase, resulting in smoother and more ordered diffusion of PIP at the water-organic interface (Fig. 1g). Experimental results further support this interpretation. Although 2D DOSY measurements showed a lower bulk diffusion coefficient of PIP in the 30% methanol–water solution than in pure water (Supplementary Fig. 2), the decrease in PIP concentration in the aqueous phase during the interfacial diffusion experiments was comparable for the two systems (Supplementary Fig. 3). This result suggests that the reduced bulk diffusivity of PIP in the methanol-containing solution does not lead to a corresponding reduction in its overall transport toward the reaction interface. Therefore, the influence of methanol on interfacial polymerization is unlikely to be governed solely by bulk diffusion. Instead, methanol may modify the interfacial environment and molecular interactions at the water/organic boundary, consistent with the MD simulations and supporting its role in promoting interfacial polymerization.

### Amorphous structure of the polyamide selective layer

**Fig. 2:**
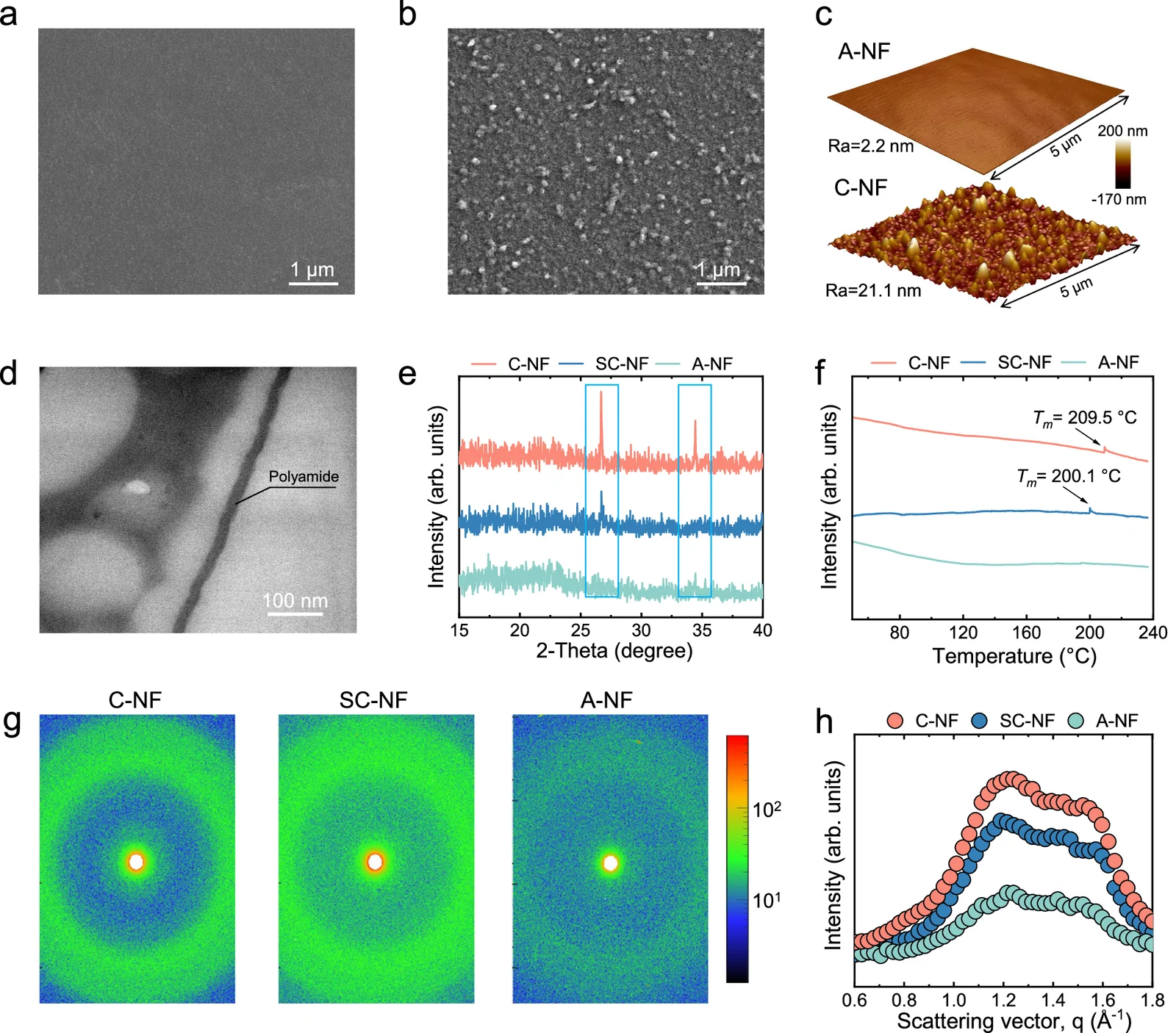
Structural characterization of amorphous NF membranes. Scanning electron microscopy (SEM) images of amorphous NF **(**A-NF) (**a**) and crystalline NF (C-NF) membranes (**b**). **c** Atomic force microscopy (AFM) characterization of active layer roughness of A-NF and C-NF. **d** Transmission electron microscope image of the A-NF section (**e**) X-ray diffraction (XRD) of NF membranes prepared at different temperatures. The samples consist of the pure polyamide selective layer without the substrate. Detailed sample preparation procedures are described in the Supplementary Information. **f** The melting temperature *T*_*m*_ of NF membranes prepared at different temperatures was determined by differential scanning calorimetry (DSC). A decrease in the melting temperature indicates a reduction in polymer crystallinity, while the absence of a melting peak suggests that the polymer exhibits an amorphous structure. Two-dimensional small-angle X-ray scattering (SAXS) patterns (**g**) and corresponding one-dimensional X-ray scattering data (**h**) of NF membranes prepared at different temperatures. The presence of crystalline domains gives rise to strong diffraction signals, whereas membranes with predominantly amorphous structures exhibit weak scattering intensity.

A series of characterization methods were used to evaluate the effect of ultra-low temperature interfacial polymerization on the structure and morphology of NF membranes. To systematically investigate the influence of interfacial polymerization temperature, three representative temperatures were selected, yielding membranes denoted as C-NF, SC-NF, and A-NF, prepared at 25 °C, 0 °C, and −20 °C, respectively. As shown in Fig. 2a–b and Supplementary Fig. 4 scanning electron microscopy (SEM) images show that as the temperature decreases, the polyamide layer transforms from a typical nodular structure to a completely smooth structure, indicating that the ultra-low temperature liquid-liquid interface makes the interfacial polymerization process orderly and controllable.

The roughness of the NF membrane was characterized by atomic force microscopy (AFM). As shown in Fig. 2c and Supplementary Fig. 5, the ultra-low temperature liquid-liquid interface polymerization has a marked effect, reducing the average surface roughness of the NF membrane from 21.1 nm to 2.2 nm. The height curve (diagonal) from the AFM test illustrates this more clearly (Supplementary Fig. 6). Compared to NF membranes prepared at room temperature, the surface at ultra-low temperatures shows almost no undulations. The smooth surface can reduce the adhesion of foulants and help improve the anti-fouling performance of the NF membrane^21^. Furthermore, the ultralow-temperature system successfully reduced the thickness of the polyamide layer from 65 nm to 32 nm (Fig. 2d and Supplementary Fig. 7). The reduced thickness of the selective layer shortens the transport pathway for water molecules, thereby facilitating water permeation. The above results show that adding methanol to enable a system temperature of −20 °C is conducive to the formation of a thinner and smoother polyamide layer, thereby significantly improving water permeability and anti-fouling performance.

In addition to conventional morphological characterization, crystallographic methods were used to evaluate the effect of the ultra-low-temperature polymerization system on the crystal structure of the polyamide NF membrane, including X-ray diffraction (XRD), differential scanning calorimetry (DSC), and two-dimensional small-angle X-ray scattering (SAXS) patterns. XRD data clearly show that the polyamide layer of the NF membrane prepared at room temperature has a typical crystalline structure with obvious diffraction peaks appearing near 26 ° and 35 ° (Fig. 2e), similar to the diffraction structure of other polyamide polymers^22^. As the interfacial polymerization temperature decreases, the crystalline structure gradually weakens and almost disappears completely, indicating that ultra-low temperature liquid-liquid interfacial polymerization transforms the crystalline structure of polyamide into amorphous.

The DSC test results offer more direct evidence to support this conclusion^23^. As shown in Fig. 2f, obvious melting endothermic peaks appear in the DSC curves of C-NF and SC-NF, and the corresponding melting temperatures (*T*_*m*_) are 209.5 and 200.1 °C, respectively. The lower *T*_*m*_ indicates the decrease in the crystallinity of polyamide. However, no melting endothermic peak and melting temperature appear in A-NF prepared by ultra-low temperature liquid-liquid interfacial polymerization, providing direct evidence for its amorphous structure^24^.

To further elucidate the effects of ultra-low-temperature liquid-liquid interfacial polymerization on the polyamide crystalline structure, C-NF, SC-NF, and A-NF were characterized using two-dimensional small-angle X-ray scattering (2D-SAXS) patterns, as shown in Fig. 2g. The 2D-SAXS clearly reveals differences in the scattering intensities of the three NF membranes. Specifically, while C-NF exhibits the strongest scattering, A-NF shows virtually no intensity, suggesting a near-total absence of crystalline domains and a predominantly amorphous structure. The integrated one-dimensional X-ray scattering profile (Fig. 2h) shows that C-NF exhibits two characteristic diffraction peaks centered at scattering vectors (q) of 1.2 and 1.5 Å, which were attributed to the presence of crystalline regions within the PA layer^14^. These peaks nearly disappear as the preparation temperature decreases, again demonstrating that ultra-low-temperature liquid-liquid interfacial polymerization eliminates polyamide crystalline, consistent with the XRD and DSC results.

X-ray photoelectron spectroscopy (XPS) was used to characterize the surface and internal chemical structures of A-NF and C-NF. As shown in Supplementary Fig. 8, the surface of A-NF has a higher oxygen content compared to that of C-NF, mainly because the surface of A-NF has more carboxyl groups (Supplementary Fig. 9). A higher oxygen-nitrogen ratio usually indicates a more porous structure in polyamides^25^. However, it should be noted that the surface O/N ratio may not reliably reflect the intrinsic crosslinking degree of the selective layer, since XPS primarily probes only the outermost surface region, where oxygen-rich terminal groups are more likely to accumulate. Internal XPS analysis shows that A-NF and C-NF have similar oxygen-nitrogen ratios (Supplementary Fig. 8), indicating that their bulk chemical composition and crosslinking density remain essentially unchanged. The increased carboxyl group content endows A-NF with a more negative surface potential (Supplementary Fig. 10), which is beneficial for the rejection of divalent anions by strengthening Donnan exclusion effects and thereby contributes to enhanced membrane selectivity. Meanwhile, the water contact angle shows only a negligible change (Supplementary Fig. 11).

The density characterization of the polyamide layer provides a perspective on its structure. The density of the polyamide selective layer was determined using a liquid suspension method, as shown in Supplementary Fig. 12. The precise density was obtained directly by suspending the polyamide layer in a liquid of the same density (details in Supplementary Method). The densities of C-NF and A-NF were 1.249 and 1.245 g/cm³, respectively, which are close to those determined by other methods or simulations^26,27^. The slight decrease in density can be attributed to the reduction in the crystalline region, as the density of the crystalline region is generally higher than that of the amorphous region.

### Nanofiltration performance of Amorphous NF

The molecular weight cutoff (MWCO) profile of is a key parameter for evaluating NF membrane separation performance. The molecular weight cutoff and pore size distribution of the NF membranes were evaluated via neutral solute retention experiments using polyethylene glycol of different molecular weights, as shown in Supplementary Fig. 13. Both A-NF and C-NF exhibited an identical MWCO of 334 Da. However, analysis of pore size distribution curves reveals that A-NF possesses a larger average pore size (0.55 nm) than C-NF (0.44 nm). Crucially, A-NF displays a significantly narrower pore size distribution, which enhances separation selectivity.

**Fig. 3:**
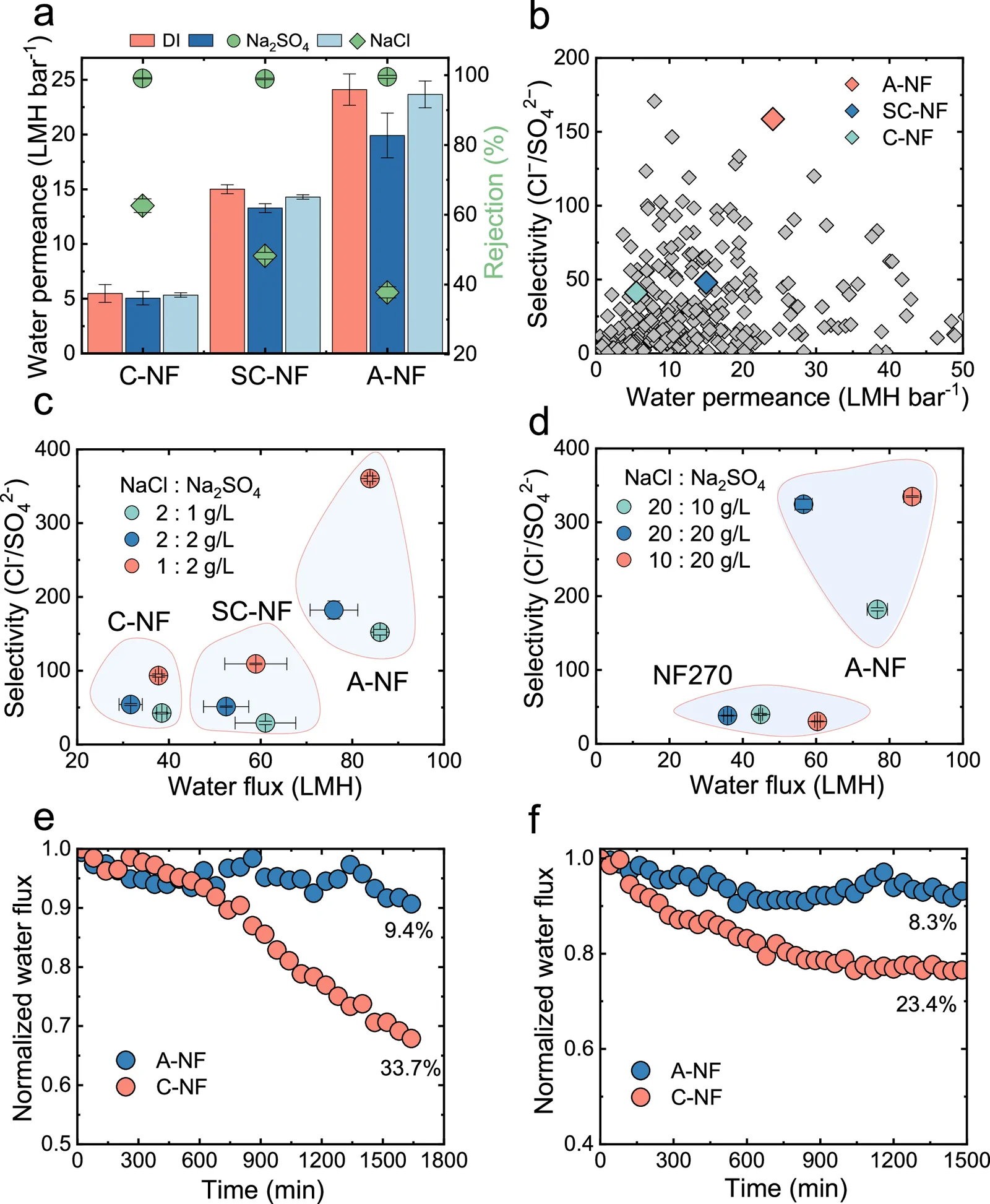
NF performance of A-NF. **a** Effects of interfacial polymerization temperature on permeance and salt rejection performance of NF membranes. **b** Summary of water permeance as well as NaCl/Na_2_SO_4_ selectivity of NF membranes reported in the literature and available commercially. The gray diamond symbols represent literature data points corresponding to the membranes listed in Supplementary Table 1. **c** Salt separation selectivity and water flux for low-concentration mixed solutions. **d** Salt separation selectivity and water permeance for high-concentration mixed solutions. **e** Anti-scaling performance of A-NF and C-NF with feed solution containing 15 mM CaCl_2_, 10 mM Na_2_SO_4_, and 10 mM NaCl; the gypsum (CaSO_4_·2H_2_O) saturation index (SI) of this solution is 0.54. **f** Anti-organic fouling performance of A-NF and C-NF. Humic acid concentration: 50 mg L^-1^. CaCl_2_ concentration: 50 mg L^-1^. Applied pressure: 6.9 bar in experiments (**a**–**c**, e and **f**), and 20 bar in (**d**); cross-flow velocity: 23.1 cm s^−1^, temperature: 25.0 ± 0.5 °C, salt concentration: 10 mM unless otherwise noted, feed volume: 5 L. Error bars in (**a**, **c**, **d**) represent the standard deviation (s.d., *n*  =  3, n derived from different experimental units) and data are presented as mean values ±  s.d. LMH denotes L m^−2^ h^−1^.

Water permeance and salt rejection of the NF membranes are shown in Fig. 3a and Supplementary Fig. 14. The pure water permeance of A-NF increased by 440% compared to C-NF, reaching 24.1 LMH/bar. The elimination of crystalline domains in the A-NF membrane reduced the polyamide's resistance to water transport, thereby improving water permeability. In contrast, the high-density crystalline regions in C-NF restrict water transport to fewer and more tortuous pathways, significantly impeding flow. Furthermore, the reduction in polyamide-layer thickness by approximately half (from 65 nm to 32 nm) also contributed to the improved permeance, as water permeability is inversely proportional to the selective-layer thickness. To further decouple the thickness effect, the water permeance was normalized by the corresponding selective-layer thickness (Supplementary Fig. 15). Notably, the thickness-normalized water permeance of A-NF remained approximately twofold higher than that of C-NF, indicating that the enhanced water transport cannot be solely attributed to the reduced selective-layer thickness.

The applicability of the ultralow-temperature interfacial polymerization strategy was further validated using PEI- and MPD-based systems (detailed fabrication procedures for these additional membrane systems are provided in the Supplementary Methods). As shown in Supplementary Figs. 16, 17, both systems exhibited substantially improved water permeability under ultra-low-temperature conditions. In particular, the PEI-based membrane achieved a 50.1% increase in water permeance together with improved salt rejection (89.3% to 94.6%), while the MPD-based membrane showed a 51% increase in water flux. These results confirm the broad applicability of this strategy across different interfacial polymerization systems, although the most pronounced permeability–selectivity enhancement was observed for the PIP-based nanofiltration membrane.

Due to the increased surface charge, a stronger Donnan potential is formed, enabling high retention of divalent anions^28^. The more negative surface potential enhances the high retention of divalent anions and the permeation of divalent cations. A-NF exhibits a 99.5% rejection for Na_2_SO_4_, but only 25.4% for CaCl_2_ (Fig. 3a and Supplementary Fig. 14). Due to its smaller hydration radius, Cl^-^ rejection is more sensitive to changes in pore size. Increasing the pore size of A-NF leads to a decrease in the rejection of NaCl from 67.6% to 37.3%.

The increased SO_4_^2-^ rejection and decreased Cl^-^ rejection enhance the Cl^-^/ SO_4_^2-^ selectivity of A-NF compared to C-NF. As shown in Fig. 3b and Supplementary Table 1, the selectivity of A-NF is superior to that of state-of-the-art NF membranes reported in the literature and to that of various commercial NF benchmarks. Taken together, these results suggest that A-NF achieves a competitive balance between water permeance and ion selectivity, comparing favorably with advanced NF membranes reported in the literature. As discussed below, the water permeance and selectivity of A-NF suggest that this membrane may also perform well in salt fractionation from reverse osmosis concentrate for zero liquid discharge (ZLD) industrial wastewater treatment^29^.

The stability of NF membranes under continuous operation is an important parameter for evaluating their practical viability. As shown in Supplementary Fig. 18, the Na_2_SO_4_ rejection of A-NF remained stable during 120 hours of continuous operation. In addition, the performance of A-NF was evaluated over a wide range of operating pressures (Supplementary Fig. 19). As the operating pressure increased from 5 to 30 bar, the water flux increased nearly linearly, while the Na_2_SO_4_ rejection remained at around 99%. These results demonstrate that A-NF can maintain stable separation performance under the elevated pressures commonly employed in practical nanofiltration applications.

Nanofiltration membranes are central to salt fractionation in industrial wastewater treatment^30,31^, where selective separation of monovalent and divalent ions is critical for downstream process efficiency. The presence of both monovalent and divalent anions can affect the separation performance of NF membranes. As shown in Supplementary Fig. 20, due to co-ion competition in a mixed-salt solution, more Cl^-^ ions are transported into the permeate to maintain charge balance, leading to a decrease in Cl^-^ rejection. Conversely, for the highly negatively charged NF membrane, the high flux of Cl^-^ further enhances the rejection of SO_4_^2-^ by Donnan exclusion^32,33^.

The application potential of A-NF in zero liquid discharge of high-salinity wastewater was verified through low-concentration and high-concentration mixed salt separation experiments. As shown in Fig. 3c, compared with C-NF, A-NF exhibited higher Cl^-^/ SO_4_^2-^ selectivity and water permeance in the low-concentration mixed salt separation experiment. At a NaCl to Na_2_SO_4_ ratio of 2:1, the Cl^-^/ SO_4_^2-^ selectivity of A-NF reached as high as 400, far exceeding the single-salt rejection selectivity.

The separation performance of high-concentration mixed salt solutions was tested at an operating pressure of 20 bar. The commercial NF membrane, NF270, commonly used in actual industrial wastewater treatment, was used as a comparison, as shown in Fig. 3d and Supplementary Figs. 21 and 22. A-NF demonstrated clear advantages in both Cl^-^/ SO_4_^2-^ selectivity and water permeance. Furthermore, the selectivity of A-NF increased with increasing SO_4_^2-^ concentration in the mixed salt solution, consistent with observations in the literature ^32^.

NF membrane fouling leads to a sharp decline in water flux and necessitates higher operating pressures, which drives up energy consumption and costs while compromising both separation efficiency and system longevity. The smooth surface of A-NF results in lower foulant adhesion. Furthermore, the high permeability of A-NF to Ca^2+^ alleviates inorganic scaling as well as organic fouling^34,35^.

In scaling experiments conducted with both calcium and sulfate ions present, C-NF exhibited a 33.7% decline in water flux after 1600 minutes of continuous operation (Fig. 3e), whereas the flux of A-NF decreased by only 9.4%, demonstrating its superior resistance to inorganic scaling. As shown in Supplementary Fig. 23, SEM images of the two NF membranes after operation clearly illustrate the difference in surface scaling. Specifically, gypsum deposition on the A-NF surface is significantly sparser and less structured than that on C-NF. This enhanced scaling resistance is mainly attributed to the higher calcium ion permeation of A-NF, which mitigates local ion accumulation and suppresses surface supersaturation, as well as to its smoother surface morphology, which reduces potential nucleation sites for gypsum crystallization^35^.

The adhesion of organic foulants to the surfaces of A-NF and C-NF was tested in situ using a quartz crystal microbalance^36^. Humic acid was selected as a typical organic foulant. As shown in Supplementary Fig. 24, humic acid adsorption on A-NF was consistently lower than that on C-NF, indicating greater intrinsic resistance to organic fouling. This antifouling advantage is expected to be further enhanced under practical cross-flow filtration conditions, where hydrodynamic shear can effectively suppress foulant accumulation. In addition, the enhanced negative surface charge of A-NF strengthens electrostatic repulsion toward negatively charged humic acid molecules, further contributing to its reduced foulant adsorption.

Dynamic cross-flow filtration experiments with humic acid solutions further confirmed the superior organic fouling resistance of A-NF. During 1500 min of continuous operation, water permeance of A-NF decreased by only 7% compared with a 20% decline for C-NF (Fig. 3f). SEM images showed a nearly pristine surface for the A-NF after anti-fouling performance tests, whereas aggregates could be identified on the C-NF (Supplementary Fig. 25). This enhanced antifouling performance is attributed to the smooth surface of the A-NF membrane, which minimizes foulant anchoring sites, together with its higher passage of calcium ions, thereby mitigating calcium-bridging interactions that are known to dramatically enhance organic fouling^35^.

### Industrial wastewater treatment

**Fig. 4:**
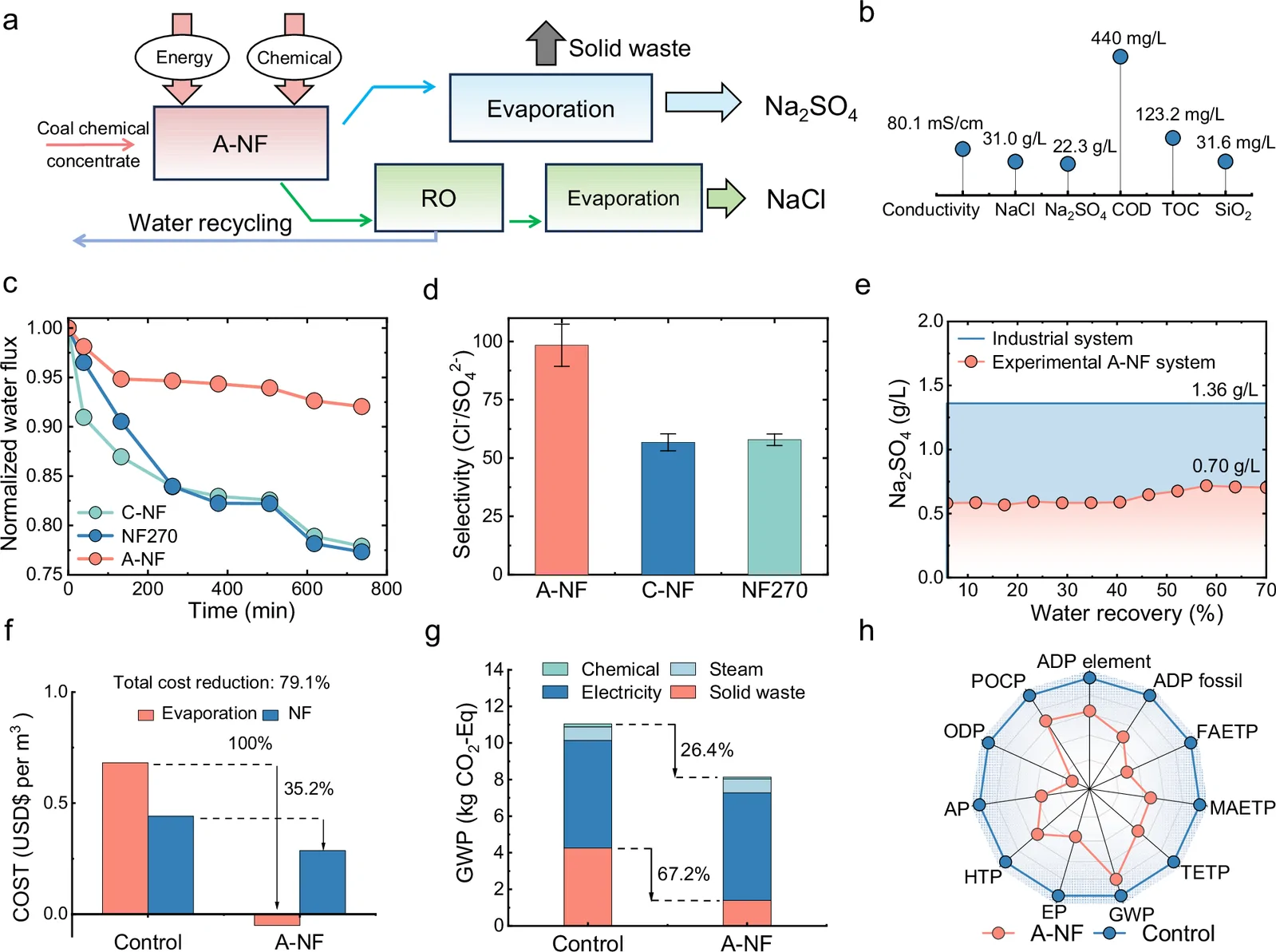
High-salinity coal chemical wastewater treatment. **a** Zero liquid discharge process for high-salinity coal chemical wastewater. **b** Components of high-salinity coal chemical wastewater. **c** Comprehensive anti-fouling performance evaluation of A-NF, C-NF, and NF270 during long-term operation. **d** Salt separation selectivity of high-salinity coal chemical wastewater. **e** Variation of Na_2_SO_4_ concentration in the permeate with water recovery rate. **f** Cost analysis of a salt crystallization system based on A-NF. **g** Global Warming Potential of a salt crystallization system based on A-NF. **h** radar chart comparing the environmental impact indicators of the A-NF-based treatment system with those of the original system for industrial wastewater. Applied pressure: 20 bar, cross-flow velocity: 23.1 cm s^−1^, temperature: 25.0 ± 0.5 °C, feed volume: 5 L. Error bars in (**c**, **d**) represent the standard deviation and data are presented as mean values ±  s.d.

NF membranes play a crucial role in the ZLD of high-salinity wastewaters. As shown in Fig. 4a, in a typical ZLD process, the NF system serves as the primary unit capable of fractionating monovalent and divalent salts. This, in turn, directly determines the purity of the subsequent crystallized salts and the proportion of salt solid waste. Lowering divalent anion concentrations in the permeate favors the selective crystallization of NaCl during downstream evaporation–crystallization, increasing salt purity while reducing the formation of low-value mixed salts. To evaluate the performance of A-NF under real-world conditions, high-salinity wastewater from the coal chemical industry was used as the feed. The wastewater composition, detailed in Fig. 4b, reveals a total salt content exceeding 53 g/L along with high levels of carcinogenic organic pollutants and inorganic silica. It should be noted that the coal chemical wastewater concentrate was subjected to a Ca^2+^ removal pretreatment before membrane filtration, reducing the residual Ca^2+^ concentration to approximately 8 mg/L (Supplementary Fig. 26).

The comparative performance of A-NF, C-NF, and NF270 was evaluated in a continuous crossflow filtration system. As shown in Fig. 4c, C-NF and NF270 showed rapid flux decline during operation, while A-NF demonstrated higher fouling resistance. A-NF achieved a high SO_4_^2-^ rejection of 99.16% for SO_4_^2-^ coupled with a low Cl^-^ rejection of 17.5% (Supplementary Figs. 27–29). Consequently, the observed Cl^-^/ SO_4_^2-^ selectivity reached 100, substantially outperforming NF270 and C-NF, for which the selectivity remained below 60 (Fig. 4d). The slightly reduced selectivity compared with laboratory-scale measurements can be attributed to higher concentration polarization and the competitive adsorption of feed constituents under continuous crossflow operation, which partially screens membrane surface charge and weakens Donnan exclusion during Cl^-^/ SO_4_^2-^ separation^37^. Meanwhile, A-NF achieved a TOC removal efficiency of 94.1% (Supplementary Fig. 30), slightly higher than that of NF270 (92.5%), indicating that its superior ion selectivity was maintained without compromising organic matter removal performance.

In the NF membrane salt separation process, the water recovery rate is a critical factor influencing the separation efficiency of the system components. The salt separation performance of an A-NF-based nanofiltration system was evaluated at a water recovery of 70%. As shown in Fig. 4e, at a water recovery rate of 70%, the Na_2_SO_4_ concentration in the permeate of the A-NF system was only 0.7 g/L, which is greatly lower than the 1.36 g/L obtained from a conventional industrial treatment process operated under comparable wastewater conditions. Reducing the Na_2_SO_4_ concentration in the permeate facilitates the crystallization of high-purity NaCl while minimizing the generation of mixed-salt solid waste.

A life cycle assessment and global-warming-potential analysis were conducted to evaluate the water treatment costs and environmental impact of implementing A-NF within the salt separation and crystallization system(Supplementary Tables 2–5). As shown in Fig. 4f, the improved anti-fouling characteristics and high permeate flux reduced the operational expenditures of the salt separation stage by 35.4%. Furthermore, the reduced sodium sulfate concentration in the feed water during the subsequent crystallization stage decreased mixed salt solid waste while increasing the output of high-purity NaCl product. This increased product output fully offset the costs of that stage, ultimately leading to a 79.1% reduction in the overall cost of the salt separation and crystallization process. Our analysis underscores the critical role of salt separation precision; specifically, minimizing Na_2_SO_4_ crossover is directly associated with lower overall processing costs.

Life cycle and carbon emission analyses highlight the importance of precise ion fractionation. Mixed-salt solid waste is typically treated by incineration, which accounts for the largest share of the ZLD process's carbon footprint. By reducing mixed-salt formation, the A-NF-based process achieves a 67.2% reduction in carbon emissions from solid waste (Fig. 4g), ultimately leading to a 26.4% reduction in emissions from the ZLD process. Beyond global warming potential, midpoint environmental impact indicators consistently show lower environmental burdens across all categories compared with the conventional ZLD process (Fig. 4h), demonstrating that precise salt separation at the membrane level translates directly into enhanced environmental sustainability at the system scale. However, despite its promising performance in wastewater treatment applications, further systematic studies are still needed to evaluate its scalability and behavior under full-scale operational conditions. The additional energy consumption associated with the low-temperature process was also evaluated (Supplementary Methods). The results indicate that these additional inputs are expected to be relatively small, suggesting that the proposed strategy remains promising from both practical and sustainability perspectives.

## Discussion

This work presents a perspective by examining how the underlying architecture of the polyamide selective layer influences NF membrane performance through the lens of polymer physics and chemistry. The amorphous NF membrane exhibited more than a 400% increase in water permeability and more than a 300% increase in Cl⁻/SO₄²⁻ selectivity, surpassing the performance of current state-of-the-art nanofiltration membranes. The use of low-freezing-point solvents to depress the aqueous-phase freezing point expands the temperature-tuning window for interfacial polymerization, opening opportunities for further optimization of membrane architecture and the development of membranes with even higher separation performance.

Viewing NF membranes through this polymer-centric lens reveals that deliberate manipulation of these intrinsic structural parameters is not merely an auxiliary optimization, but a necessary and powerful design strategy. Emphasizing polymer crystallography and molecular-level structural regulation repositions NF membrane development within the broader framework of rational polymer design, in which controlling chain organization is essential to unlocking combinations of permeability, selectivity, and anti-fouling performance. This work highlights that advancing high-performance NF membranes requires preparation strategies grounded in the deliberate regulation of polymer structure at the molecular level.

## Methods

### Materials and chemicals

Polysulfone (PSF, MWCO: 50 K) ultrafiltration membrane was purchased from RisingSun Membrane Technology Co., Ltd (Beijing). Commercial NF membranes (NF270) were purchased from Dow Inc. Trimesoyl chloride (TMC), piperazine (PIP), m-phenylenediamine (MPD), polyethyleneimine (PEI), and n-hexane were purchased from Shanghai Aladdin Biochemical Technology Co., Ltd. Sodium sulfate (Na_2_SO_4_), calcium chloride (CaCl_2_), glycerin, polyethylene glycol (PEG, 200, 300, 400, 600 and 800 Da), hydrochloric acid (HCl), sodium hydroxide (NaOH), sulfuric acid (H_2_SO_4_), and sodium chloride (NaCl) were purchased from Sinopharm Chemical Reagent Co., Ltd. High-performance liquid chromatography (HPLC) grade methanol was purchased from Sigma-Aldrich (Shanghai) Trading Co., Ltd. Humic acid (HA) and sodium alginate (SA) were purchased from Sigma Aldrich (St. Louis, Missouri, USA). Unless otherwise specified, all chemicals used in this study were of analytical grade and used as received without further purification. All solutions used in this work were prepared with 18-MΩ ultrapure deionized water (PURELAB flex Ultrapure Water System).

### Fabrication of Amorphous NF membrane

NF membranes were fabricated via interfacial polymerization of PIP and TMC. Cleaned PSF membranes were fixed with the active layer facing upward on a 10 × 13 cm glass plate, and surface water was removed with a rubber roller. The membranes were immersed in 1 wt% PIP and 30% methanol aqueous solution at -20°C for 2 min, drained, and then contacted with 0.15 wt% TMC in *n*-hexane for 30 s to form the polyamide selective layer. Infrared thermal imaging measurements confirmed that the membrane substrate retained an ultralow temperature immediately prior to immersion in the n-hexane phase (Supplementary Fig. 31). Specifically, the NF membrane obtained by interfacial polymerization of aqueous solution at room temperature (25 °C) is referred to as the crystalline NF membrane, while the NF membrane prepared by 30% methanol aqueous solution at -20°C is referred to as the amorphous NF membrane.

### Membrane characterization

The surface morphology and elemental mapping of the membranes were characterized using a Schottky field emission scanning electron microscope (SEM, GeminiSEM 500, Zeiss, Germany). The thickness and roughness of the active layer were measured by atomic force microscopy (AFM, Dimension Icon, Bruker, USA). Attenuated total reflection-Fourier transform infrared (ATR-FTIR, Nicolet 8700, Thermo Scientific, USA) and X-ray photoelectron (XPS, Thermo-VG Scientific Inc, Thermo Scientific, USA) spectra were employed for functional group and elemental analysis. A drop shape analyzer (OCA 25, KRUSS, Germany) was used to measure the water contact angle of the membranes. The surface potential of the membranes was determined using a streaming potential analyzer (SurPASS 3, Anton Paar, Austria). The concentrations of polyethylene glycol (PEG) were quantified using a total organic carbon Analyzer (TOC, multiN/C 3100, Analytik Jena, Germany). A high-resolution X-ray diffractometer for thin films (XRD, XPert3 MRD, Malvern Panalytical, UK) was used to determine the crystalline structure of the membrane. Small-angle X-ray scattering (SAXS, Xeuss 3.0 UHR, XENOCS SAS, France) was used to obtain two-dimensional SAXS patterns. The melting temperature (Tm) of the polyamide was measured by a differential scanning calorimeter (DSC, DSC 204F1, NETZSCH-Gerätebau GmbH, Germany) at a heating/cooling rate of 20 °C min^−1^. The concentration of anions was determined by ion chromatography (IC, DIONEX INUVION, Thermo Scientific, USA).

### Ion separation performance

The performance of membranes was evaluated using a custom-made cross-flow membrane filtration system consisting of four parallel membrane cells, each with an effective filtration area of 3.10 cm^2^. The cross-flow velocity was set at 23.1 cm s^-1^ and the temperature was 25.0 °C, except for the pressure stability experiments. The membrane was compacted with deionized water at 10 bar until a stable flux was observed before taking any measurements. The water permeance (*P*) of the membrane (L m^−2^ h^−1^ bar^−1^) was calculated by

1 $$P=\Delta {{{\rm{V}}}}/({{{\rm{A}}}}\Delta {{{\rm{t}}}}\Delta {{{\rm{P}}}})$$

where Δ*V* is the volume of permeate water (L) collected during a time interval of Δ*t* (h), *A* is the effective filtration area (m^2^), and Δ*P* is the applied pressure (bar), respectively. The rejection efficiency (*R*) was calculated according to

2 $$R=(1-{C}_{p}/{C}_{0})\times 100\%$$

where *C*_*o*_ and *C*_*p*_ are the salt concentrations in the feed liquid and the filtrate, respectively.

## Supplementary information

Supplementary Information

Peer Review File

## Source data

Source Data

## Data Availability

All data presented in this work are fully available from the corresponding authors. Source data are provided with this paper.
