# Supplementary material for: Suppressing Polyamide Crystallization Unlocks Ultrapermeable and Highly Selective Nanofiltration Membranes
Source: Nat Commun. 2026 Sep 3;17:10481. doi: 10.1038/s41467-026-76628-8 (PMC13635223; doi:10.1038/s41467-026-76628-8)
Supplement: Supplementary file 1 [file 41467_2026_76628_MOESM1_ESM.pdf]

Supplementary Information

Suppressing Polyamide Crystallization Unlocks Ultrapermeable and Highly Selective Nanofiltration Membranes

Hao Zhang [1, 2, 3], Jishan Wu [2, 3], Xin Cao [1], Jingjing Guo [1], Menachem Elimelech [2, 3, 4, *], Yunkun Wang [1, *]

[1] State Key Laboratory of Advanced Environmental Technology, School of Environment, University of Science and Technology of China, Hefei 230026, China

[2] Department of Civil and Environmental Engineering, Rice University, Houston, TX 77005, USA

[3] Rice Center for Membrane Excellence (RiCeME), Rice University, Houston, TX 77005, USA

[4] Department of Chemical and Biomolecular Engineering, Rice University, Houston, TX 77005, USA

* Correspondence and requests for materials should be addressed to M.E. (email: menachem.elimelech@rice.edu) or to Y.W. (email: ykwang@ustc.edu.cn)

Table of contents

Supplementary Methods 4
Sample preparation method 4
PEI-based NF membranes preparation method 4
MPD-based RO membranes preparation method 4
Pore size analysis method 4
Polyamide density measurement method 5
Computational Methods 5
Simplified Thermodynamic Estimation of Cooling Energy Consumption 7
Life Cycle Assessment 7
Supplementary Figures 8
Supplementary Fig. 1: Temperature-dependent diffusion of PIP. 8
Supplementary Fig. 2: Diffusion coefficient of PIP. 9
Supplementary Fig. 3: Temporal evolution of PIP concentration. 10
Supplementary Fig. 4: Surface morphology of SC-NF. 11
Supplementary Fig. 5: Surface roughness of SC-NF. 12
Supplementary Fig. 6: Surface roughness profiles. 13
Supplementary Fig. 7: Thickness of the membrane. 14
Supplementary Fig. 8: XPS characterization. 15
Supplementary Fig. 9: High-resolution XPS spectra. 16
Supplementary Fig. 10: Surface charge characterization. 17
Supplementary Fig. 11: Hydrophilicity characterization. 18
Supplementary Fig. 12: Density characterization. 19
Supplementary Fig. 13: Pore size analysis. 20
Supplementary Fig. 14: Nanofiltration performance. 21
Supplementary Fig. 15: Thickness-normalized water permeance. 22
Supplementary Fig. 16: Separation performance of MPD-based membranes. 23
Supplementary Fig. 17: Separation performance of PEI-based membranes. 24
Supplementary Fig. 18: Stability of nanofiltration membranes. 25
Supplementary Fig. 19: Pressure stability of membrane. 26
Supplementary Fig. 20: Schematic diagram of co-ion competition effect. 27
Supplementary Fig. 21: Rejection of SO_4^{2-}. 28
Supplementary Fig. 22: Rejection of Cl^-. 29
Supplementary Fig. 23: Surface morphology after anti-scaling test. 30

Supplementary Fig. 24: QCM analysis. 31
Supplementary Fig. 25: Surface morphology after anti-fouling test. 32
Supplementary Fig. 26: Ca^{2+} concentration. 33
Supplementary Fig. 27: Nanofiltration performance under actual wastewater treatment. ... 34
Supplementary Fig. 28: SO_4^{2-} passage under actual wastewater treatment. 35
Supplementary Fig. 29: Rejection of Cl^- under actual wastewater treatment. 36
Supplementary Fig. 30: TOC removal performance. 37
Supplementary Fig. 31: Temperature changes of the membrane substrate. 38
Supplementary Tables 39
Supplementary Table 1. Comparison of selectivity and permeance data from the literature with this work. 39
Supplementary Table 2. The I-O list of NF membrane unit. 49
Supplementary Table 3. The I-O list of NaCl crystallization unit. 50
Supplementary Table 4. The I-O list of A-NF membrane unit. 51
Supplementary Table 5. The I-O list of NaCl crystallization unit (A-NF). 52

Supplementary Methods

Sample preparation method

Nanofiltration membranes of 1 m^2 were prepared in batches at different temperatures. The polysulfone substrate was dissolved using N,N-dimethylformamide to separate the polyamide layer. The completely separated polyamide layer was transferred to 50 ml centrifuge tubes and freeze-dried to obtain polyamide foam. The polyamide foam was pressed into tablets to obtain samples for crystallographic characterization.

PEI-based NF membranes preparation method

PEI-based NF membranes were fabricated via interfacial polymerization of PEI and TMC. Cleaned PSF membranes were fixed with the active layer facing upward on a 10 × 13 cm glass plate, and surface water was removed with a rubber roller. The membranes were immersed in 1 wt% PEI and 30% methanol aqueous solution at -20°C for 2 min, drained, and then contacted with 0.15 wt% TMC in n-hexane for 30 s to form the polyamide selective layer.

MPD-based RO membranes preparation method

MPD-based RO membranes were fabricated via interfacial polymerization of MPD and TMC. Cleaned PSF membranes were fixed with the active layer facing upward on a 10 × 13 cm glass plate, and surface water was removed with a rubber roller. The membranes were immersed in 3.4 wt% MPD, 2% TEA and 30% methanol aqueous solution at -20°C for 2 min, drained, and then contacted with 0.15 wt% TMC in n-hexane for 120 s to form the polyamide selective layer.

Pore size analysis method

Rejections of neutral organic species with different molecular weights, including glycerol (92 Da), PEG (200, 300, 400, 600 and 800 Da), were assessed at a feed concentration of 200 mg L^{-1} to determine molecular weight cut off (MWCO) and pore size distribution of the membranes. The concentration of solute was detected with a total organic carbon

analyzer (TOC, multiN/C 3100, Analytik Jena, Germany). MWCO is defined as the molecular weight of the solute corresponding to a retention rate of 90%. Membrane pore size distribution was considered to follow the log-normal probability density function (PDF). The mean pore size (μ_p) of the membrane is equal to the Stokes radius (r_p) of the solute at a retention rate of 50%. The geometric standard deviation (σ_p) of the PDF curve is the ratio between the Stokes radius of a solute with 84.13% retention and the Stokes radius of a solute with 50% retention. The membrane pore size distribution is calculated by the equation (1) based on μ_p and σ_p.

$$\frac{dR(r_p)}{dr_p} = \frac{1}{r_p \ln \sigma_p \sqrt{2\pi}} exp\left[-\frac{(\ln r_p - \ln \mu_p)^2}{2(\ln \sigma_p)^2}\right] \quad (1)$$

Since the integral areas of PDFs of different membrane samples are not equal and <1, they are normalized.

Polyamide density measurement method

The nanofiltration membrane was immersed in N,N dimethylformamide to dissolve the polysulfone substrate and obtain the separated polyamide layer. The polyamide layer was then transferred to ultrapure water for complete washing. The polyamide layer was then transferred to a saturated aqueous solution of $NaNO_3$. At this point, the solution density was greater than the polyamide density, causing it to float on the surface. Ultrapure water was added to the solution to dilute it and reduce its density, and the floating state of the polyamide was observed. As the solution density decreased, the polyamide gradually transitioned from a floating state to a suspended state, at which point the density of the polyamide became equal to that of the solution. The density of the polyamide could then be obtained by measuring the density of the solution using an analytical balance.

Computational Methods

Molecular dynamics (MD) simulations were performed using the LAMMPS package to investigate the diffusion of PIP from a 30% methanol aqueous solution into the n-hexane phase. A biphasic simulation box containing PIP, a 30% (v/v) MeOH/H_2O mixture, and n-hexane was constructed, with the two liquid phases arranged along the z-direction to form a stable liquid–liquid interface. Periodic boundary conditions were applied in all three

dimensions. Water molecules were modeled using the TIP3P force field, while PIP, methanol, and n-hexane were described by the OPLS-AA force field. Atomic charges and bonded/non-bonded parameters followed standard OPLS-AA assignments, and Lennard-Jones cross-interactions were generated using the Lorentz–Berthelot combination rules. Electrostatic interactions were treated with the particle–particle particle-mesh (PPPM) Ewald method, and both real-space electrostatic and Lennard-Jones interactions used a cutoff of 10–12 Å.

Simulations were performed at two temperatures, −20 °C and 25 °C. For each temperature, the system was first energy-minimized and equilibrated under the NPT ensemble (T = −20/25 °C, P = 1 atm) to achieve stable density and interfacial structure. Temperature and pressure were controlled using the Nosé–Hoover thermostat and barostat. Production simulations were then conducted in the NVT ensemble with a time step of 1.0 fs, and total simulation times of several tens of nanoseconds ensured sufficient sampling of the PIP interfacial migration process. The velocity-Verlet algorithm was used for time integration, and all covalent bonds involving hydrogens were constrained using the SHAKE algorithm.

To quantify the free-energy barrier associated with PIP transfer across the interface, umbrella sampling was carried out along the z-coordinate of the PIP molecular center of mass, which serves as the reaction coordinate perpendicular to the interface. A series of overlapping sampling windows were constructed, each restrained by a harmonic bias potential. For both −20 °C and 25 °C, biased MD trajectories were collected for all windows. The potential of mean force (PMF) profile was reconstructed using the weighted histogram analysis method (WHAM), and the free-energy barrier for diffusion was obtained from the PMF difference between the interfacial maximum and the minima corresponding to the methanol/water and n-hexane basins. Together with diffusion coefficients derived from the mean-square displacement (MSD), the computed PMF profiles allow a comprehensive evaluation of the temperature dependence and interfacial effects on PIP transport across the liquid–liquid boundary.

Simplified Thermodynamic Estimation of Cooling Energy Consumption

Rough calculation process:

Volume of precursor solution: V=100 L

Mass of solution: m ~ 100 kg(assuming the density of the solution is approximately equal to that of water)

Specific heat capacity: Cp=4.18 kJ kg^{-1} K^{-1} (the specific heat capacity of a 30% methanol–water solution is slightly lower than that of pure water, and therefore this calculation provides a conservative estimation of the cooling load)

Temperature decrease: ΔT=25-(-20)=45 °C

The theoretical cooling load is: Q= m Cp ΔT= 18810 kJ= 5.23 kWh

Considering a practical refrigeration coefficient of performance (COP) of 1.5–2.0:

E=Q/COP= 2.62 kWh−3.49 kWh

Assuming a typical electricity emission factor: 0.45–0.55 kg CO_2-eq kWh^{-1}

The carbon footprint is: 1.2−1.9 kg CO_2-eq per batch

Life Cycle Assessment

The functional unit of this LCA study was defined as the treatment of 1 m³ of high-salinity wastewater to achieve ZLD. The system boundary encompasses the process of concentrated high-salinity wastewater entering the salt separation and crystallization unit, thereby achieving ZLD. Within this boundary, the A-NF membrane replaced the conventional nanofiltration units. Under this boundary, the environmental impacts of A-NF were assessed using GaBi software (version 9.2.1, Education) and the CML-IA baseline method (January 2016). The life cycle inventory was established using operational data obtained from actual coal chemical plants achieving ZLD, as well as pilot-scale experimental data for the A-NF treating high-salinity wastewater. A life cycle cost analysis covering salt separation and crystallization stages was also conducted, with the prices of chemicals and materials obtained from China National Pharmaceutical Group Chemical Reagent Co., Ltd. For chemicals not included in the GaBi database, upstream tracing and substitution were performed to construct their representative life cycle inventories.

Supplementary Figures

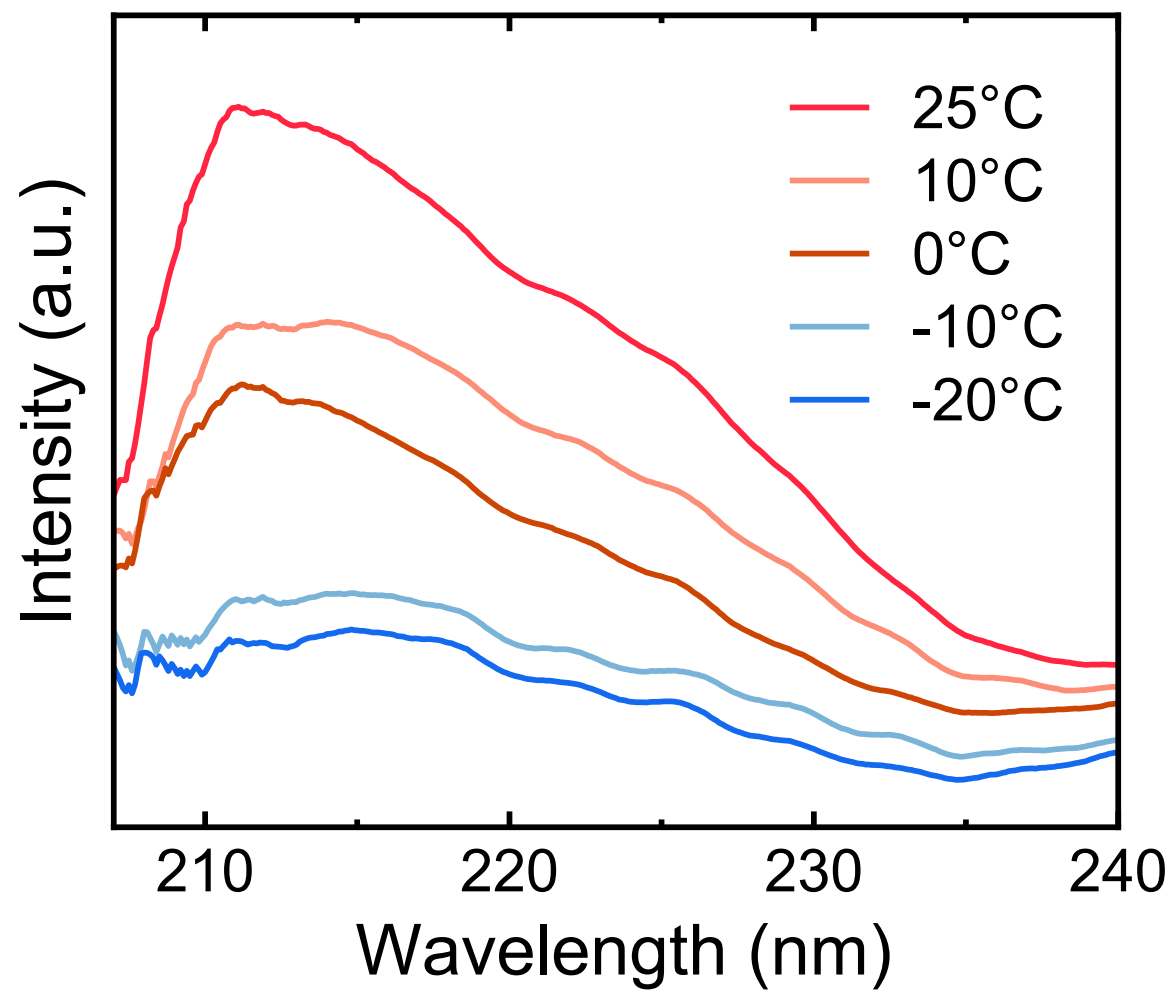

Supplementary Fig. 1: Temperature-dependent diffusion of PIP. UV absorption spectra of PIP diffused into the organic phase per unit time at different temperatures

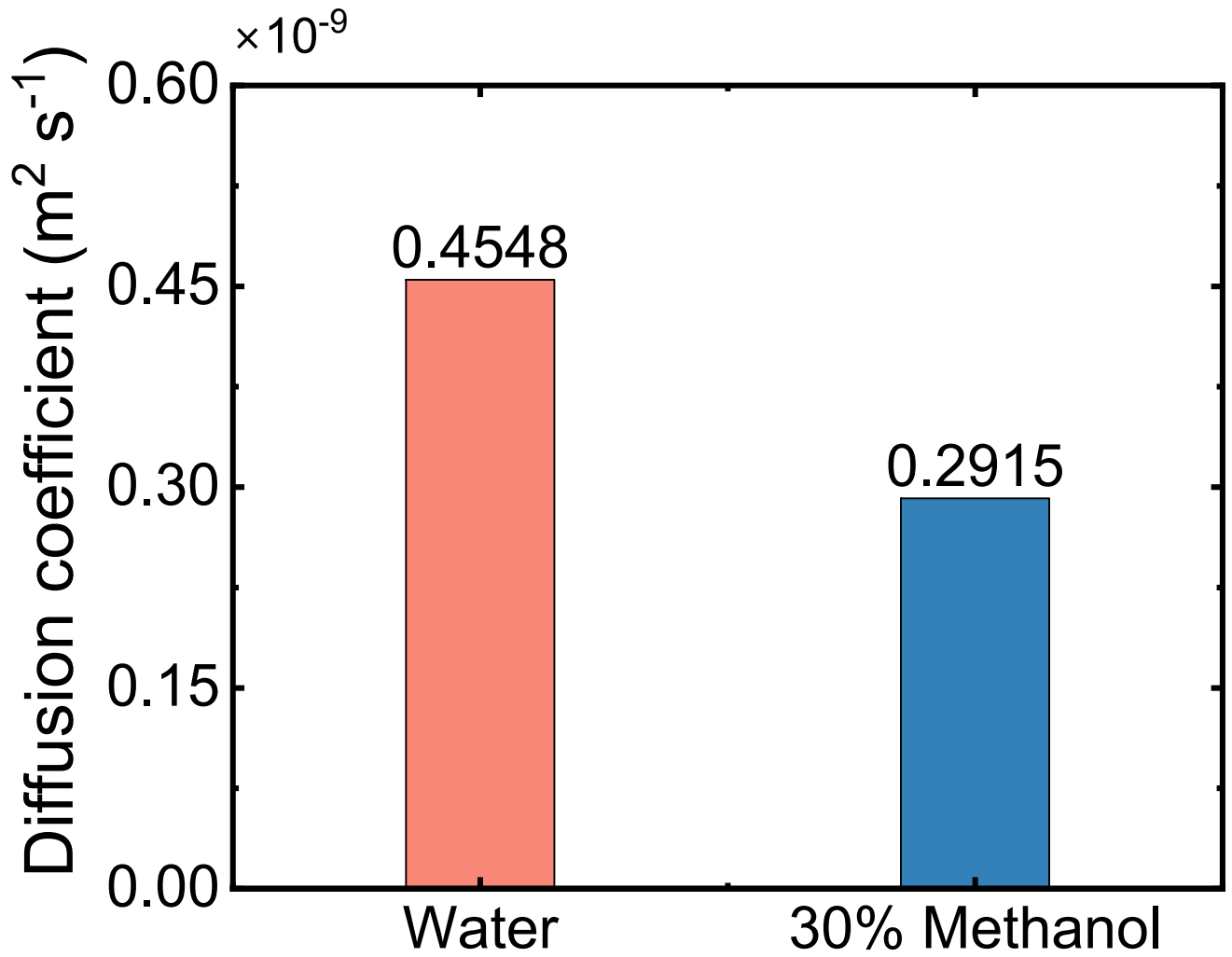

Supplementary Fig. 2: Diffusion coefficient of PIP. The diffusion coefficient of PIP in 30% methanol aqueous solution and water as quantified by 2D DOSY NMR

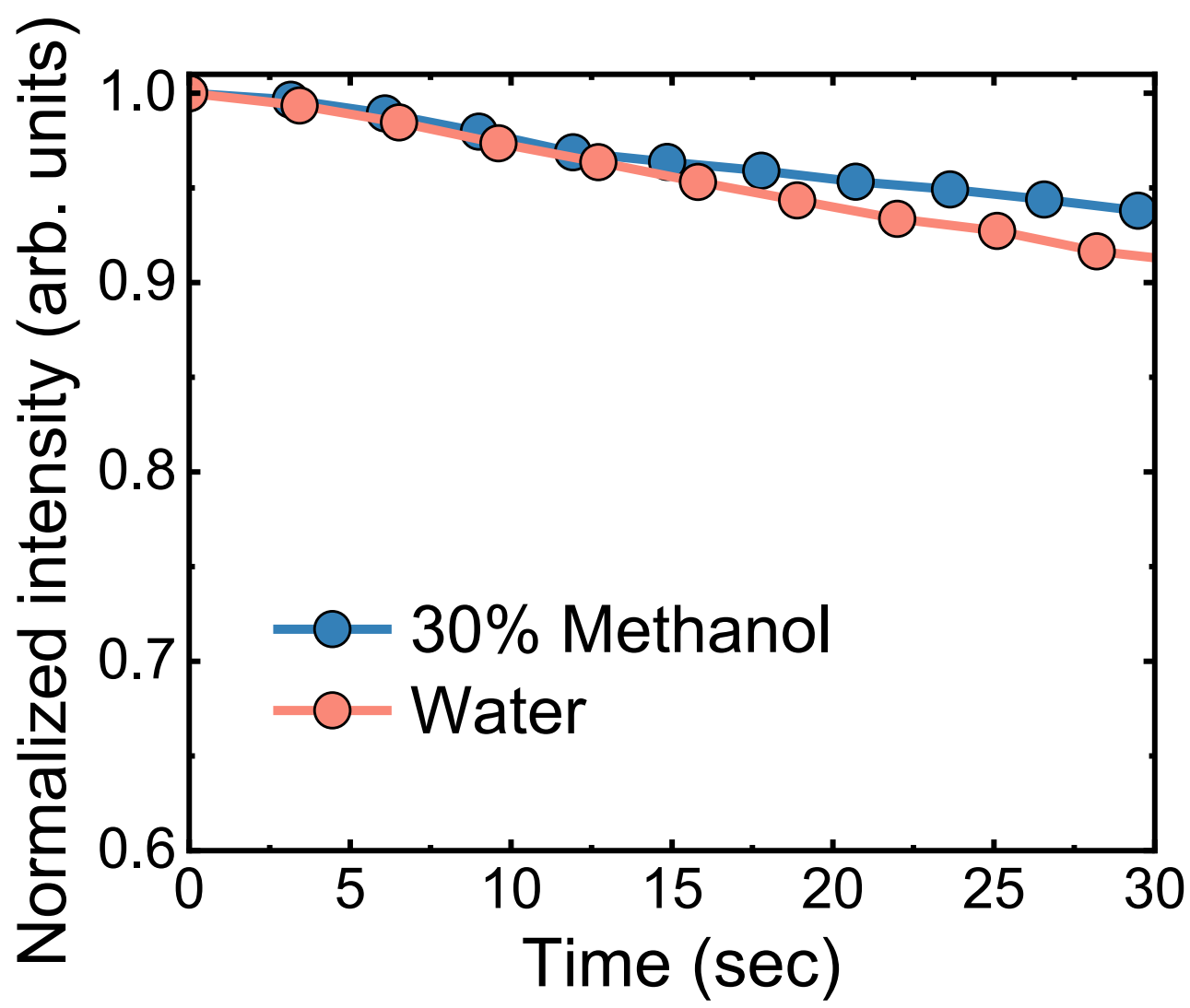

Supplementary Fig. 3: Temporal evolution of PIP concentration. Changes of PIP concentration over time during interfacial polymerization in 30% methanol aqueous solution and water.

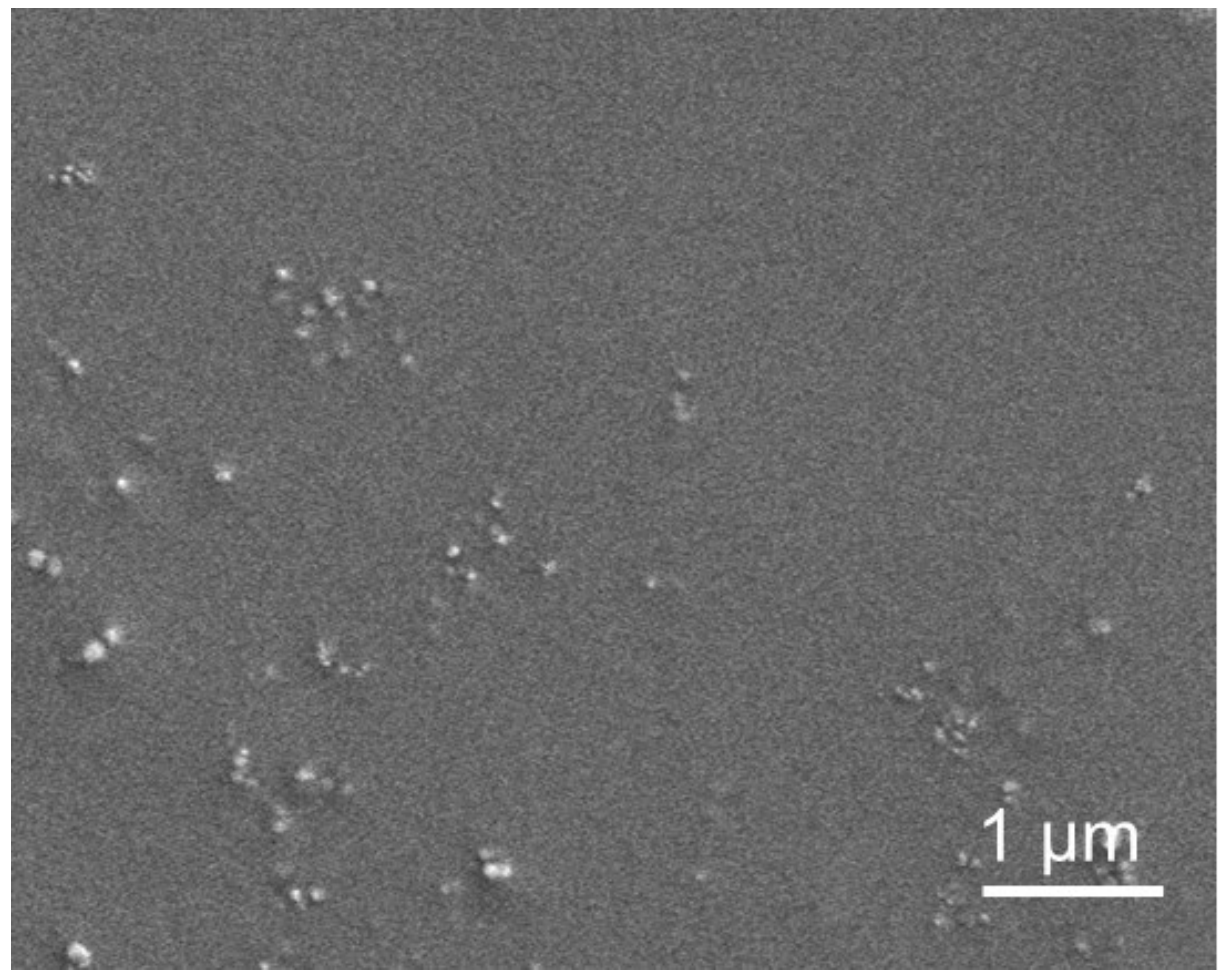

Supplementary Fig. 4: Surface morphology of SC-NF. Scanning electron microscopy (SEM) images of SC-NF

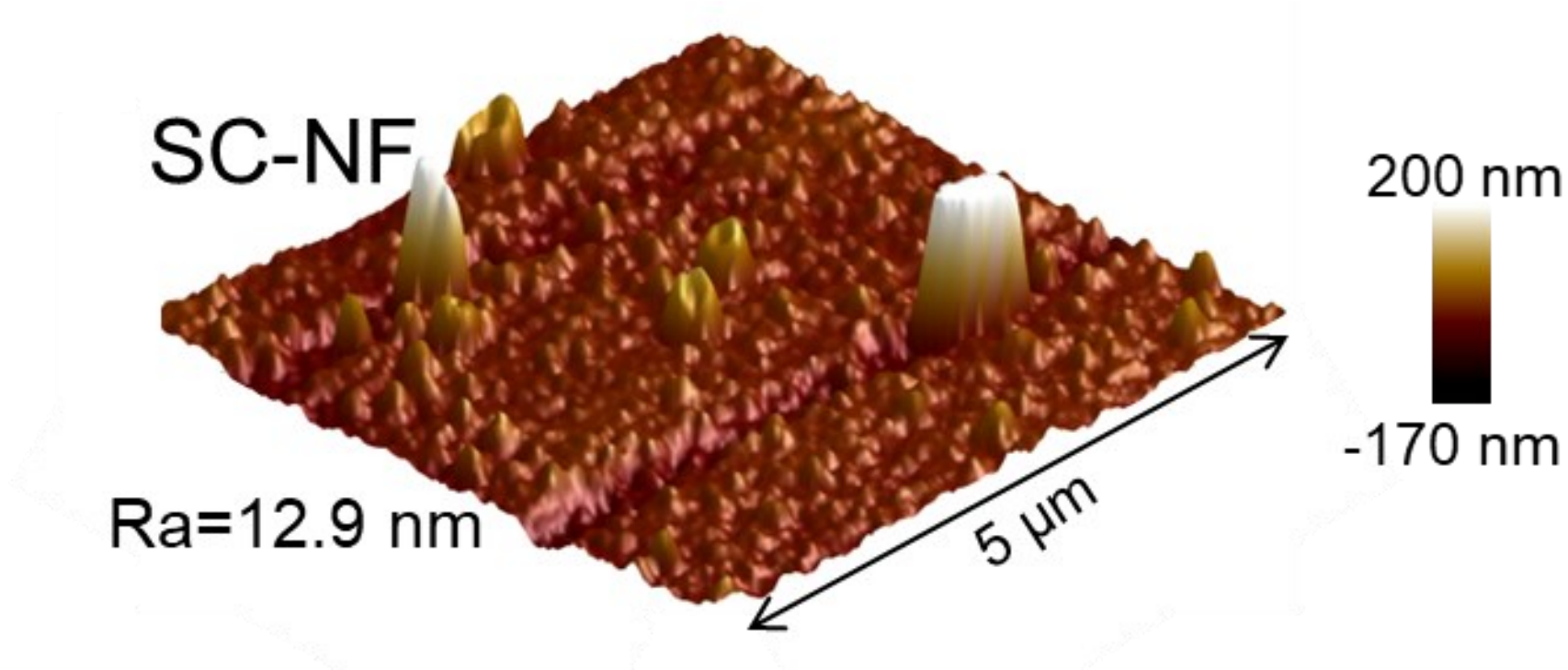

Supplementary Fig. 5: Surface roughness of SC-NF. Atomic force microscopy (AFM) characterization of active layer roughness of SC-NF (SC-NF is a semi-crystalline nanofiltration membrane fabricated at 0 °C).

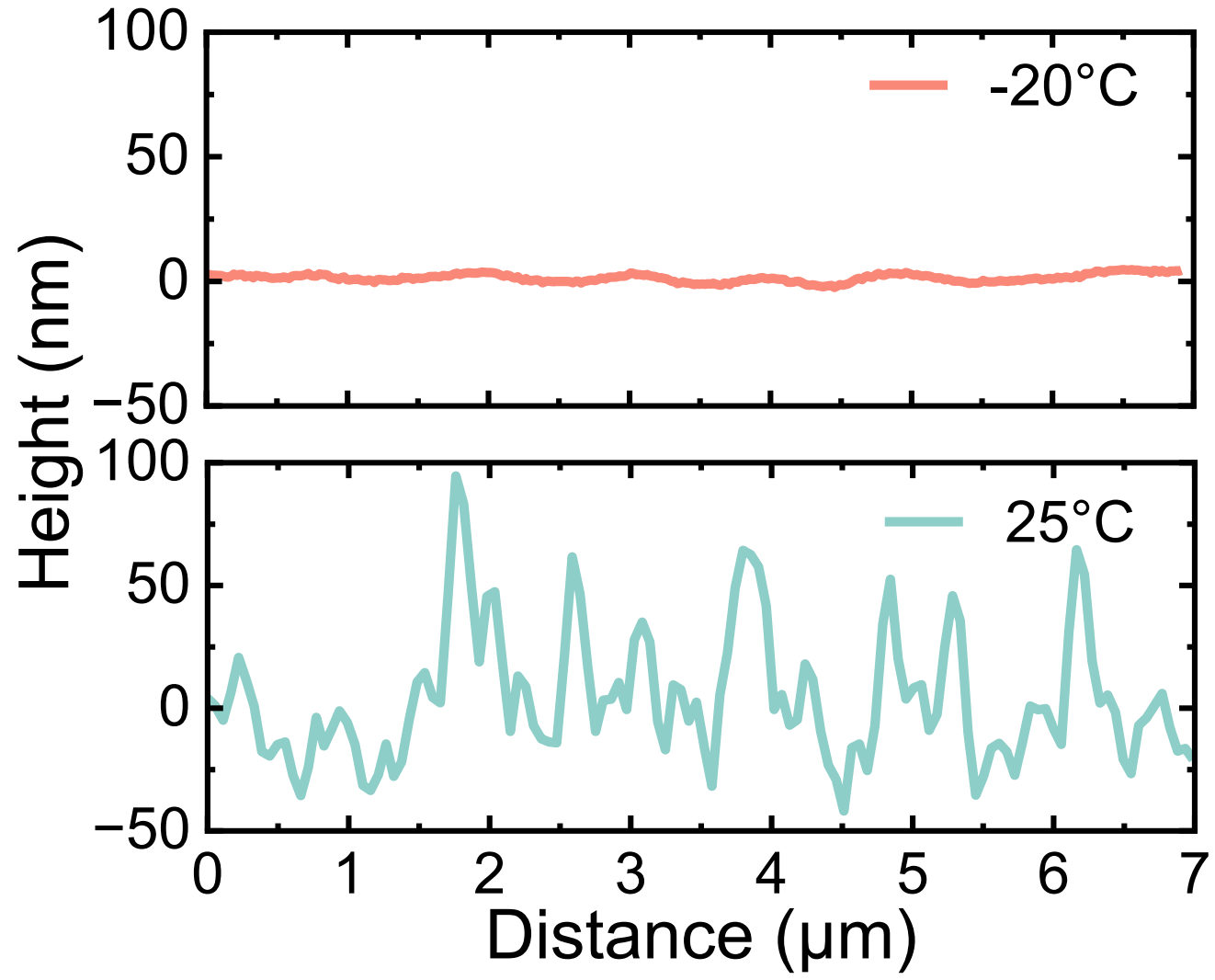

Supplementary Fig. 6: Surface roughness profiles. Surface roughness curves of nanofiltration membranes prepared at different temperatures.

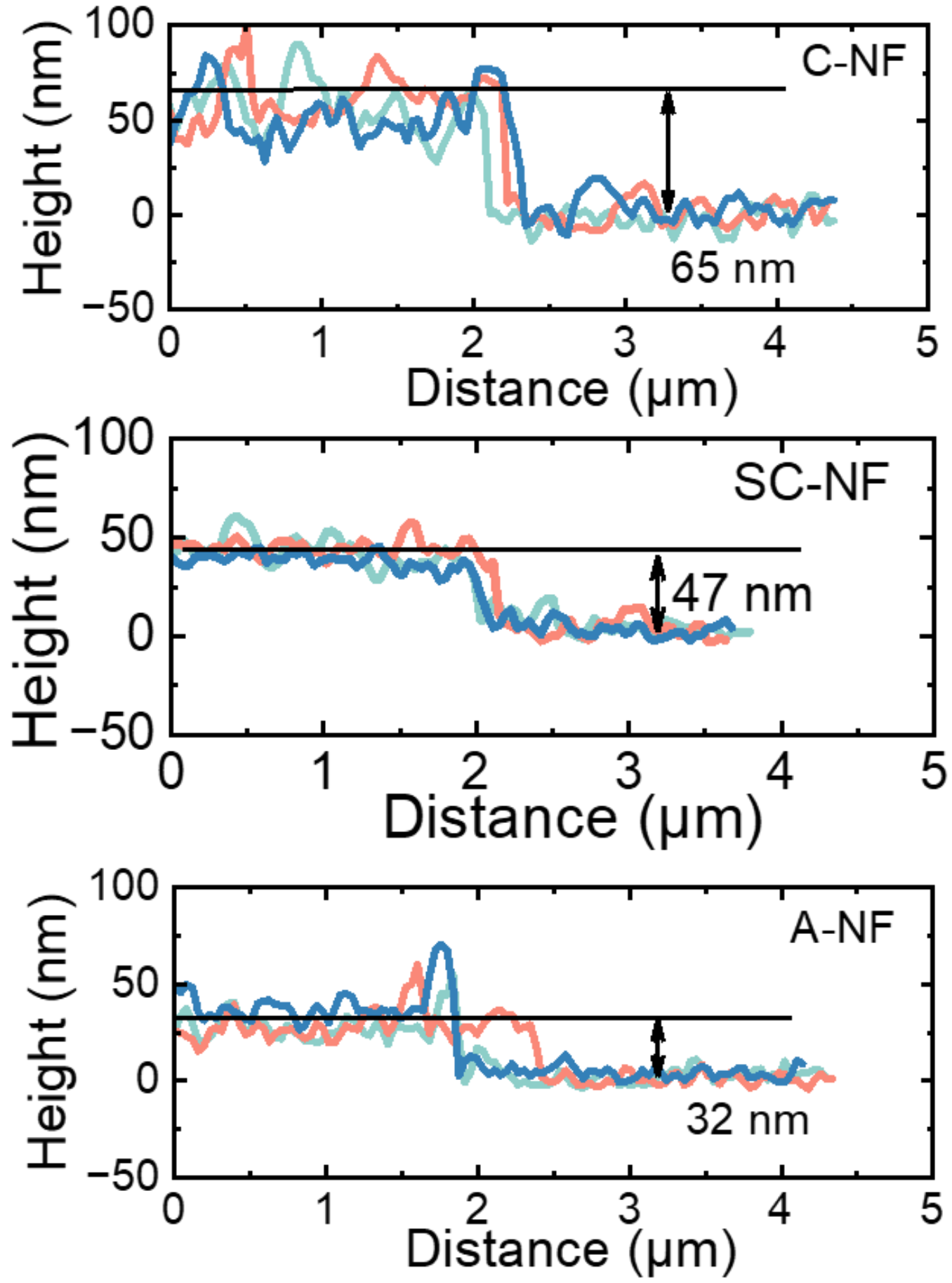

Supplementary Fig. 7: Thickness of the membrane. Atomic force microscopy (AFM) characterization of active layer thickness of the membrane.

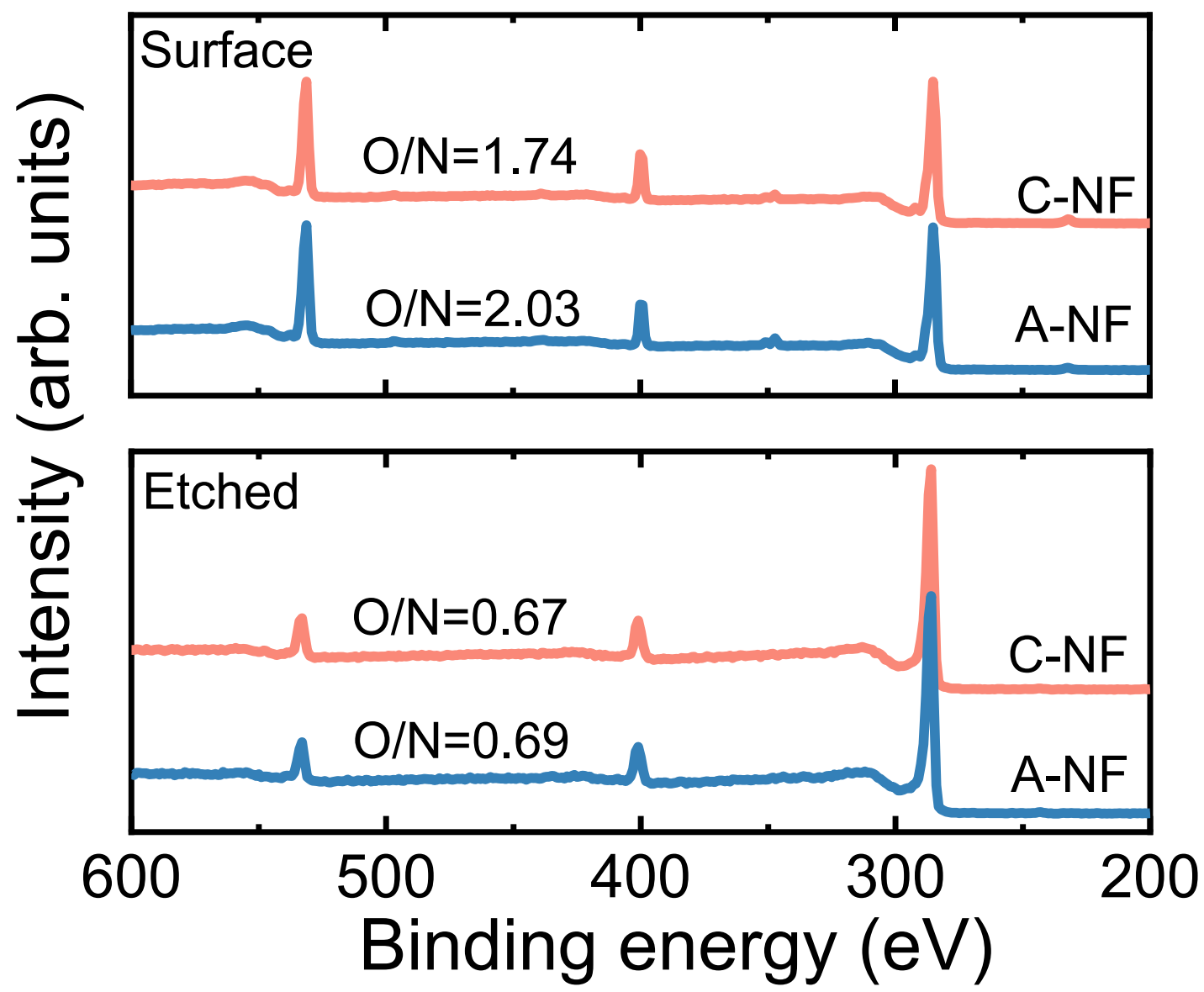

Supplementary Fig. 8: XPS characterization. The XPS spectra of A-NF and C-NF surfaces before and after etching.

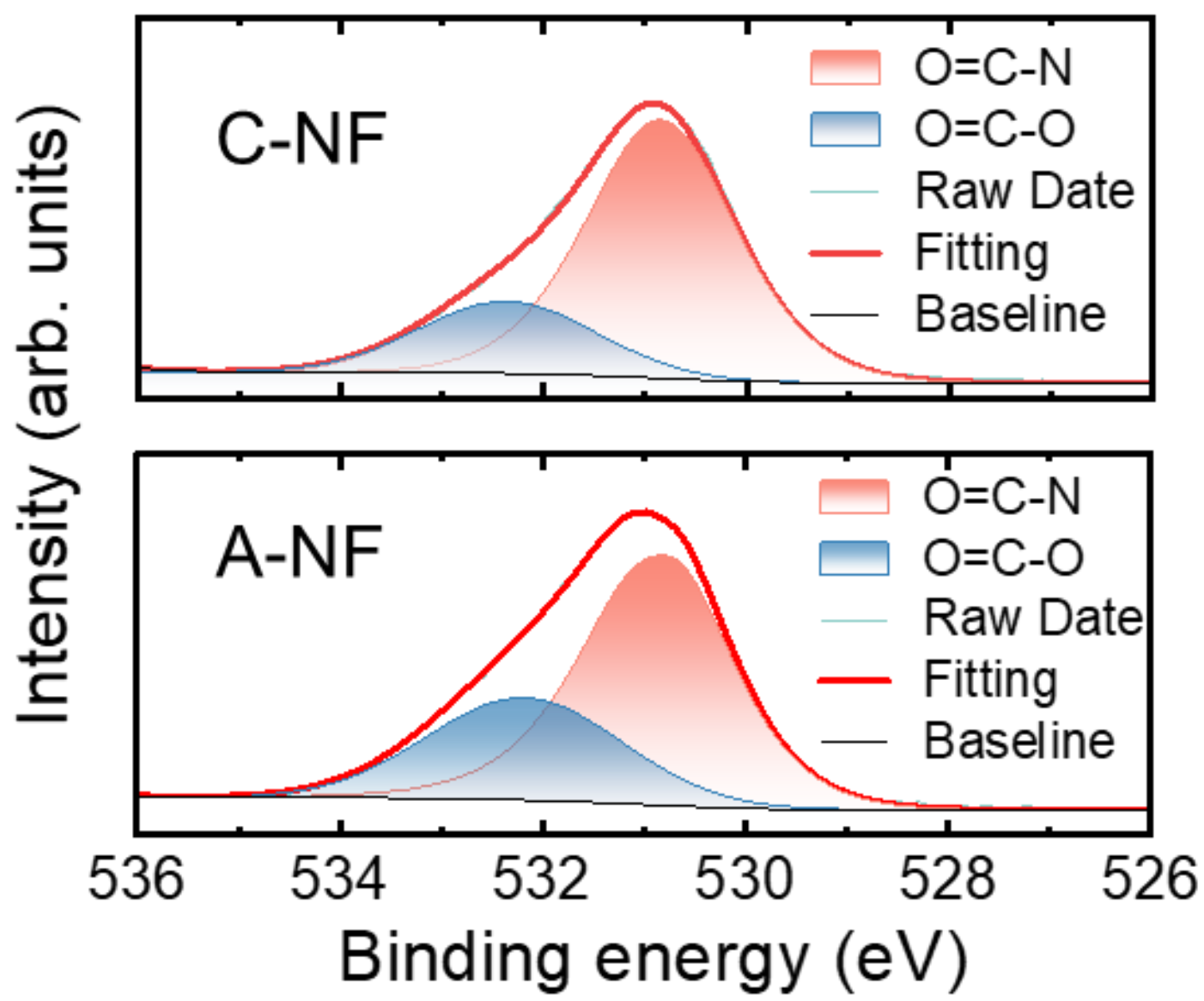

Supplementary Fig. 9: High-resolution XPS spectra. High-resolution O*1s* spectra on the surfaces of A-NF and C-NF.

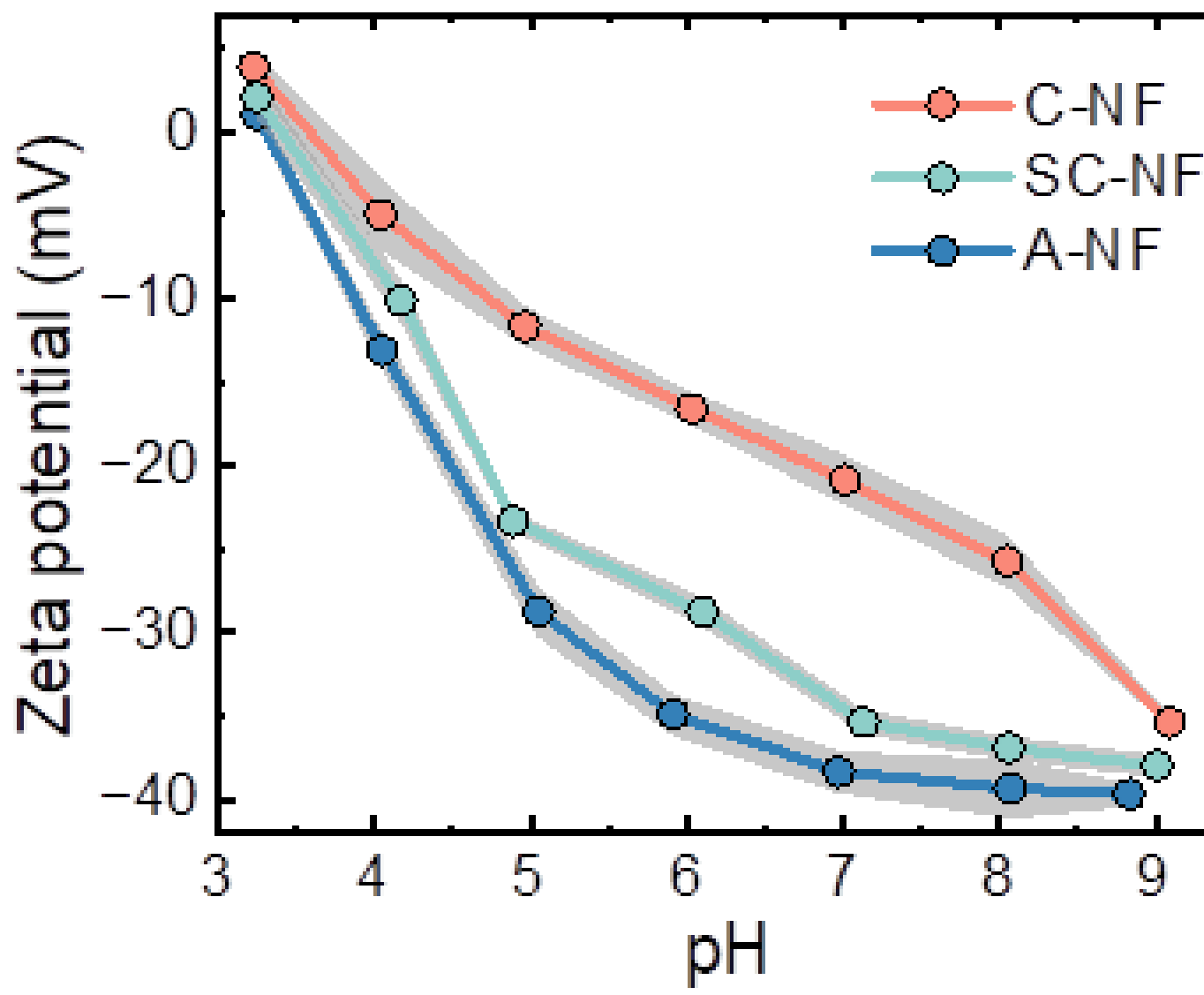

Supplementary Fig. 10: Surface charge characterization. Surface zeta potential of NF membranes prepared at different temperatures. The shaded area represents the standard deviation (s.d., n = 3, n derived from different experimental units) and data are presented as mean values ± s.d.

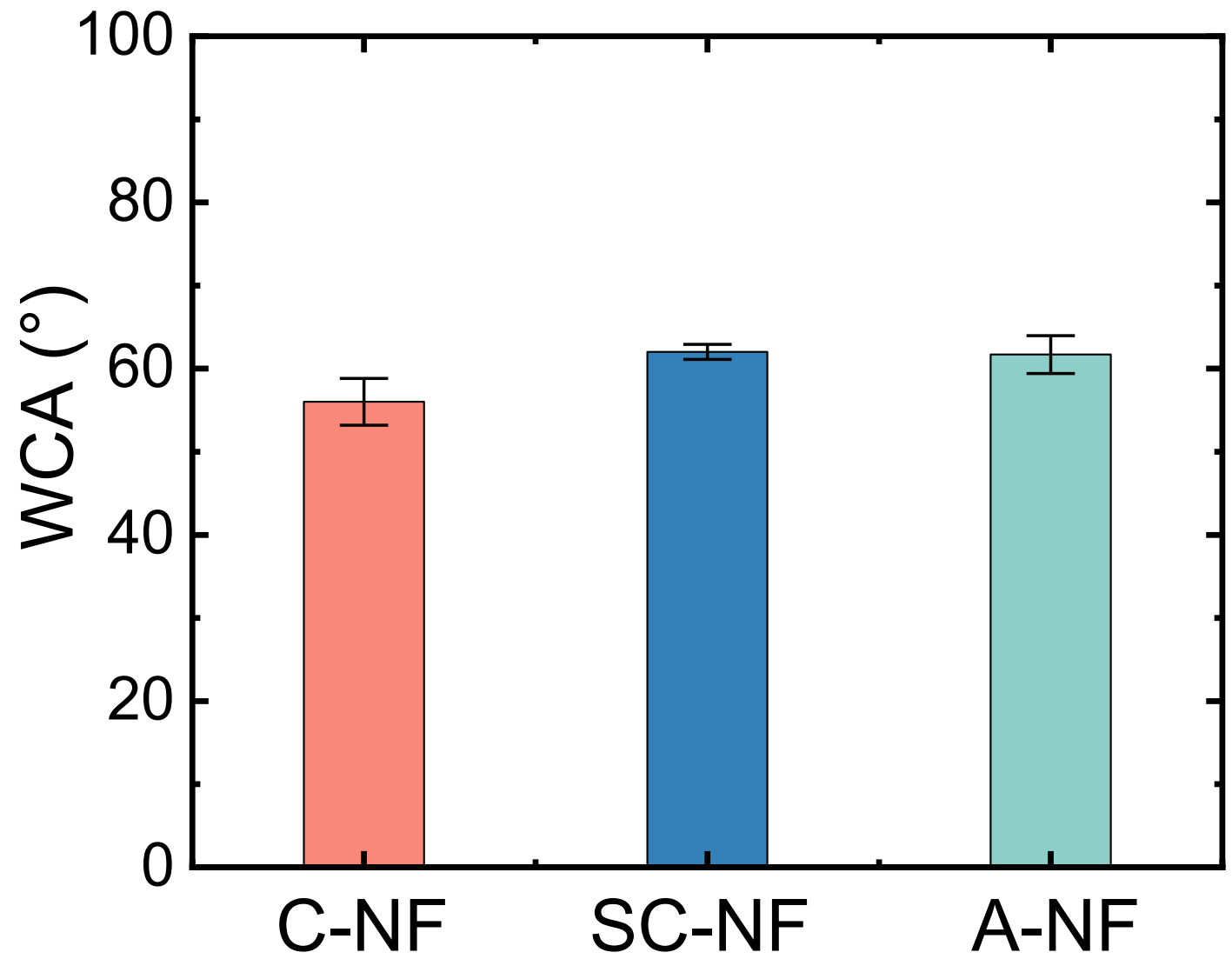

Supplementary Fig. 11: Hydrophilicity characterization. Water contact angle of A-NF, C-NF and SC-NF. Error bars represent the standard deviation (s.d., n = 3, n derived from different experimental units) and data are presented as mean values ± s.d.

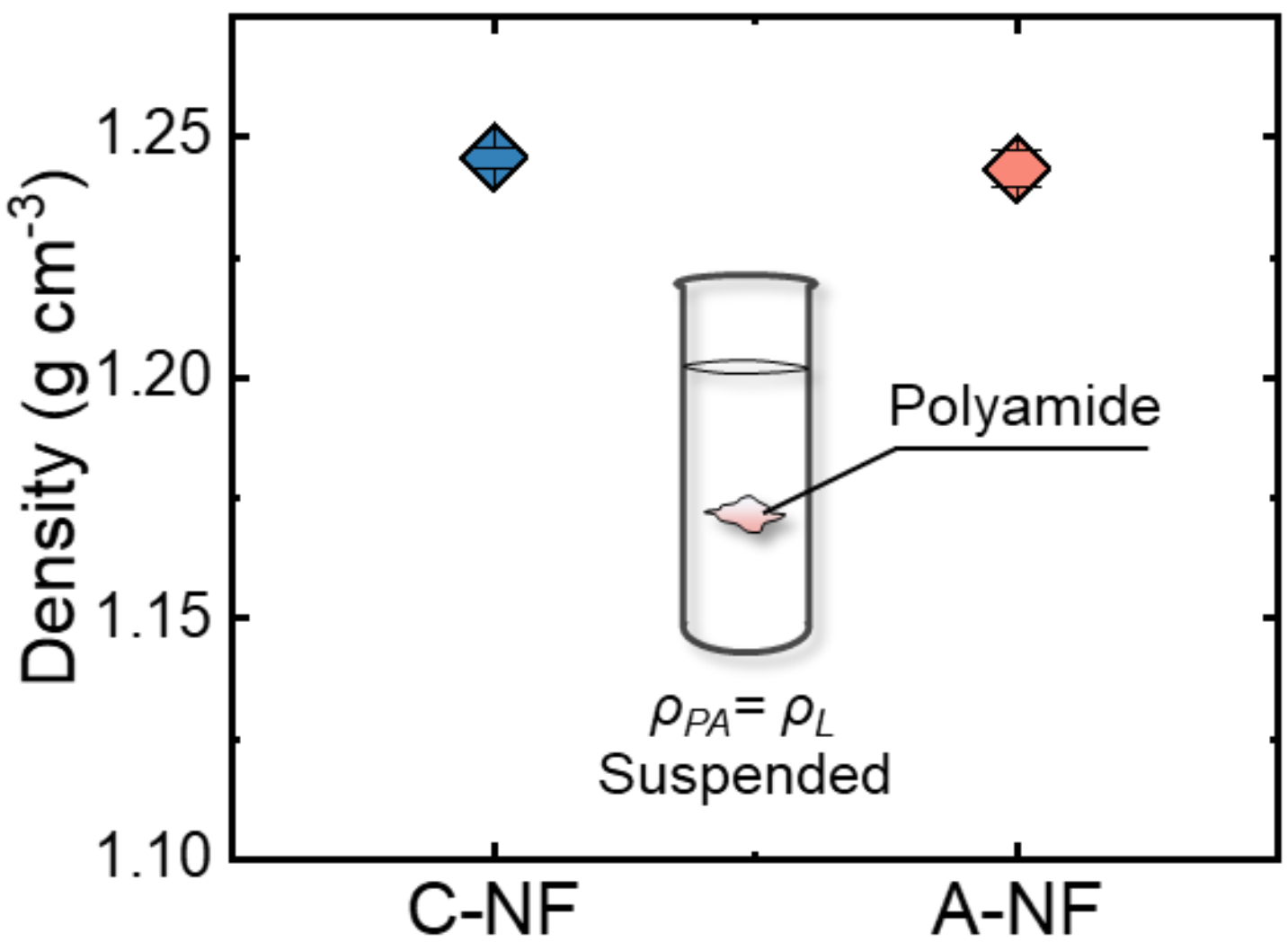

Supplementary Fig. 12: Density characterization. Polyamide layer density of NF membranes prepared at different temperatures. Error bars represent the standard deviation (s.d., n = 3, n derived from different experimental units) and data are presented as mean values ± s.d.

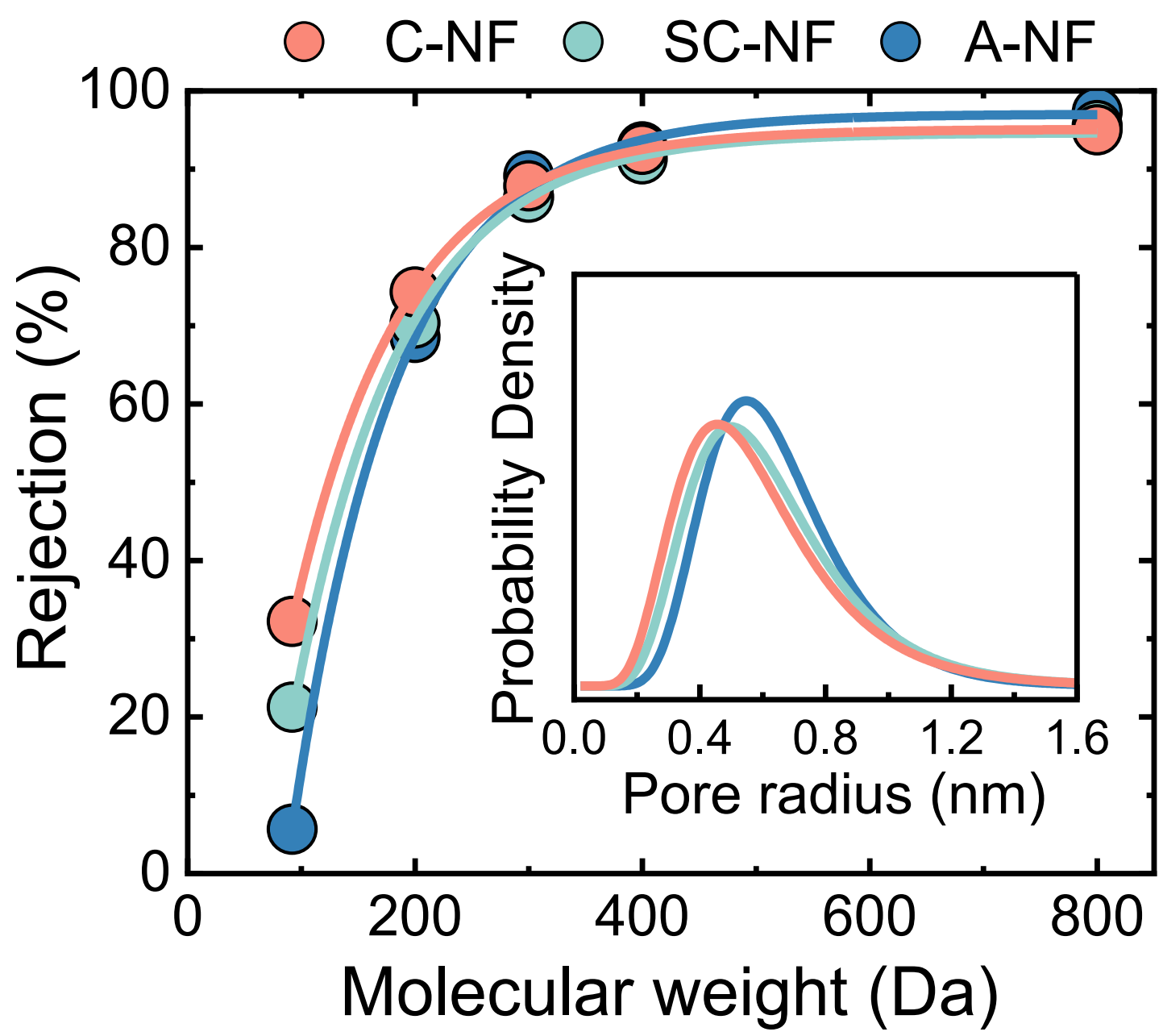

Supplementary Fig. 13: Pore size analysis. Pore size distributions of NF membranes prepared at different temperatures.

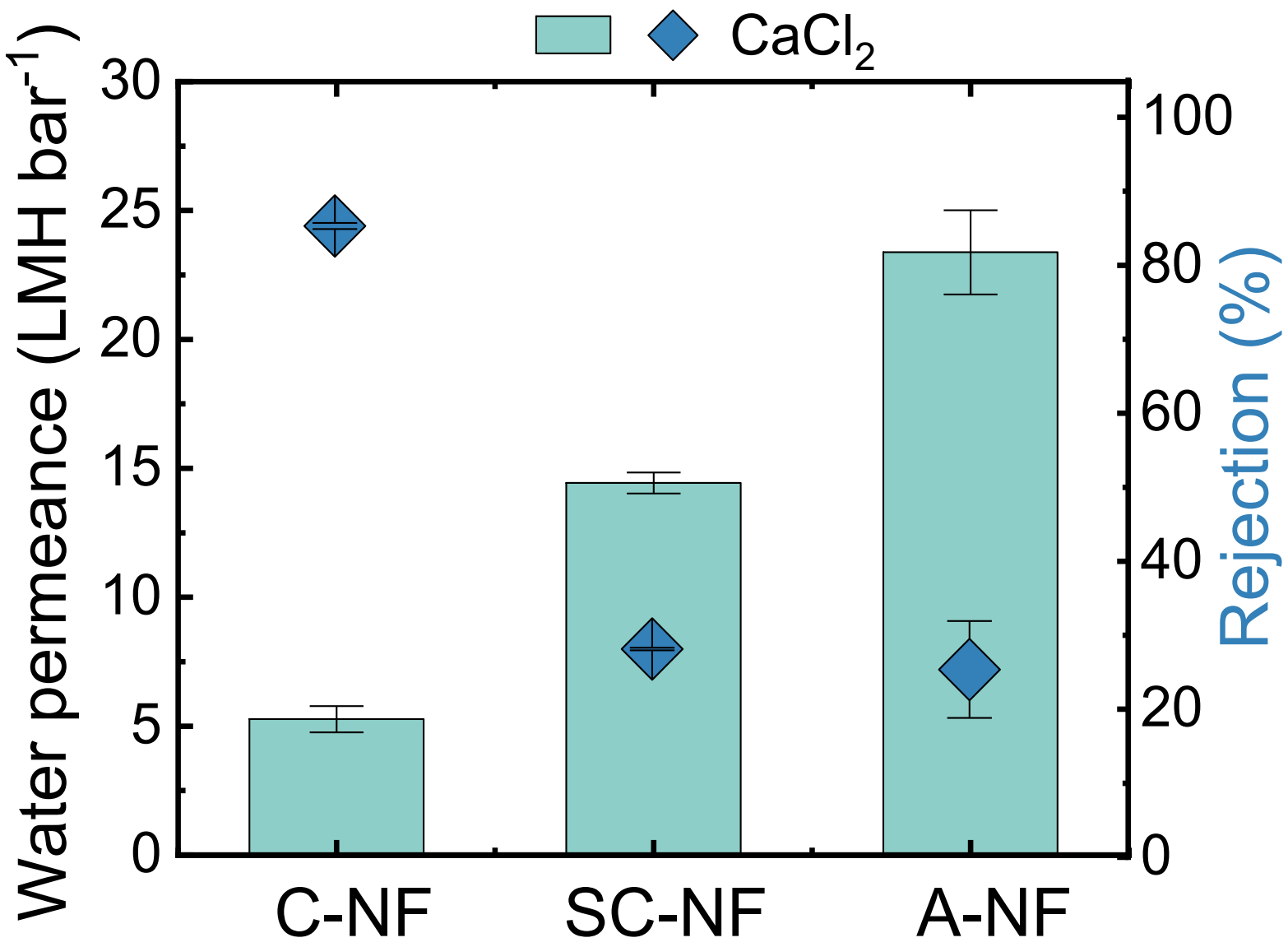

Supplementary Fig. 14: Nanofiltration performance. Effects of interfacial polymerization temperature on permeance and salt rejection performance of NF membranes. Error bars represent the standard deviation (s.d., n = 3, n derived from different experimental units) and data are presented as mean values ± s.d.

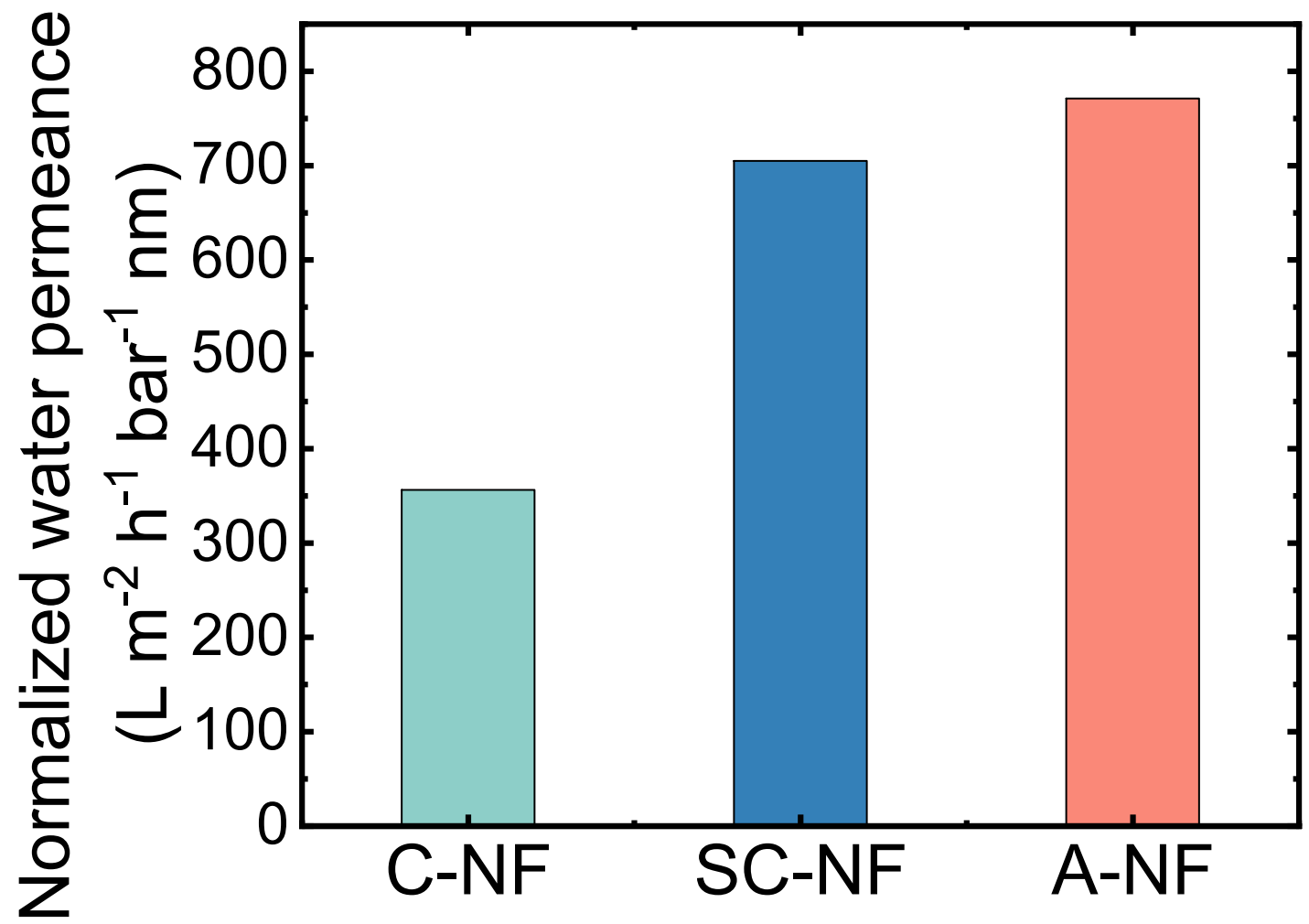

Supplementary Fig. 15: Thickness-normalized water permeance. Thickness-normalized water permeance of C-NF, SC-NF, and A-NF. The water permeance was normalized by multiplying the measured permeance by the corresponding selective-layer thickness.

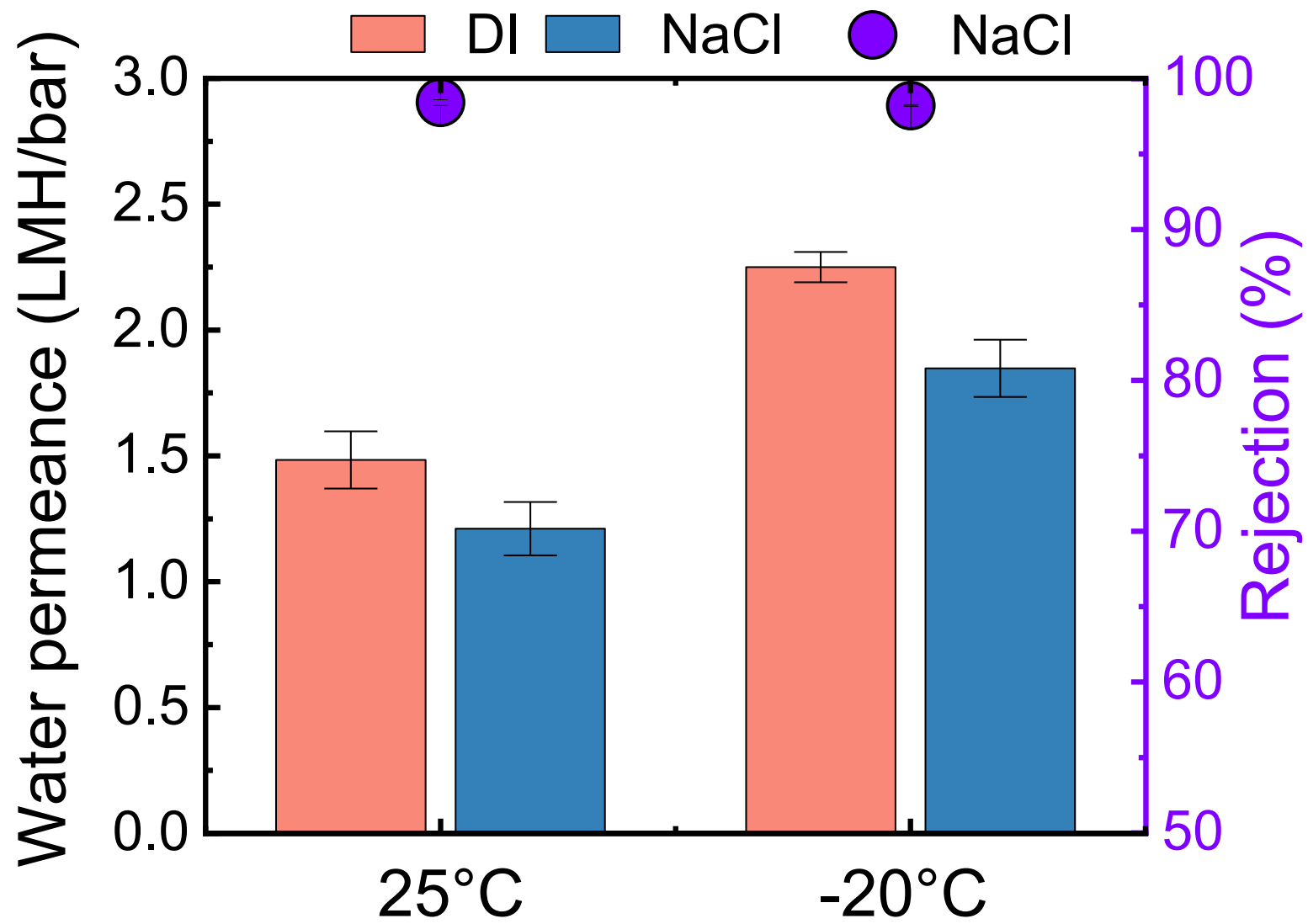

Supplementary Fig. 16: Separation performance of MPD-based membranes. Separation performance of MPD-based nanofiltration membranes prepared by interfacial polymerization at different temperatures (25 °C and −20 °C), including water permeance and NaCl rejection. Applied pressure: 20 Bar, NaCl concentration: 2 g/L. Error bars represent the standard deviation (s.d., n = 3, n derived from different experimental units) and data are presented as mean values ± s.d.

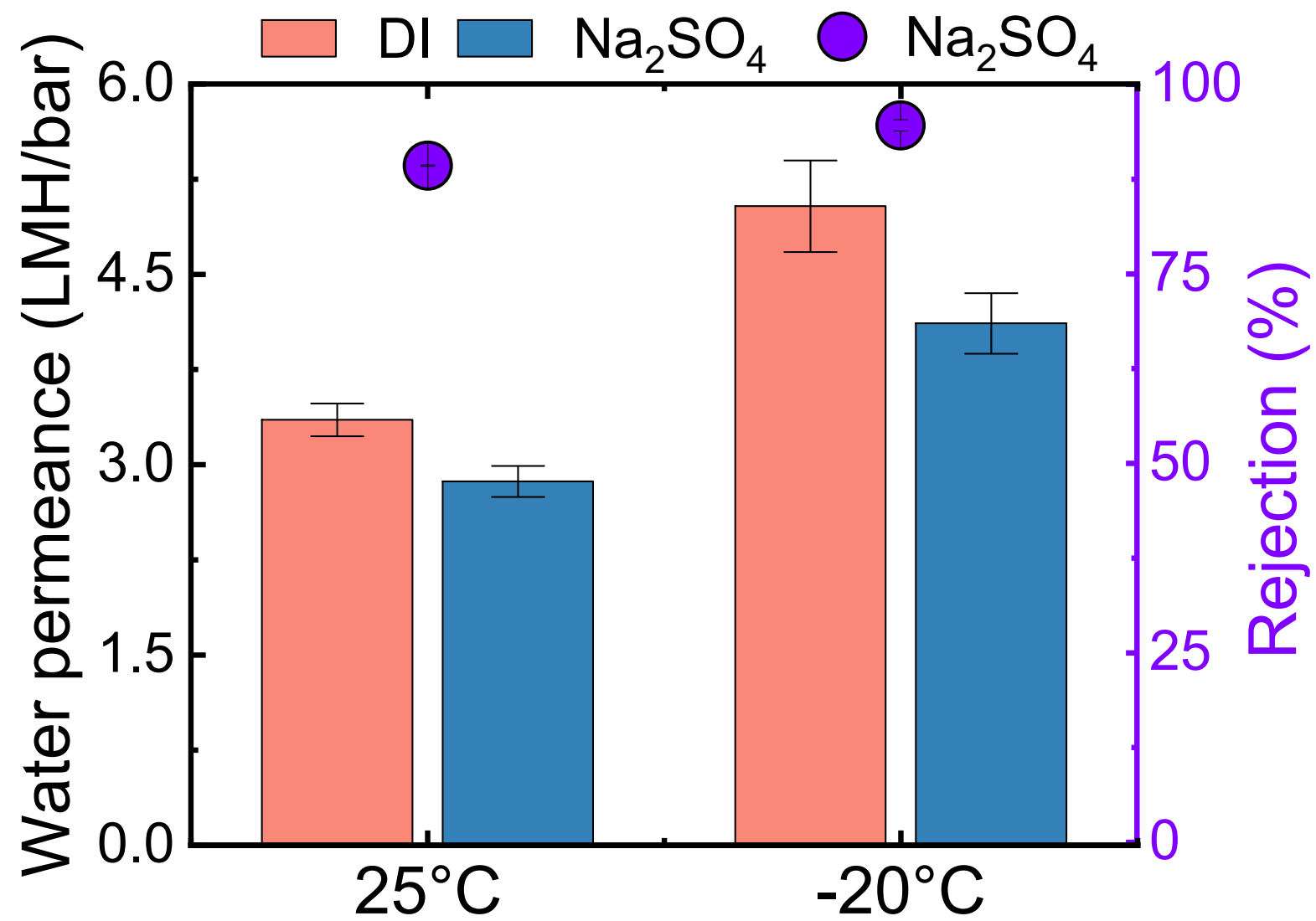

Supplementary Fig. 17: Separation performance of PEI-based membranes.

Separation performance of PEI-based nanofiltration membranes prepared by interfacial polymerization at different temperatures (25 °C and −20 °C), including water permeance and Na_2SO_4 rejection. Applied pressure: 6.9 Bar, Na_2SO_4 concentration: 10mM. Error bars represent the standard deviation (s.d., n = 3, n derived from different experimental units) and data are presented as mean values ± s.d.

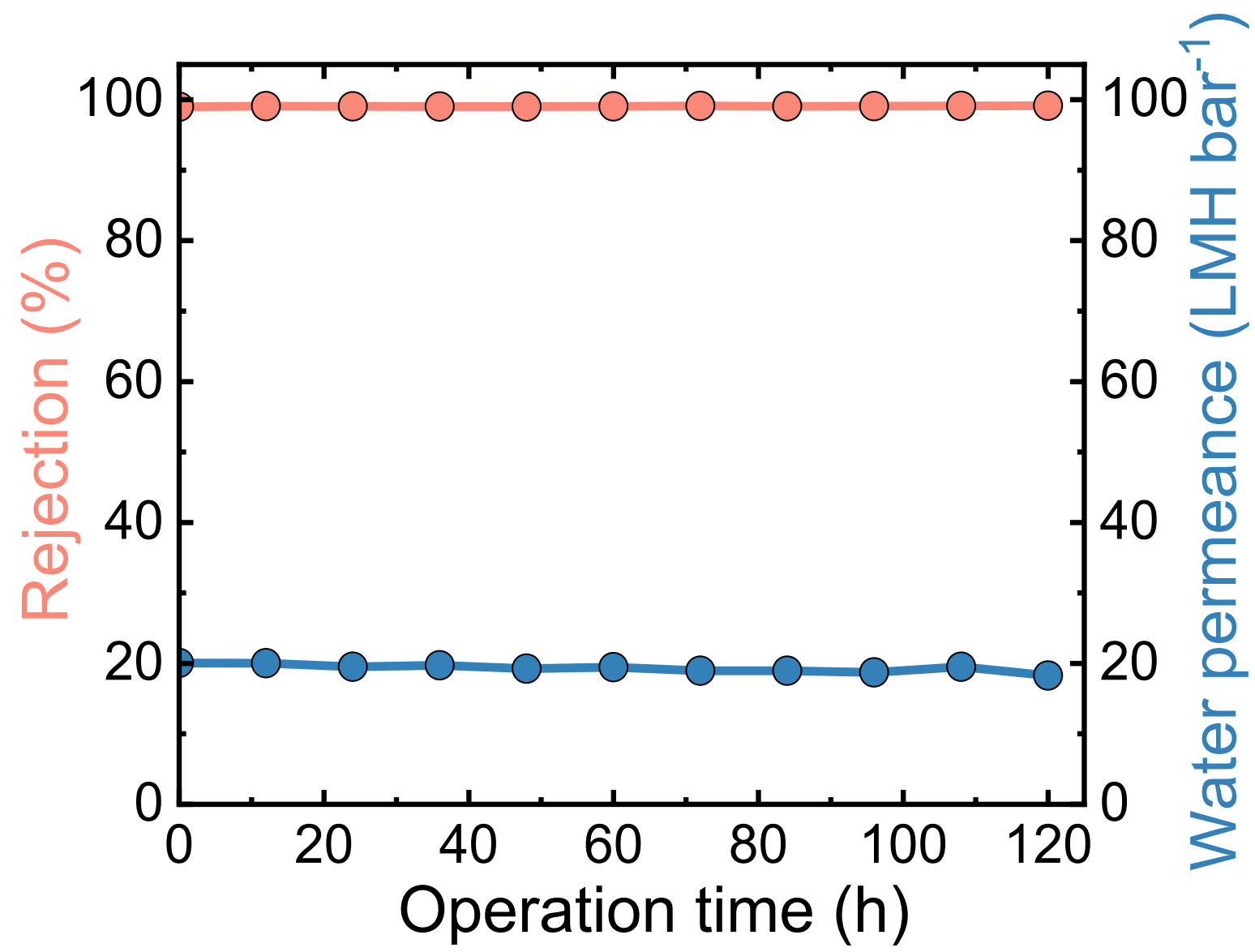

Supplementary Fig. 18: Stability of nanofiltration membranes. Continuous separation performance of A-NF.

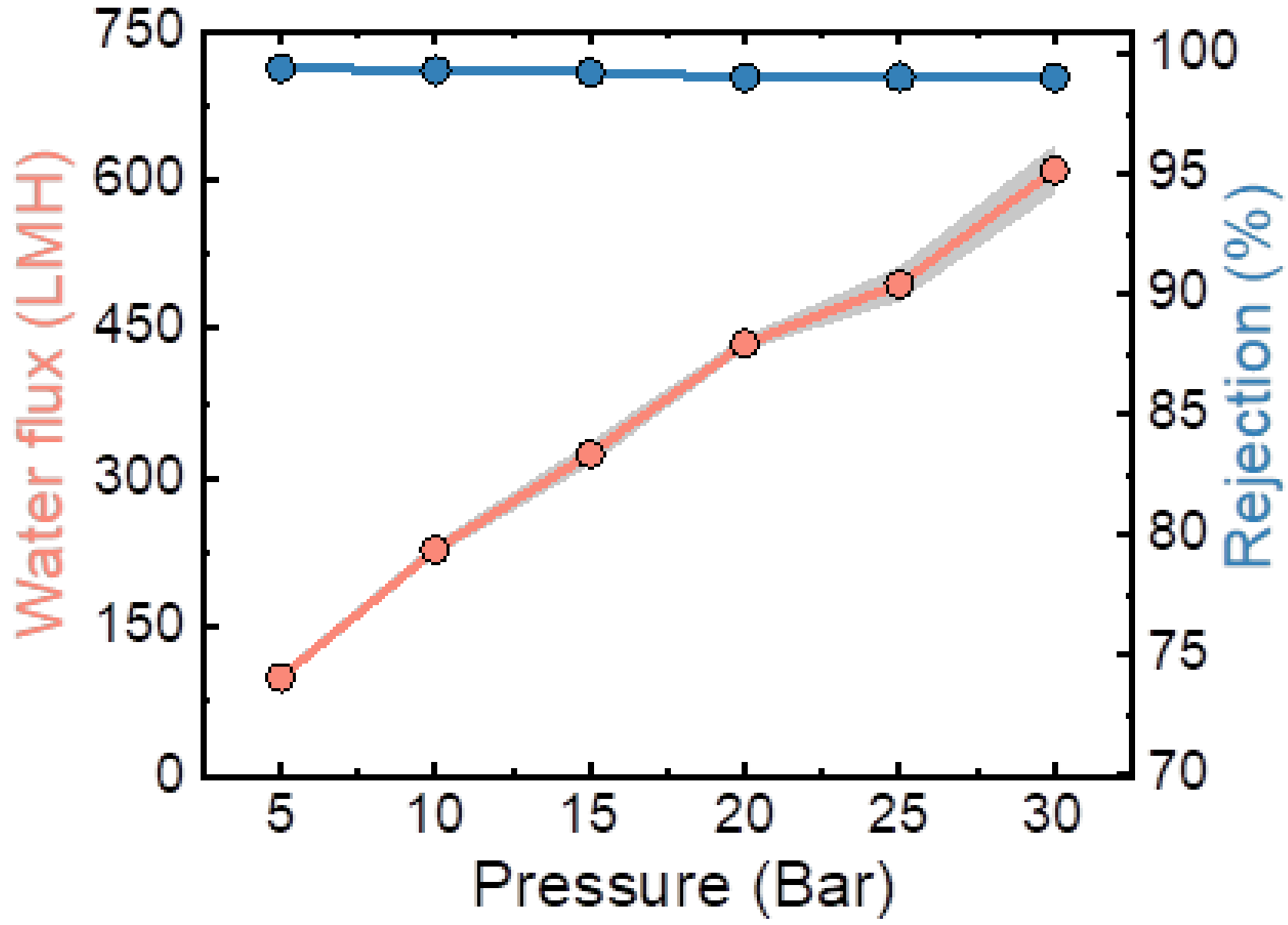

Supplementary Fig. 19: Pressure stability of membrane. Stability of A-NF under different operating pressures. The grey shadow in the water flux data represents the standard deviation (s.d., n = 3, n derived from different experimental units).

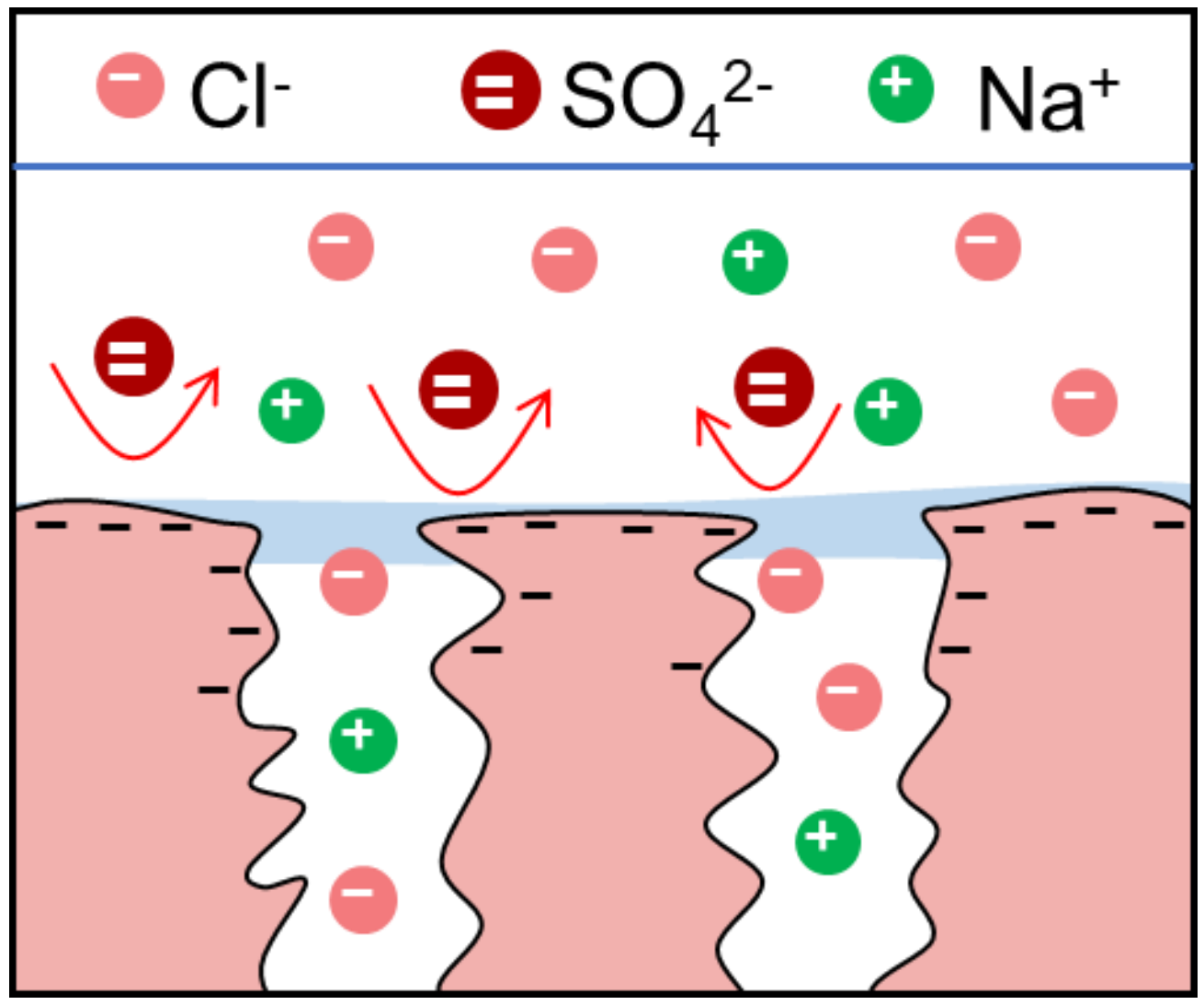

Supplementary Fig. 20: Schematic diagram of co-ion competition effect. The co-ion competition effect enhances SO_4^{2-} retention performance.

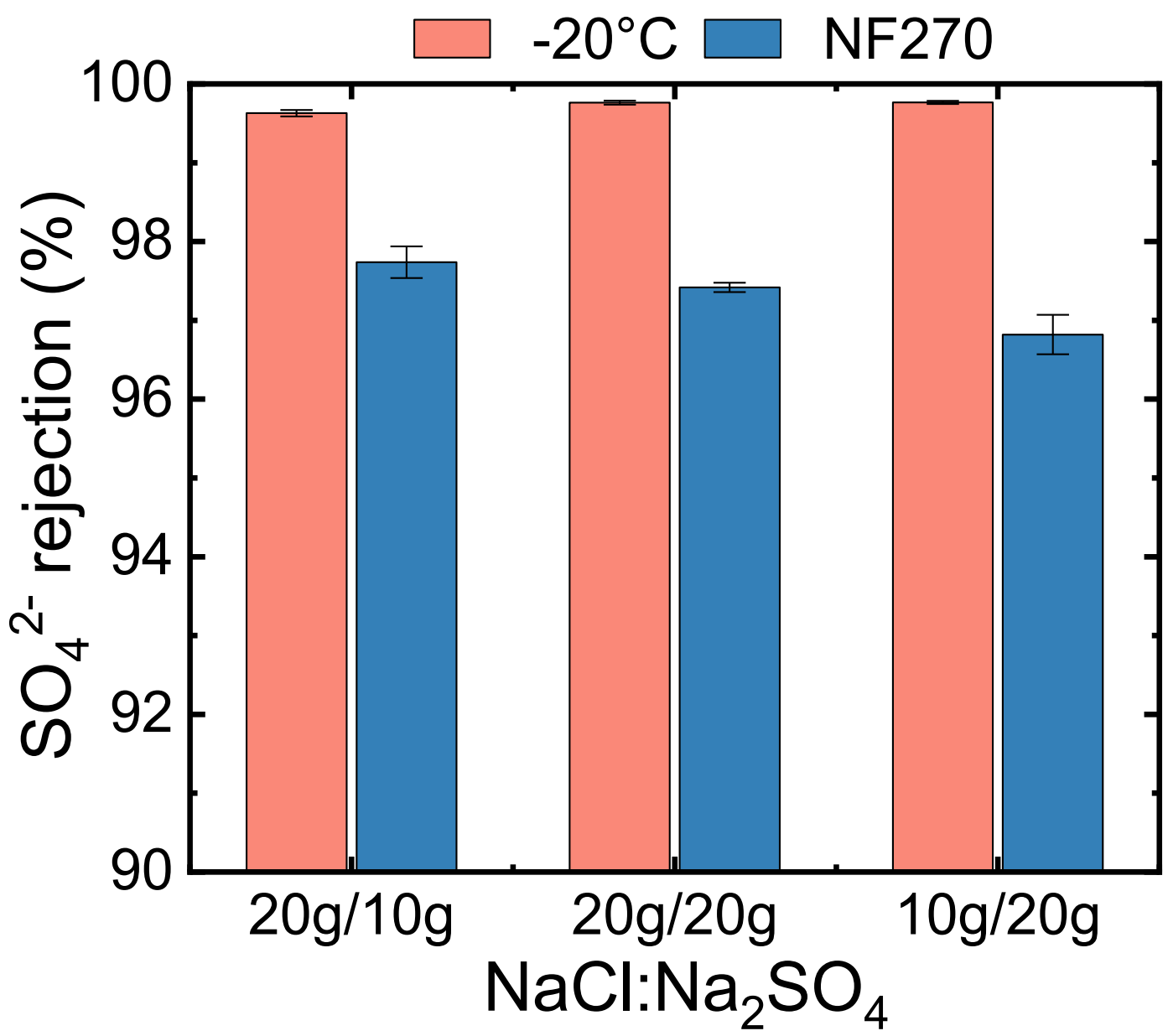

Supplementary Fig. 21: Rejection of SO_4^{2-}. Rejection of SO_4^{2-} by A-NF and NF270 in high-concentration mixed salt separation experiments. Error bars represent the standard deviation (s.d., n = 3, n derived from different experimental units) and data are presented as mean values ± s.d.

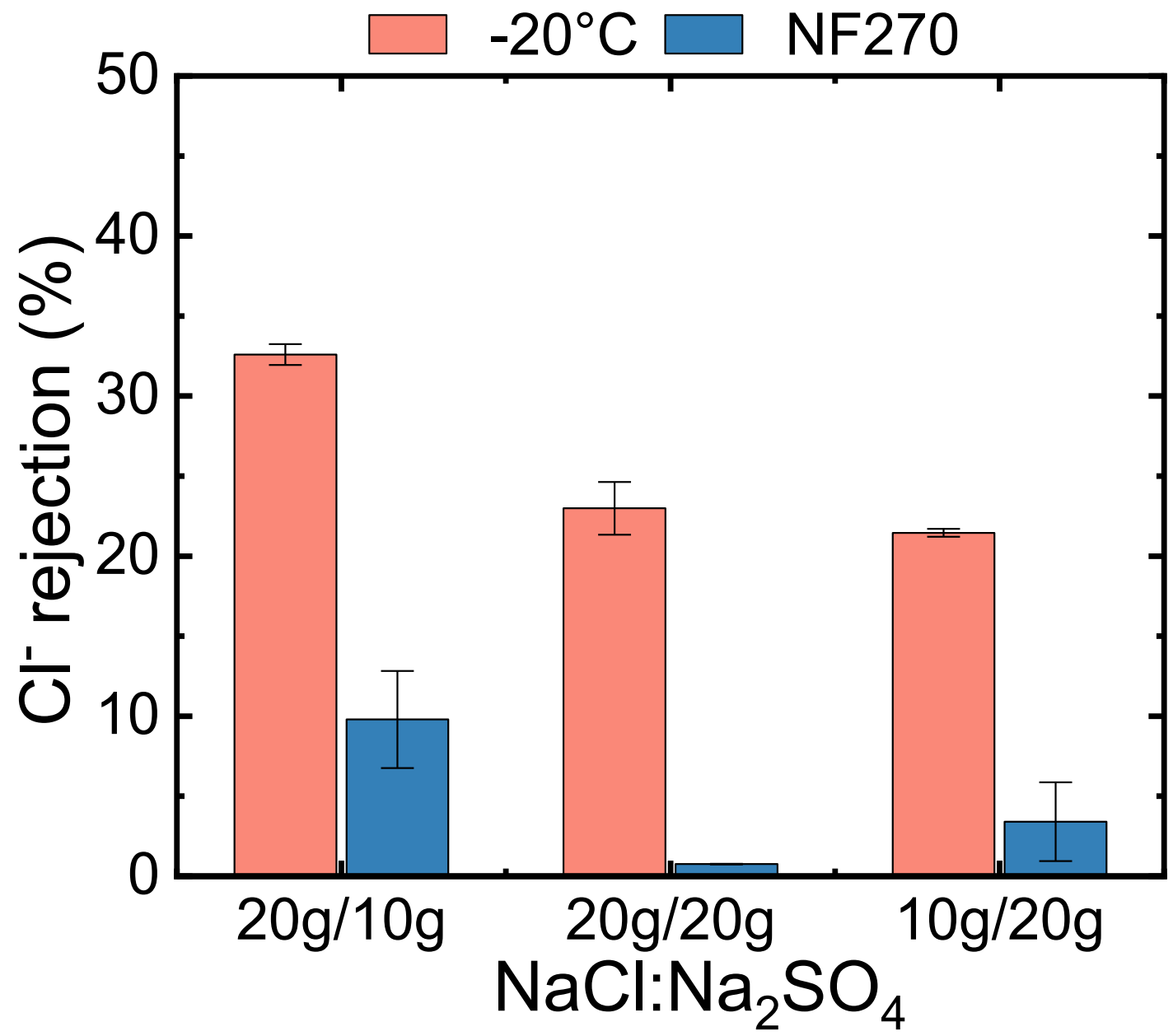

Supplementary Fig. 22: Rejection of Cl^-. Rejection of Cl^- by A-NF and NF270 in high-concentration mixed salt separation experiments. Error bars represent the standard deviation (s.d., n = 3, n derived from different experimental units) and data are presented as mean values ± s.d.

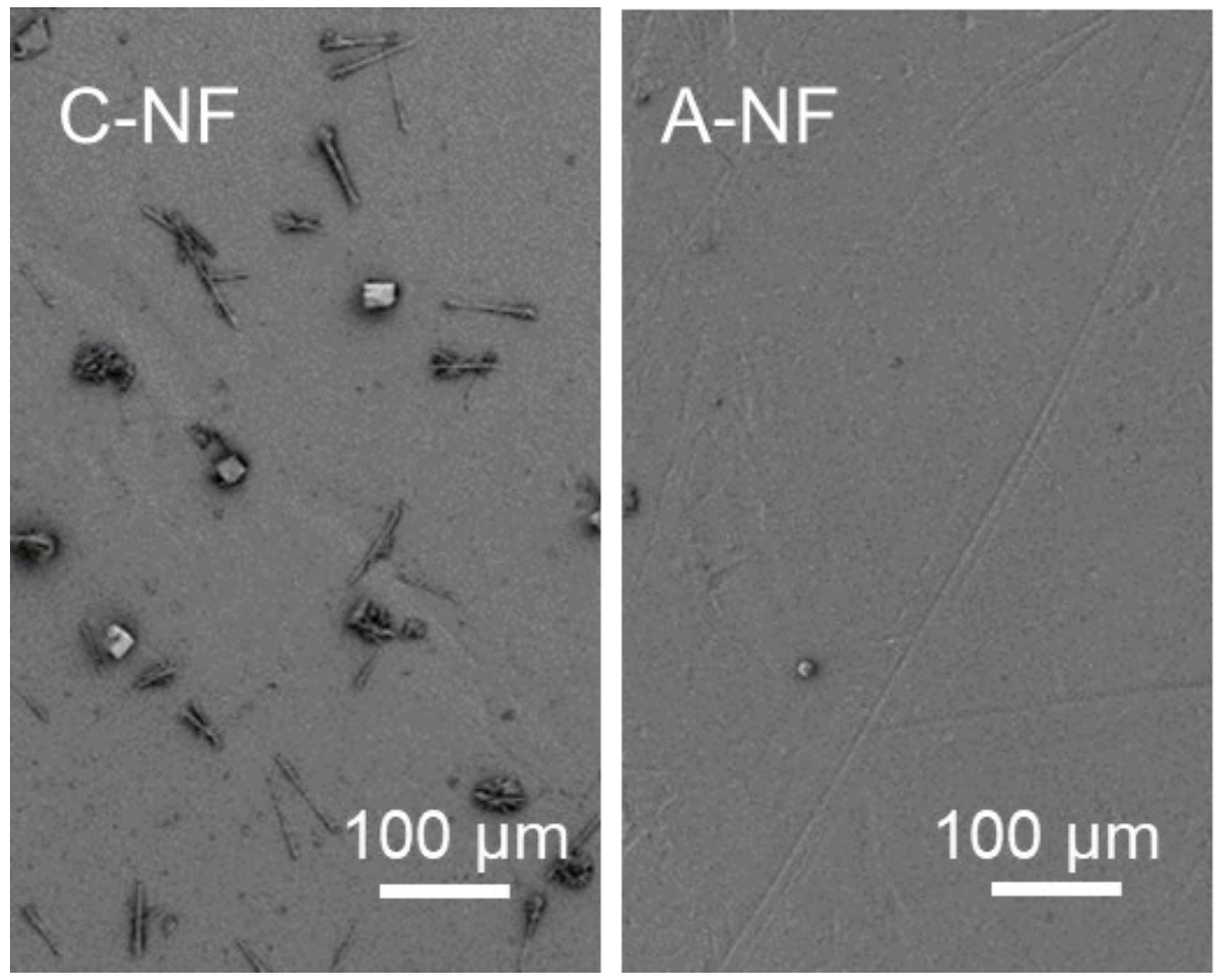

Supplementary Fig. 23: Surface morphology after anti-scaling test. Surface SEM images of C-NF and A-NF after long-term anti-scaling test.

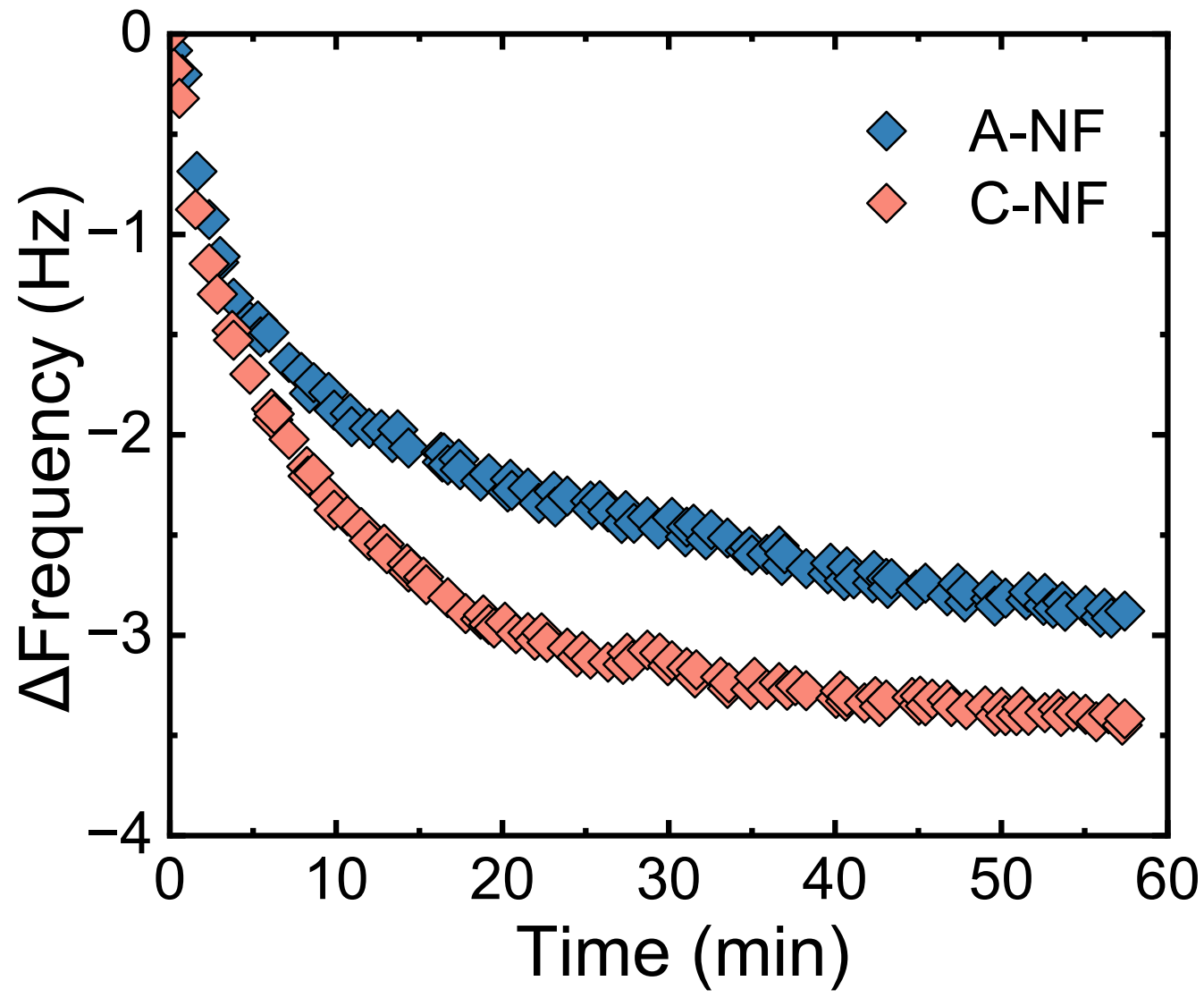

Supplementary Fig. 24: QCM analysis. Real time in-situ monitoring of humic acid deposition (i.e., fouling) using QCM.

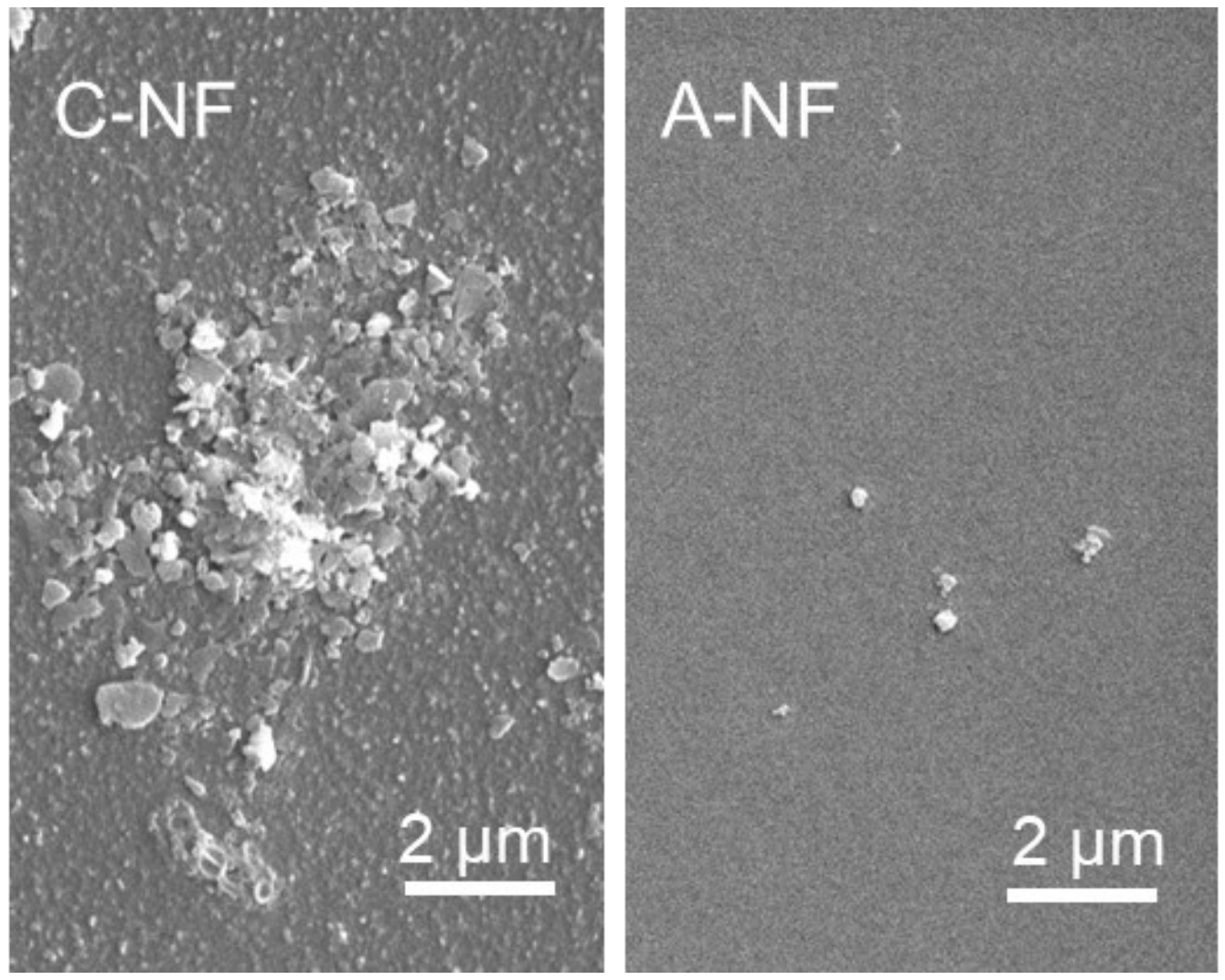

Supplementary Fig. 25: Surface morphology after anti-fouling test. Surface SEM images of C-NF and A-NF after long-term anti-fouling test.

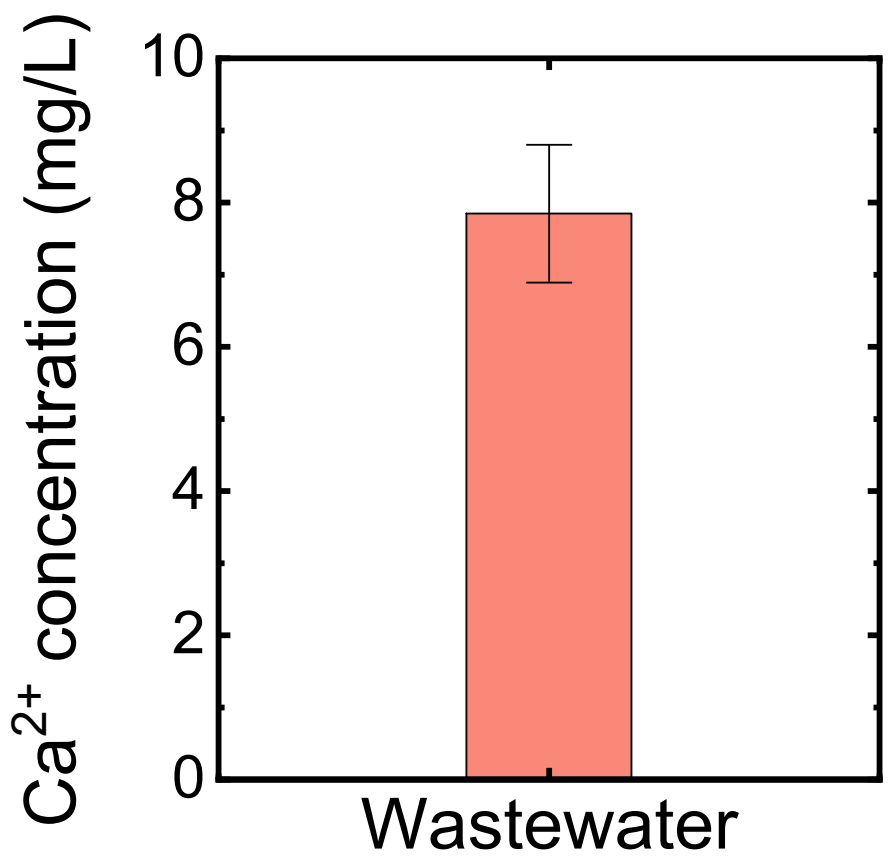

Supplementary Fig. 26: Ca^{2+} concentration. The Ca^{2+} concentration in the actual coal chemical wastewater used in this study. Error bars represent the standard deviation (s.d., n = 3, n derived from different experimental units) and data are presented as mean values ± s.d.

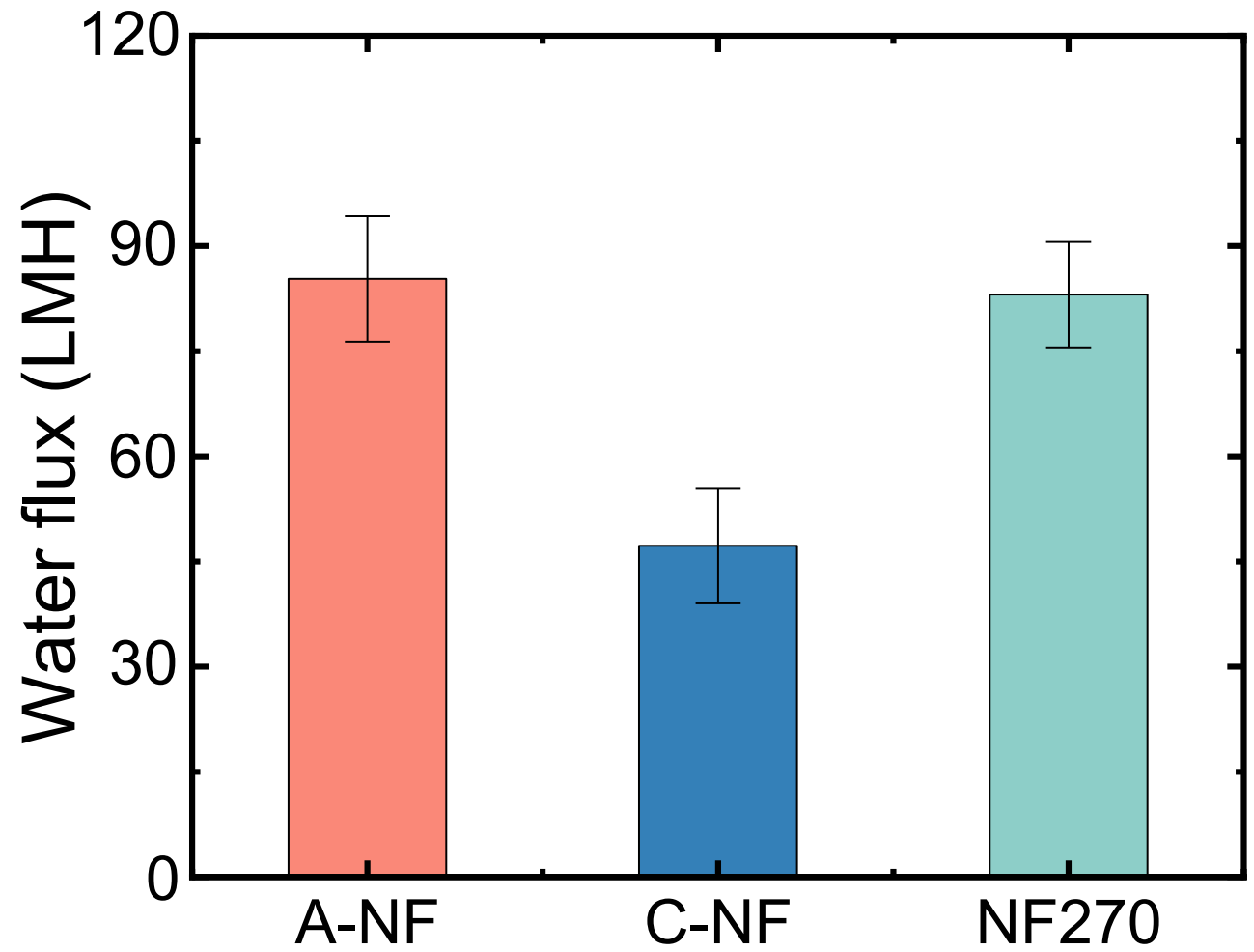

Supplementary Fig. 27: Nanofiltration performance under actual wastewater treatment. Water flux in the separation of actual wastewater by A-NF, C-NF, and NF270. Error bars represent the standard deviation (s.d., n = 3, n derived from different experimental units) and data are presented as mean values ± s.d.

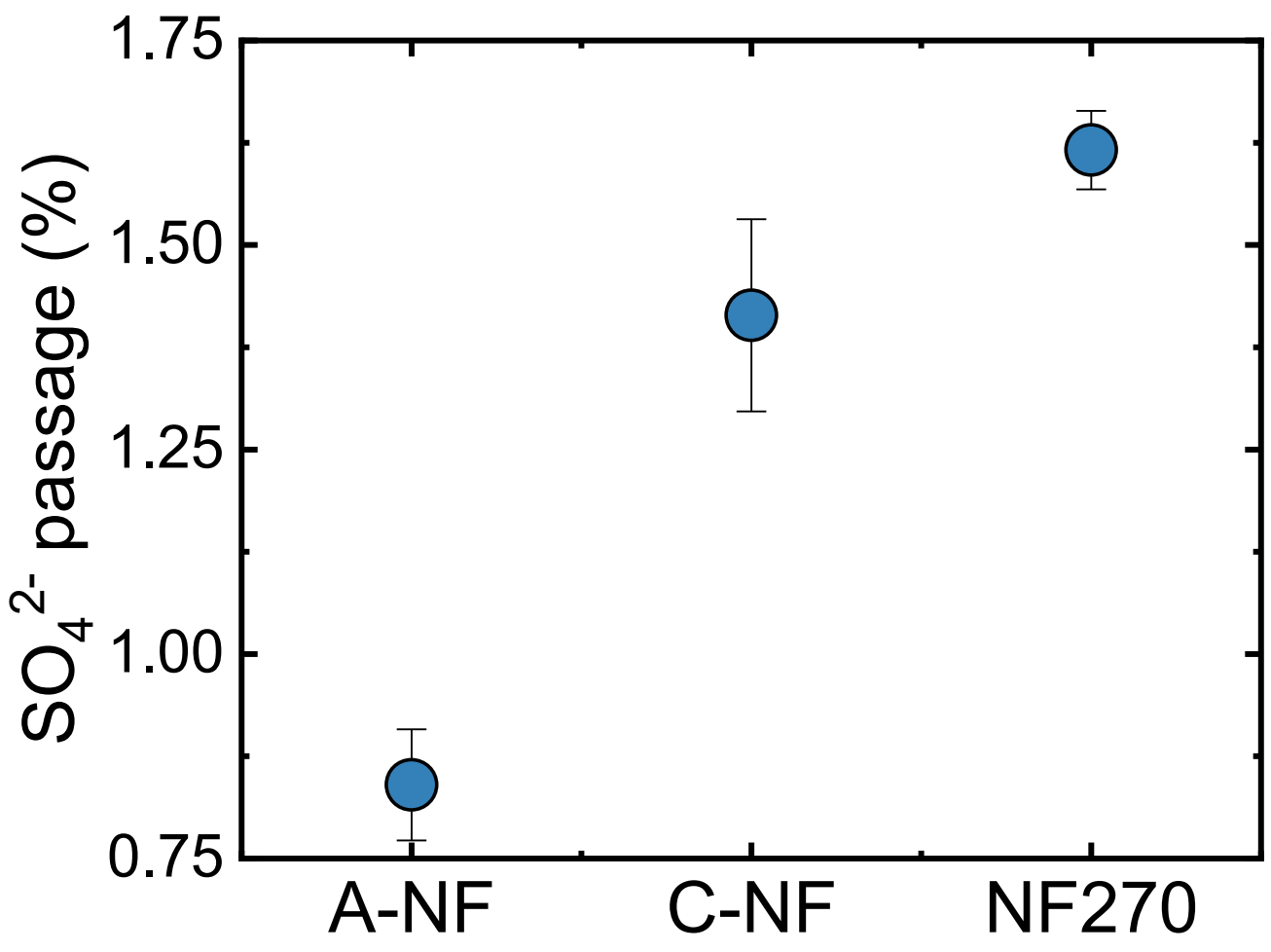

Supplementary Fig. 28: SO_4^{2-} passage under actual wastewater treatment. SO_4^{2-} passage during the separation of actual wastewater by A-NF, C-NF, and NF270. Error bars represent the standard deviation (s.d., n = 3, n derived from different experimental units) and data are presented as mean values ± s.d.

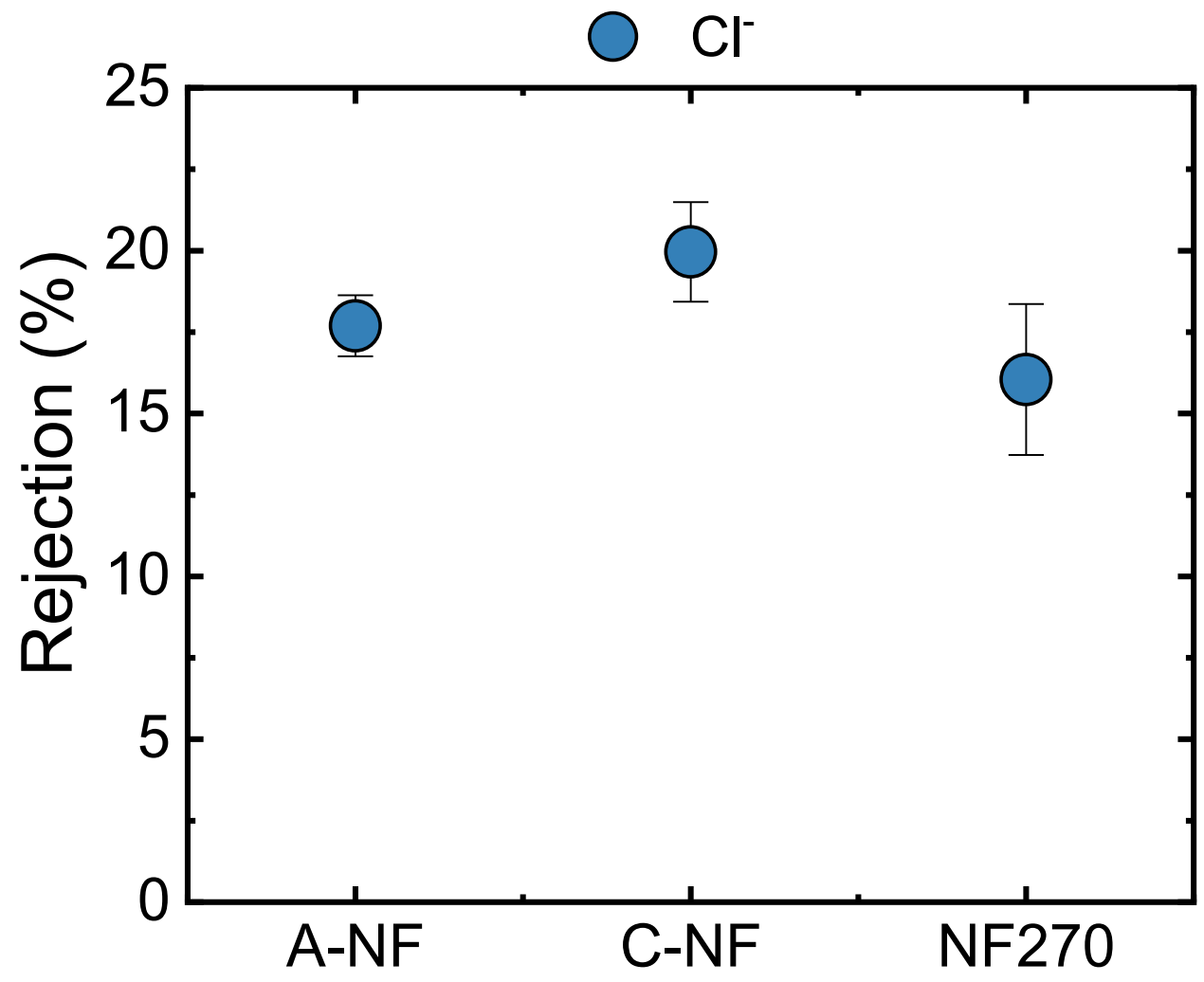

Supplementary Fig. 29: Rejection of Cl^- under actual wastewater treatment.
Rejection of Cl^- by A-NF, C-NF and NF270 in actual wastewater. Error bars represent the standard deviation (s.d., n = 3, n derived from different experimental units) and data are presented as mean values ± s.d.

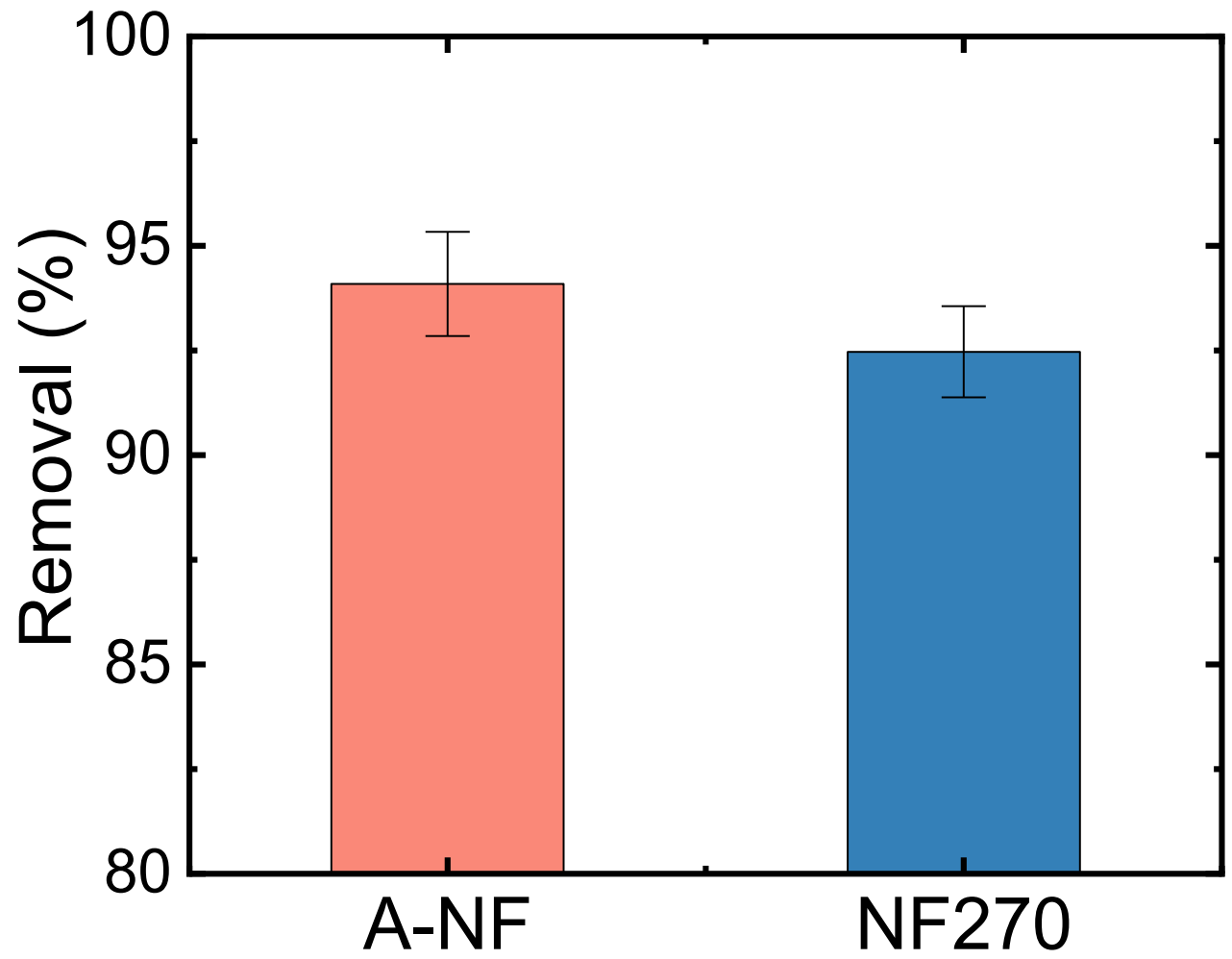

Supplementary Fig. 30: TOC removal performance. Rejection of TOC by A-NF and NF270 in actual wastewater. Error bars represent the standard deviation (s.d., n = 3, n derived from different experimental units) and data are presented as mean values ± s.d.

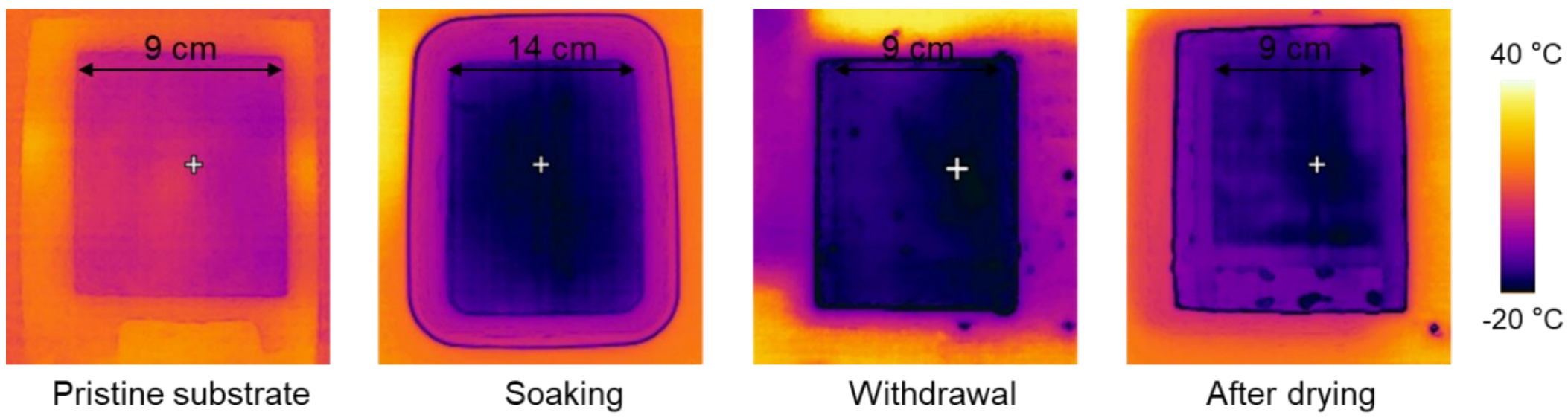

Supplementary Fig. 31: Temperature changes of the membrane substrate. Infrared thermal images of the membrane substrate at different stages of the ultralow-temperature treatment

Supplementary Tables

Supplementary Table 1. Comparison of selectivity and permeance data from the literature with this work.

Source	*Specific flux* ($Lm^{-2}h^{-1}bar^{-1}$)	R_{NaCl} (%)	R_{Na2SO4} (%)	Seletivity
This work (A-NF)	24.1	38.86	99.61	158.65
This work (C-NF)	15	62.60	99.09	47.93
This work (SC-NF)	5.48	48.23	98.92	41
https://doi.org/10.1016/j.seppur.2020.116528	2.1	90.0	99.7	39.67
https://pubs.acs.org/doi/10.1021/acsnano.5b02598	18.8	/	/	93
https://doi.org/10.1002/aic.17419	13.34	13.6	98.9	98.4
	6.84	12.4	97.5	65.6
	4.73	46.5	98.9	72.2
https://doi.org/10.1021/acs.est.4c04063	16.5	/	99.1	91
http://dx.doi.org/10.1016/j.memsci.2016.10.011	16.4	32.0	97.3	25.2
https://doi.org/10.1016/j.memsci.2019.117386	18.5	16.1	97.8	38.1
https://doi.org/10.1016/j.memsci.2024.123085	38.3	16.0	99.1	83
https://doi.org/10.1038/s41467-023-43291-2	13.6	/	/	29.2
https://doi.org/10.1038/s41467-022-35681-9	41.7	34.4	98.7	50
https://doi.org/10.1073/pnas.2019891118	26.5	10.8	96.6	26.23
https://doi.org/10.1038/s41467-022-28183-1	38.9	25.2	94.9	14.67
https://doi.org/10.1016/j.memsci.2021.119073	19.5	-20.1	99.1	133.4
https://doi.org/10.1002/adfm.202313026	42.9	18.79	97.78	36.58
https://doi.org/10.1126/science.adi9531	29.7	42.5	99.6	143.8
https://doi.org/10.1016/j.colsurfa.2021.127001	35.67	15.6	98.2	46.9
https://doi.org/10.1002/adfm.202007054	37.6	16.2	98.94	79.06
https://doi.org/10.1016/j.memsci.2020.118064	1.6	8.4	83.4	5.51
	2.7	37.5	86.7	4.70
https://doi.org/10.1002/smll.201601253	32.0	22.0	95.9	19.02
https://doi.org/10.1016/j.desal.2017.10.011	1.1	92.5	98.0	3.75
	1.0	93.0	96.8	2.19
	1.2	91.0	94.0	1.50

https://doi.org/10.1016/j.cej.2018.03.116	4.1	40.0	95.8	14.29
https://doi.org/10.1038/s41467-018-04467-3	53.5	10.0	95.3	19.15
https://doi.org/10.1021/acs.est.8b02425	19.6	18.0	95.0	16.40
https://doi.org/10.1021/acsnano.8b09761	50.3	13.4	96.5	24.74
https://doi.org/10.1021/acsami.8b18719	21.0	18.8	98.5	54.13
https://doi.org/10.1016/j.memsci.2019.01.040	16.4	14.6	97.2	30.50
	20.7	19.6	93.4	12.18
https://doi.org/10.1016/j.cej.2019.123993	9.7	35.0	55.0	1.44
https://doi.org/10.1002/smll.201601253	32.0	22.0	95.9	19.02
https://doi.org/10.1039/C8TA08273A	28.0	50.0	96.0	12.50
https://doi.org/10.1016/j.memsci.2019.117192	33.7	14.3	98.1	45.11
https://doi.org/10.1039/C9TA12984G	52.8	16.0	96.4	23.33
	62.9	10.0	93.5	13.85
https://doi.org/10.1016/j.memsci.2020.117971	16.6	30.0	95.5	15.56
https://doi.org/10.1039/C9TA02299F	30.8	21.3	97.5	31.48
https://doi.org/10.1039/C9EW00227H	34.5	12.3	97.8	39.86
https://doi.org/10.1039/C9SC03088C	31.1	12.0	95.0	17.60
https://doi.org/10.1016/j.memsci.2017.09.016	23.0	14.7	92.0	10.66
https://doi.org/10.1016/j.memsci.2019.117192	33.7	14.3	98.1	45.11
https://doi.org/10.1021/acsomega.9b01446	10.8	48.0	99.0	52.00
https://doi.org/10.1039/C9TA08163A	48.7	27.3	94.3	12.75
https://doi.org/10.1039/C9TA12258C	45.2	25.7	99.3	106.14
https://doi.org/10.1021/acs.est.0c03589	19.3	20.5	90.3	8.21
	14.8	24.2	97.7	32.24
	10.7	15.9	94.7	15.98
https://doi.org/10.1039/D0TA06520J	27.6	25.0	95.0	15.00
	48.9	26.0	93.9	12.13
https://doi.org/10.1016/j.memsci.2020.118417	19.1	22.0	94.0	13.00
	21.5	23.5	95.0	15.30
https://doi.org/10.1021/acs.est.9b06779	31.4	48.0	99.4	86.67
https://doi.org/10.1016/j.memsci.2020.118282	42.3	11.0	96.5	25.43
https://doi.org/10.1016/j.memsci.2019.117444	33.0	14.0	95.0	17.20
https://doi.org/10.1016/j.memsci.2020.117874	63.0	8.1	97.1	31.69
https://doi.org/10.1021/acsami.1c02776	48.4	12.6	96.0	21.70

	27.2	41.3	95.6	13.24
https://doi.org/10.1016/j.memsci.2021.119154	9.6	37.0	91.0	7.00
https://doi.org/10.1016/j.seppur.2021.118802	21.0	21.5	98.4	49.06
https://doi.org/10.1016/j.desal.2020.114802	34.0	7.4	95.0	18.52
https://doi.org/10.1016/j.memsci.2013.04.018	7.0	44.1	99.0	55.90
https://doi.org/10.1016/j.memsci.2012.10.053	5.4	30.0	80.0	3.50
https://doi.org/10.1016/j.memsci.2012.10.042	3.7	42.5	79.5	2.80
https://doi.org/10.1016/j.memsci.2015.04.020	13.3	10.0	98.9	81.82
	10.8	16.0	99.1	93.33
https://doi.org/10.1039/C4RA14854A	2.5	53.9	51.3	0.95
	2.3	57.2	46.4	0.80
https://doi.org/10.1016/j.memsci.2015.12.031	17.5	12.0	94.0	14.67
https://doi.org/10.1016/j.desal.2016.03.007	2.4	59.5	95.2	8.44
https://doi.org/10.1039/C6RA05716K	6.4	35.2	92.0	8.10
https://doi.org/10.1039/C6RA16896E	3.9	46.0	90.0	5.40
https://doi.org/10.1039/C5TA09252C	7.5	18.9	96.4	22.53
https://doi.org/10.1016/j.memsci.2017.05.062	9.5	25.6	97.3	27.56
https://doi.org/10.1016/j.memsci.2017.06.061	11.5	38.0	91.0	6.89
https://doi.org/10.1016/j.carbon.2016.07.056	6.6	24.0	65.0	2.17
https://doi.org/10.1016/j.seppur.2015.09.039	6.1	36.6	93.6	9.86
https://doi.org/10.1016/j.desal.2017.06.006	5.5	30.1	85.0	4.66
https://doi.org/10.1016/j.memsci.2017.03.040	5.6	32.0	98.8	56.67
https://doi.org/10.1039/C7RA11550D	7.0	34.5	97.0	21.83
	8.2	34.0	97.6	27.50
https://doi.org/10.1016/j.memsci.2017.05.075	9.7	26.0	88.0	6.17
	11.2	28.0	85.0	4.80
https://doi.org/10.1021/acs.iecr.8b04515	7.8	40.0	98.0	30.00
https://doi.org/10.3390/polym10111253	8.0	43.0	98.0	28.50
https://doi.org/10.1016/j.memsci.2017.09.075	8.3	35.0	98.4	40.63
https://doi.org/10.1021/acs.est.8b04102	16.5	22.7	99.2	96.63
https://doi.org/10.1016/j.memsci.2018.07.031	10.4	50.0	95.0	10.00
https://doi.org/10.1038/s41598-018-23188-7	2.0	94.0	97.0	2.00
https://doi.org/10.1016/j.memsci.2018.07.044	6.1	16.0	94.0	14.00
	5.3	27.5	92.0	9.06

	7.0	10.0	94.0	15.00
https://doi.org/10.1016/j.memsci.2018.03.034	20.5	5.2	21.7	1.21
https://doi.org/10.1016/j.seppur.2018.08.037	9.7	42.6	99.0	57.40
https://doi.org/10.1016/j.cej.2018.10.147	20.3	22.0	98.0	39.00
https://doi.org/10.1021/acs.est.9b05343	39.5	23.0	10.0	0.86
https://doi.org/10.1016/j.arabjc.2017.12.009	4.1	37.4	97.0	20.87
https://doi.org/10.1016/j.seppur.2018.09.034	2.3	92.1	97.2	2.77
https://doi.org/10.1016/j.apsusc.2019.05.157	11.9	37.0	98.0	31.50
	12.4	39.0	98.0	30.50
https://doi.org/10.1016/j.cej.2019.02.180	14.3	45.0	98.0	27.50
https://doi.org/10.1016/j.memsci.2019.117203	21.7	13.0	87.6	7.02
https://doi.org/10.1016/j.memsci.2019.05.065	11.7	21.5	95.0	15.70
https://doi.org/10.1016/j.seppur.2019.05.018	9.7	44.0	98.0	28.00
	13.3	35.0	97.0	21.67
https://doi.org/10.1016/j.memsci.2019.117717	20.5	20.0	94.5	14.55
https://doi.org/10.1039/C8TA03446J	13.6	13.0	68.9	2.80
https://doi.org/10.1016/j.memsci.2017.11.057	10.6	31.6	97.8	31.09
https://doi.org/10.1021/acs.iecr.9b00772	12.7	39.8	99.1	66.89
	14.6	38.1	99.0	61.90
	13.1	35.4	98.9	58.73
https://doi.org/10.1039/C9TA02299F	18.0	30.2	98.4	43.63
	21.3	25.6	98.2	41.33
	26.8	22.6	97.7	33.65
	30.8	21.3	97.5	31.48
https://doi.org/10.1039/C9EW00227H	34.5	12.3	97.8	39.86
https://doi.org/10.1021/acsapm.9b00973	9.8	25.1	95.7	17.42
	8.0	31.7	99.6	170.75
https://doi.org/10.1016/j.desal.2020.114661	18.3	25.0	99.2	97.40
https://doi.org/10.1016/j.memsci.2020.118605	7.6	33.8	96.5	18.91
https://doi.org/10.1021/acsami.0c06417	46.5	32.0	93.5	10.46
https://doi.org/10.1016/j.memsci.2020.118333	15.0	11.0	92.0	11.13
https://doi.org/10.1007/s11705-020-1943-8	19.0	23.0	99.4	128.33
https://doi.org/10.1016/j.memsci.2020.118064	1.6	39.2	91.4	7.11
https://doi.org/10.1016/j.desal.2016.05.002	5.8	49.4	97.4	19.43

https://doi.org/10.1016/j.memsci.2020.118003	11.2	54.6	99.4	73.81
https://doi.org/10.1016/j.seppur.2021.119150	63.2	2.6	12.3	1.11
https://doi.org/10.1016/j.desal.2020.114811	18.5	28.9	98.4	44.46
https://doi.org/10.1002/app.50902	12.7	31.3	99.4	119.04
https://doi.org/10.1016/j.seppur.2020.117632	40.0	44.4	99.1	62.43
https://doi.org/10.1016/j.desal.2004.07.001	9.7	66.7	95.1	6.73
	11.9	62.6	96.1	9.67
https://doi.org/10.1016/j.cej.2013.03.021	11.9	39.8	95.9	14.68
https://doi.org/10.1016/j.desal.2013.11.025	9.3	21.1	96.1	20.23
https://doi.org/10.1016/j.memsci.2014.11.020	13.2	54.8	45.8	0.83
https://doi.org/10.1016/j.memsci.2014.05.047	18.2	53.6	77.4	2.05
	15.3	44.6	50.4	1.12
	6.8	13.6	99.3	123.43
https://doi.org/10.1016/j.memsci.2016.01.034	16.6	65.0	95.4	7.61
https://doi.org/10.1016/j.memsci.2016.01.028	7.0	30.0	98.0	35.00
https://doi.org/10.1016/j.memsci.2016.10.012	1.5	85.2	78.0	0.67
https://doi.org/10.1016/j.seppur.2016.09.023	13.4	51.5	98.6	34.64
	17.0	50.6	98.5	32.93
https://doi.org/10.1002/app.43511	1.6	54.6	98.2	25.22
https://doi.org/10.1039/C6RA05716K	3.1	40.9	92.3	7.68
	5.4	31.9	91.5	8.01
	6.3	36.2	38.2	1.03
	1.6	48.1	88.6	4.55
https://doi.org/10.1016/j.memsci.2016.04.049	14.2	52.0	98.4	29.45
	17.2	49.7	98.2	27.34
https://doi.org/10.1039/C5TA09252C	5.4	25.3	86.0	5.34
https://doi.org/10.1016/j.desal.2015.11.001	1.9	95.7	98.1	2.26
https://doi.org/10.1016/j.memsci.2017.05.062	6.1	29.9	95.0	14.02
	11.9	15.5	93.2	12.43
https://doi.org/10.1021/acsami.7b00159	11.5	20.0	92.1	10.14
https://doi.org/10.1007/s10924-017-1063-9	0.7	65.0	84.0	2.19
	0.9	70.0	82.0	1.67
https://doi.org/10.1016/j.desal.2017.06.006	3.2	26.5	72.1	2.63
https://doi.org/10.1016/j.memsci.2016.09.055	10.0	20.0	90.0	8.00

https://doi.org/10.1039/C7RA02374J	5.0	50.0	90.0	5.00
https://doi.org/10.1021/acsami.6b14412	6.9	28.0	93.0	10.29
https://doi.org/10.1016/j.desal.2017.10.011	0.5	93.5	99.4	11.61
https://doi.org/10.1016/j.memsci.2018.03.075	1.8	83.8	96.2	4.26
	16.2	27.4	98.3	42.71
https://doi.org/10.1016/j.cej.2018.03.116	3.1	32.0	94.0	11.33
https://doi.org/10.1039/C8RA01374H	7.7	42.2	97.3	21.41
	12.1	31.1	92.4	9.07
https://doi.org/10.1002/app.46363	7.5	15.0	90.0	8.50
https://doi.org/10.1002/app.46482	21.0	29.1	90.0	7.09
https://doi.org/10.1021/acs.iecr.8b04515	5.7	42.0	98.5	38.67
https://doi.org/10.1016/j.memsci.2018.01.048	4.4	65.9	98.6	24.36
https://doi.org/10.1007/s11705-017-1695-2	2.2	27.8	90.4	7.52
https://doi.org/10.1016/j.desal.2018.07.023	10.6	21.1	96.2	20.76
https://doi.org/10.1016/j.memsci.2018.03.004	14.5	22.0	97.0	26.00
https://doi.org/10.1021/acs.est.8b04102	10.3	16.2	98.7	64.47
https://doi.org/10.1016/j.memsci.2018.07.031	5.4	30.0	88.0	5.83
https://doi.org/10.1016/j.memsci.2017.11.057	5.9	36.8	98.5	42.13
https://doi.org/10.1038/s41598-018-23188-7	1.6	86.0	87.5	1.12
https://doi.org/10.1016/j.memsci.2018.07.044	3.0	27.0	95.0	14.60
https://doi.org/10.1016/j.memsci.2018.03.034	5.1	14.2	62.1	2.26
https://doi.org/10.1016/j.memsci.2019.02.064	9.9	44.1	98.1	29.42
https://doi.org/10.1016/j.seppur.2018.08.037	7.0	50.0	99.5	100.00
https://doi.org/10.1016/j.cej.2018.10.147	12.3	18.5	99.0	81.50
https://doi.org/10.1016/j.memsci.2018.11.019	9.8	21.6	97.0	26.13
https://doi.org/10.1039/C9EW00227H	19.3	31.3	98.9	62.45
https://doi.org/10.1016/j.arabjc.2017.12.009	1.8	32.0	95.0	13.60
	3.1	32.0	96.0	17.00
https://doi.org/10.1016/j.seppur.2018.09.034	1.7	66.2	72.2	1.22
https://doi.org/10.1039/C8TA09242G	8.6	13.0	99.0	87.00
https://doi.org/10.1016/j.memsci.2019.05.065	5.4	47.0	99.0	53.00
https://doi.org/10.1016/j.memsci.2019.117375	5.8	52.0	98.0	24.00
	15.3	35.4	95.1	13.18
https://doi.org/10.1016/j.seppur.2019.05.018	7.0	52.0	99.0	48.00

https://doi.org/10.1016/j.memsci.2020.117852	11.4	48.6	97.8	23.36
	17.2	49.6	99.3	72.00
https://doi.org/10.1016/j.memsci.2019.117717	11.0	30.0	98.6	50.00
https://doi.org/10.1021/acs.est.8b02425	2.2	10.0	75.0	3.60
https://doi.org/10.1039/C8TA08273A	9.5	50.0	97.0	16.67
https://doi.org/10.3390/polym10111253	5.5	41.0	97.5	23.60
https://doi.org/10.1016/j.memsci.2017.09.075	3.3	40.0	98.0	30.00
https://doi.org/10.1016/j.apsusc.2019.05.157	7.7	35.0	98.5	43.33
https://doi.org/10.1007/s10853-018-2847-6	3.7	85.5	99.8	80.39
https://doi.org/10.1016/j.memsci.2018.10.061	6.7	48.3	97.3	19.15
	13.2	34.0	97.6	27.50
	17.0	34.9	95.9	15.88
https://doi.org/10.1021/acs.iecr.9b00772	6.9	32.7	98.8	56.08
https://doi.org/10.1016/j.memsci.2019.01.040	12.0	17.8	97.4	31.62
https://doi.org/10.1039/C9TA02299F	14.5	35.4	99.0	64.60
https://doi.org/10.1016/j.seppur.2019.04.043	6.2	54.0	86.0	3.29
	5.0	78.0	97.0	7.33
	5.3	73.0	91.0	3.00
	4.2	47.0	87.0	4.08
	3.0	51.0	89.0	4.45
https://doi.org/10.1021/acs.iecr.9b02217	13.0	56.2	99.2	54.75
https://doi.org/10.1016/j.memsci.2019.117396	1.6	98.3	99.6	4.25
	15.2	25.9	96.1	19.00
https://doi.org/10.1016/j.memsci.2019.117386	6.0	56.3	98.8	36.42
	21.7	16.1	97.8	38.14
https://doi.org/10.1039/C5TA09252C	5.4	25.3	86.0	5.34
	7.5	18.9	96.4	22.53
https://doi.org/10.1016/j.memsci.2018.02.009	12.3	20.2	92.3	10.36
https://doi.org/10.1021/acs.est.9b06426	34.7	12.2	95.5	19.51
https://doi.org/10.1021/acsapm.9b00973	4.5	27.3	98.1	38.26
https://doi.org/10.1016/j.desal.2020.114352	4.9	40.5	97.2	21.25
	7.5	47.0	94.0	8.83
https://doi.org/10.1016/j.memsci.2019.117718	26.2	17.0	97.7	36.09
https://doi.org/10.1002/aic.16879	5.7	49.5	95.8	12.02

	15.5	18.3	94.9	16.02
https://doi.org/10.1016/j.memsci.2020.118067	5.6	23.0	92.0	9.63
	9.4	43.0	98.5	38.00
	27.5	52.5	98.5	31.67
	25.5	28.3	98.4	44.81
https://doi.org/10.1016/j.memsci.2020.118153	25.8	26.3	99.1	81.89
https://doi.org/10.1016/j.memsci.2020.118202	6.3	25.0	98.0	37.50
	25.0	20.0	95.1	16.33
https://doi.org/10.1016/j.memsci.2020.117971	20.2	22.0	97.2	27.86
	19.0	24.1	98.4	47.44
https://doi.org/10.1038/s41467-020-15771-2	12.6	15.1	97.1	29.28
	17.1	27.0	99.6	182.50
	14.9	21.3	98.8	65.58
	20.3	20.9	99.1	87.89
	26.2	9.9	98.1	47.42
	14.9	14.8	98.0	42.60
	28.3	30.7	39.7	1.15
	38.2	46.1	62.5	1.44
https://doi.org/10.1021/acs.est.0c03589	2.4	10.2	75.8	3.70
https://doi.org/10.1016/j.memsci.2019.117441	14.6	18.5	96.6	23.97
https://doi.org/10.1021/acsami.0c09510	20.2	6.3	27.0	1.28
https://doi.org/10.1016/j.desal.2020.114661	11.8	28.0	99.3	102.86
https://doi.org/10.1016/j.seppur.2020.117133	5.4	39.4	92.4	7.93
	12.9	22.0	91.6	9.29
https://doi.org/10.1016/j.memsci.2019.117472	4.0	31.4	78.4	3.17
https://doi.org/10.1021/acs.est.9b06779	9.1	23.0	95.2	16.04
https://doi.org/10.1016/j.memsci.2020.118286	1.1	45.9	92.6	7.31
https://doi.org/10.1016/j.memsci.2020.118526	9.4	33.3	98.8	55.58
	8.8	38.5	99.4	102.50
https://doi.org/10.1016/j.memsci.2020.118786	20.0	42.0	95.0	11.60
https://doi.org/10.1016/j.memsci.2019.117795	7.4	14.3	90.0	8.57
https://doi.org/10.1021/acs.iecr.5b01950	6.5	23.2	51.2	1.57
https://doi.org/10.1016/j.desal.2020.114709	18.4	16.4	70.0	2.79
https://doi.org/10.1002/ceat.201600149	10.0	35.9	99.8	320.50

	11.6	36.3	98.3	36.40
	11.2	37.9	96.5	17.74
https://doi.org/10.1039/C7RA00621G	7.7	15.8	92.1	10.64
https://doi.org/10.1016/j.seppur.2020.116528	0.8	61.1	92.3	5.07
	5.1	74.2	96.6	7.67
https://doi.org/10.1016/j.desal.2020.114340	9.2	35.5	99.1	70.96
https://doi.org/10.1016/j.memsci.2020.118064	3.6	12.7	79.9	4.33
https://doi.org/10.1016/j.jhazmat.2017.09.016	9.6	49.7	94.9	9.91
https://doi.org/10.1021/acs.nanolett.9b00060	12.4	47.7	94.7	9.89
	1.6	73.1	53.7	0.58
	8.2	59.0	94.0	6.84
https://doi.org/10.1007/s12221-010-1041-5	13.3	24.7	92.5	9.97
https://doi.org/10.1039/C5RA02560E	5.9	26.0	97.6	31.42
	8.5	27.4	98.8	59.29
https://doi.org/10.1016/j.memsci.2014.11.011	13.3	31.0	94.8	13.16
https://doi.org/10.1016/j.memsci.2015.04.072	10.4	58.7	99.7	146.55
https://doi.org/10.1016/j.memsci.2016.07.038	4.1	40.3	87.2	4.65
	8.8	45.3	89.0	4.99
	17.2	20.1	82.8	4.64
https://doi.org/10.1016/j.memsci.2015.12.064	7.9	70.2	97.5	12.11
https://doi.org/10.1021/acs.iecr.6b00409	13.8	63.5	96.2	9.69
https://doi.org/10.1002/app.45422	4.7	45.7	93.1	7.92
https://doi.org/10.1002/app.45301	13.0	23.1	78.4	3.56
	3.0	71.9	90.2	2.87
https://doi.org/10.1016/j.memsci.2017.04.043	2.0	35.5	95.6	14.70
https://doi.org/10.1126/science.aar6308	19.3	66.6	99.5	68.12
https://doi.org/10.1039/C8TA00342D	4.7	38.7	98.9	56.20
	13.1	41.5	99.1	67.54
https://doi.org/10.1007/978-3-319-05041-6_3	4.7	70.7	80.0	1.46
https://doi.org/10.1021/acsami.8b19121	10.5	54.2	96.1	11.78
https://doi.org/10.1016/j.memsci.2019.117244	7.8	58.0	98.7	31.35
https://doi.org/10.1016/j.seppur.2020.117632	40.4	44.6	99.1	62.19
https://doi.org/10.1016/j.memsci.2020.118557	25.3	52.7	99.9	397.49
https://doi.org/10.1016/j.memsci.2021.119154	4.9	49.0	92.2	6.54

	19.5	17.6	98.9	74.91
https://doi.org/10.1021/acsestengg.0c00213	10.3	8.9	98.7	71.67
	7.1	16.6	99.1	98.00
	15.0	48.2	98.9	47.94
	5.5	62.6	99.1	41.10

Supplementary Table 2. The I-O list of NF membrane unit.

Category	Item	Amount	Unit	Cost (CNY)	Cost (USD)
Energy	Electricity	2.361822356	kWh	1.4193	0.1987
Chemical agent	citric acid	0.003740177	kg	0.0579	0.0081
	sodium tripolyphosphate	0.003065734	kg	0.0227	0.0032
	EDTA-4Na	0.001226271	kg	0.0411	0.0057
	liquid alkaline (50%)	0.003249603	kg	0.0084	0.0012
	scale inhibitor	0.00738831	kg	1.6025	0.2244
Wastewater input	NaCl	31	kg		0
	Na_2SO_4	22.3	kg		0
	water	1000	kg		0
Wastewater output (permeate liquid)	Na_2SO_4	0.95239865	kg		0
	NaCl	19.6777595	kg		0
	water	700	L		0
Wastewater output (concentrated liquid)	NaCl	11.3222405	kg		0
	Na_2SO_4	21.34760135	kg		0
	water	300	L		0

Supplementary Table 3. The I-O list of NaCl crystallization unit.

Category	Item	Amount	Unit	Cost (CNY)	Cost (USD)
Energy	Electricity	20.03743512	kWh	12.02312314	1.683237239
Chemical agent	citric acid	0.000613073	kg	0.009930994	0.001390339
	sodium tripolyphosphate	0.001532518	kg	0.011586159	0.001622062
	EDTA-4Na	0.000613073	kg	0.008275828	0.001158616
	steam	13.60913959	kg	2.313921551	0.323949017
	hydrochloric acid (30%)	0.04597686	kg	0.034758478	0.004866187
	liquid alkaline (50%)	0.031604395	kg	0.081103116	0.011354436
	defoaming agent	0.002942553	kg	0.142344244	0.019928194
	scale inhibitor	0.001348629	kg	0.021517153	0.003012401
Wastewater input	Na_2SO_4	0.95239865	kg		
	NaCl	19.6777595	kg		
	water	350	L		
Output	NaCl	18.9006195	kg	-13.230433	-1.8522607
	hazardous waste	1.72953865	kg	3.4590773	0.484270822
	water(emission to air)	350	L		

Supplementary Table 4. The I-O list of A-NF membrane unit.

Category	Item	Amount	Unit	Cost (CNY)	Cost (USD)
Energy	Electricity	2.361822356	kWh	1.4193	0.1987
Chemical agent	citric acid	0.001345301	kg	0.0208	0.0029
	sodium tripolyphosphate	0.001102711	kg	0.0082	0.0011
	EDTA-4Na	0.000441076	kg	0.0148	0.0021
	liquid alkaline (50%)	0.001168846	kg	0.0030	0.0004
	scale inhibitor	0.002657494	kg	0.5764	0.0807
Wastewater input	NaCl	31	kg		
	Na_2SO_4	22.3	kg		
	water	1000	kg		
Wastewater output (permeate liquid)	Na_2SO_4	0.49	kg		
	NaCl	23.142	kg		
	water	700	L		
Wastewater output (concentrated liquid)	NaCl	7.858	kg		
	Na_2SO_4	21.81	kg		
	water	300	L		

Supplementary Table 5. The I-O list of NaCl crystallization unit (A-NF).

Category	Item	Amount	Unit	Cost (CNY)	Cost (USD)
Energy	Electricity	20.03743512	kWh	12.02312314	1.683237239
Chemical agent	citric acid	0.000613073	kg	0.009930994	0.001390339
	sodium tripolyphosphate	0.001532518	kg	0.011586159	0.001622062
	EDTA-4Na	0.000613073	kg	0.008275828	0.001158616
	steam	13.60913959	kg	2.313921551	0.323949017
	hydrochloric acid (30%)	0.04597686	kg	0.034758478	0.004866187
	liquid alkaline (50%)	0.031604395	kg	0.081103116	0.011354436
	defoaming agent	0.002942553	kg	0.142344244	0.019928194
	scale inhibitor	0.001348629	kg	0.021517153	0.003012401
Wastewater input	Na_2SO_4	0.49	kg		
	NaCl	23.142	kg		
	water	350	L		
Output	NaCl	23.064405	kg	-16.1450835	-2.26031169
	hazardous waste	0.56766395	kg	1.1353279	0.158945906
	Water (emission to air)	350	L		
