# Supplementary material for: Suppressing Polyamide Crystallization Unlocks Ultrapermeable and Highly Selective Nanofiltration Membranes
Source: Nat Commun. 2026 Sep 3;17:10481. doi: 10.1038/s41467-026-76628-8 (PMC13635223; doi:10.1038/s41467-026-76628-8)
Supplement: Supplementary file 2 [file 41467_2026_76628_MOESM2_ESM.pdf]

nature portfolio

Peer Review File

Suppressing Polyamide Crystallization Unlocks Ultrapermeable and Highly Selective Nanofiltration Membranes

Corresponding Author: Professor Menachem Elimelech

This file contains all reviewer reports in order by version, followed by all author rebuttals in order by version.

Version 0:

Reviewer comments:

Reviewer #1

(Remarks to the Author)
This manuscript presents a novel strategy to regulate the crystalline structure of the selective layer in nanofiltration membranes via ultra-low-temperature liquid–liquid interfacial polymerization at -20°C. The resulting selective layer exhibits an amorphous structure while maintaining excellent selectivity and permeability. The fabricated membrane demonstrates outstanding overall performance in practical high-salinity wastewater treatment. From the perspective of intrinsic polymer crystallinity, this work provides a new strategy for the design of nanofiltration membranes. It provides a new perspective for structural design in this field and is therefore recommended for publication in Nature Communications. However, several issues need to be addressed before acceptance. The specific comments are as follows:

1. Besides microcrystallinity suppression, could other factors also contribute to the observed improvement in NF performance?
2. What is the key difference between this method and existing ultra-low-temperature interfacial polymerization approaches, and what are its main advantages?
3. The method used to characterize the polyamide density appears interesting and effective, but the current description is too brief. Please provide a more detailed description of this method.
4. A-NF has demonstrated excellent anti-fouling performance in both laboratory-scale studies and actual water treatment applications. What is the main mechanism responsible for the improved antifouling performance?
5. In the practical wastewater evaluation, the salt selectivity of A-NF shows a noticeable decline compared to laboratory conditions. What are the reasons for this behavior?
6. Residual TOC is present in the influent (Fig. 4b). How effective is A-NF at removing residual TOC in practical water treatment applications?

Reviewer #2

(Remarks to the Author)
Comment:
This study introduces a dual strategy of co-solvent and low-temperature interfacial polymerization to fabricate NF membranes. By precisely modulating the formation and packing kinetics, amorphous polyamide chains were consequently achieved, leading to enhancement in both water permeability and ion selectivity. While the use of a methanol-water aqueous phase facilitates fine control over the internal architecture of the selective layer, critical aspects, including material cost, process reproducibility, energy consumption, and manufacturability at industrial scale, remain to be systematically evaluated. Addressing these challenges is essential to advance the strategy toward practical implementation and rigorous peer-reviewed publication. Some other issues also are listed below, which should be carefully addressed.
1. What was reaction temperature used for the preparation of SC-NF?
2. The results show that the crosslinking degree of A-NF and C-NF is similar, and the thickness of the A-NF membrane has significantly decreased. Please further analyze why the water flux of the membrane has suddenly increased under these conditions? What is the core mechanism driving this increase in water flux? Does this mean that the traditional conclusion

that "crosslinking degree determines permeability" is invalid in this case? Please further verify the core mechanism of the relationship between the dominant structure and performance.
3. For polymers with linear configuration, crystallization could be triggered upon applying external pressure for prolonged time. While for polyamide, will this also occur after long-term filtration?
4. Since the dual strategy could also reduce the polyamide thickness, which was beneficial for water passing through, then which effect would dominate the great enhancement in flux, thickness reduction or amorphous structure? How to quantify the contribution of each factor?
5. Although the high permeability of A-NF to Ca^{2+} alleviates membrane fouling contamination, it will result in a large amount of Ca^{2+} migrating to the product water side. In a typical ZLD process, should the issue of new scaling problems caused by this in the subsequent evaporation crystallization unit be considered, as well as the potential problems it brings to the system stability and product purity?
6. Is the low-temperature interface polymerization method for membranes applicable to other monomer systems? Does the applicability of this strategy need to be customized and designed based on the specific chemical properties of different monomer structure systems?
7. The basal membrane was first immersed in the aqueous phase at -20°C, and then transferred to the oil phase at room temperature for interface polymerization. This operation process could introduce a significant temperature gradient. Does the author quantitatively evaluate the impact of this temperature transition from the following three aspects: (1) the concentration distribution process of PIP monomers in the aqueous phase boundary layer; (2) the local adsorption conformation and spatial distribution of TMC at the interface; (3) the spatial uniformity of the crosslinking density of the polyamide network? It is recommended to consider the interference of this thermal-dynamic coupling process on the stability of the interface, which affects the controllability and batch reproducibility of the amorphous membrane structure.
8. The claim of a > 400% increase in water permeability should be accompanied by a comparative analysis with existing literature to contextualize the performance enhancement.
9. The manuscript often refers to "ultra-smooth" surfaces and "defect-free" structures, but more detailed quantitative data should be provided to substantiate these claims. How are ultra-smooth surfaces defined internationally?
10. This work focuses on water permeability and salt rejection, but long-term stability and fouling resistance are only briefly addressed. Furthermore, 800 min long-term operation time is too short and not convincing.
11. In the MD simulations, how did the authors determine the degree of cross-linking? Do the experimental crosslinking data align with the simulation results? Please provide details.
12. In addition to the MD simulations of interfacial polymerization, the authors should conduct pressure-driven desalination simulations to better illustrate the desalination mechanism.
13. The hypothesis about the role of methanol in enhancing the diffusion of PIP should be supported by experimental verification.
14. At the end of this manuscript, the authors conducted a LCA analysis on industrial wastewater treatment, which was totally meaningless to me. Instead, the authors should focus more on assessing the energy consumption required to maintain the low temperature during large-scale production and its corresponding carbon footprint, comparing with traditional methods to further verify the scalability and actual energy cost of this technology. Moreover, the unit price of the A-NF membrane sheet prepared at -20°C should been included for comparison with the traditional NF membranes?

Reviewer #3

(Remarks to the Author)
This work presents a novel approach to membrane fabrication. Specifically, the phase inversion process is conducted a very low temperature of -20°C by doping the water phase with methanol, thereby avoiding the water freeze at normal freeze point. The resultant membrane demonstrates superior processing performance with increased flux, rejection, and fouling characteristics. These are attributed to the fact that the low temperature in the bath helps to slow down the interfacial polymerisation process and results in a thinner and smoother membrane, while maintaining NF pore size. This is a very nice piece of work and is definitely of interest to readers, I commend the authors on this front.

There are some issues with the work. Firstly, there are no stated aims at the front of the paper as would be expected. What is provided is a set of conclusive remarks based on the results which have not been presented at this point. This needs to be rationalised. Secondly, there is something very strange going on in this paper in that two experiments are reported in the text of the paper, yet the figures in the supplementary data file report on three. This has to be sorted out as confusion is introduced throughout the paper as a result. Thirdly, while I understand that authors like to sell their work, there is a lot of sensationalism in the text as provided that is not warranted. There are many areas to the text where the authors infer that the membrane is industrially significant or relevant, when the reality of the situation is that the membrane has not been tested under industrial conditions or for appropriate timescales. This language needs to be toned down, and the claims made in a more appropriate fashion. Afterall, this membrane currently only exists at the size of a postage stamp…

Finaly, this work leaves me with one major question. The authors have doped the feed solution with methanol to operate at -20°C. Why not -10°C or -30°C? If the work were to be repeated at these temperatures would a better membrane result? There has been a massive amount of time and effort placed on characterisation of the single experiment membrane when I feel a range of experimental temperatures and less characterisation may have been more beneficial. I guess this could be a subject for future work (which is not mentioned at all).

My recommendation is publication following corrections.

Specific comments:

Title: I think the word ‘membranes’ is missing from the end of the title, i.e. should the title be ‘Suppressing Polyamide Crystallization Unlocks Ultrapermeable and Highly Selective Nanofiltration Membranes’?
Front page. Please check affiliation d, it is posted as c (c is listed twice).
Abstract: Line 38 has a random (167) in the text line. Please remove.
Line 47. Should the section title be ‘Introduction’ rather than ‘Main’?
Line 90,93,99. Please avoid the use of personal pro-nouns, i.e. I, we…etc. Scientific text should be written in the third person. I have also noticed this issue throughout the rest of the text. Please address all instances.

Lines 90 to 104: All of the text in this section represents conclusive remarks relating to the work presented in this paper before the results have actually been presented. Please rework this section of the text to deliver the aims of the paper and not the conclusions from the work. The aims should reflect the gap in current scientific knowledge and how the work will address those gaps.

Line 107: What is ‘PIP’ the acronym has been introduced with no explanation.

Line 166 to 169: The surface roughness of the membranes is being discussed and figure 2c clearly indicates that the relative roughness of the resulting membranes is 21.1 nm and 2.2 nm respectively. What then is supplementary figure 3 showing with an Ra = 12.9 nm? The caption gives no information at all in relation to this phenomenon and the value is half way between the 25°C and -20°C data. This is confusing, please explain what is happening.

Line 173 to 177. In this section of text you state that the membrane thickness is reduced from 65nm to 32nm as a result of the process. I assume that this is the fabrication of the membrane at 25°C and -20°C (note I have to assume). Then when I look at supplementary figure 5 there are three sets of plots related to this data set. These are labelled C-NF, SC-NF, and A-NF. This is very confusing as the main paper is indicating there are two sets of data, the supplementary information is telling me there are three. Similarly, the legend descriptors are not explained anywhere in the paper, I have found A-NF on line 192 but this acronym is not introduced. The other two are not explained anywhere. Lines 429-430 infer that the A-NF is the amorphous membrane and C-NF is the crystalline membrane – is this the case as I don’t like to guess? I see no mention of SC-NF anywhere. Please rationalise this situation by ensuring that the methods are fully explained and the figure captions are complete, i.e. they explain the figure in full detail. Why is there a third plot when only two experiments are reported?

Line 260 introduces figure 3b. There appears to be a great many grey diamonds on this plot that make no logical sense and are not reported in the legend or caption. What are these data points? Also, at this point supplementary table 1 is introduced. Firstly, why not give the column titles proper text, i.e. specific flux (Lm-2h-1bar-1) rather than A? Secondly, this table is very dubious. What I mean by this is that rejection of salts will depend on applied pressure, concentration, and pH to name but a few factors. Therefore, to make a fair comparison, all of the data sets reported would need to be experiments conducted at the same experimental conditions. Thus, the claim that the membrane has higher selectivity is not substantiated by this table. So, I have to ask myself what is the point of this table? In addition, there are some reports in the table where the specific flux is significantly higher than that observed in this study. I would have to question if these studies are actually characterising NF membranes as fluxes this high would suggest that they are indeed UF membranes. Again, what is the purpose of this table as it does not validate the membrane characteristics obtained in this work?

Lines 266 to 272: Here you indicate that the long-term performance of NF membranes is important. I totally agree. How is 120 hours long term performance? This is 5 days of data, i.e. a working week. This is not long term at all. Please amend the text accordingly to remove the claim. Supplementary figure 14 is introduced at this point. Can you explain what the grey shadow is on the water flux data set? Is this an error bar? If it is just image and not representative of actual data then please remove as it creates confusion. In addition, most commercial NF membranes operate up to around 30-40 bar pressure, and your text indicates ‘wide range of operating pressures’. This is misleading as the work is limited to the low operating pressure range as detailed in the caption on the figure. Out of interest, does this membrane offer superiority in the high-pressure ranges as well? Also of note, your figure shows a random -20°C, surely this is not the operating temperature of the experiment. Please remove and add actual conditions to the caption.
I have now seen supplementary figure 16 (SF16). Now I am confused as this figure and SF14 are surely showing the same data set with SF16 showing an increased data set. Why are both needed?

Lines 311 to 322. The inference is made here that the new membrane demonstrates better anti-fouling properties. From the data provided, I agree with these statements. However, is it fair to infer from these experiments that the membrane ‘is advantageous for applications involving industrial wastewaters’ when the conditions of these experiments are far from those that would be typical of seawater desalination or industrial effluents? I feel the language used needs to be reflective of what has been done and not sensationalised without appropriate data to back up these statements.

Again, as with lines 329 to 336, 1500 minutes is one day of operation. Significant fouling occurs over time, and this experiment is not based over a long time period. So, while I agree that the demonstrated effect of less fouling is there, the text needs to be contextualised in light of the demonstrated operation and not inferred that there is superior industrial performance when this has not been demonstrated.

Methods: Where was the ultrapure water obtained?
Line 459: Why is the symbol ‘S’ used for membrane area rather than ‘A’ as would be expected? Why then is specific flux termed ‘A’ when papers would normally use ‘J’ for flux and ‘P’ for permeance? Why add confusion by swapping from normal nomenclature.

Line 463: Again, why is symbol ‘Co’ used in place of the typically expected ‘Cp’ the concentration in the permeate?

Line 542: Reference 20 has an anomalous ‘1’, please remove.

Version 1:

Reviewer comments:

Reviewer #1

(Remarks to the Author)
Questions addressed. Can be accepted now.

Reviewer #2

(Remarks to the Author)
better

Reviewer #3

(Remarks to the Author)
Dear Editor and authors
I have now taken the time to read through the responses to the original review questions that I made and am happy that the reviewers have taken on board all of the points raised. I am happy for the paper to progress to publication. I would like to thank the authors for the rigorous responses which made review far easier.

Open Access This Peer Review File is licensed under a Creative Commons Attribution 4.0 International License, which permits use, sharing, adaptation, distribution and reproduction in any medium or format, as long as you give appropriate credit to the original author(s) and the source, provide a link to the Creative Commons license, and indicate if changes were made.
In cases where reviewers are anonymous, credit should be given to 'Anonymous Referee' and the source.
The images or other third party material in this Peer Review File are included in the article’s Creative Commons license, unless indicated otherwise in a credit line to the material. If material is not included in the article’s Creative Commons license and your intended use is not permitted by statutory regulation or exceeds the permitted use, you will need to obtain permission directly from the copyright holder.
To view a copy of this license, visit https://creativecommons.org/licenses/by/4.0/

Reviewer #1:

Reviewer: This manuscript presents a novel strategy to regulate the crystalline structure of the selective layer in nanofiltration membranes via ultra-low-temperature liquid–liquid interfacial polymerization at -20°C. The resulting selective layer exhibits an amorphous structure while maintaining excellent selectivity and permeability. The fabricated membrane demonstrates outstanding overall performance in practical high-salinity wastewater treatment. From the perspective of intrinsic polymer crystallinity, this work provides a new strategy for the design of nanofiltration membranes. It provides a new perspective for structural design in this field and is therefore recommended for publication in Nature Communications. However, several issues need to be addressed before acceptance. The specific comments are as follows:

Our Response: We sincerely thank the reviewer for the highly positive evaluation of our work and for recognizing the novelty and significance of our strategy for regulating the crystalline structure of nanofiltration selective layers via ultra-low-temperature interfacial polymerization. We have carefully considered all of the reviewer's comments and suggestions and have revised the manuscript accordingly. We believe that these revisions have significantly improved the clarity, rigor, and overall quality of the manuscript. We sincerely appreciate the reviewer's constructive feedback and valuable assistance in improving this work.

1- Besides microcrystallinity suppression, could other factors also contribute to the observed improvement in NF performance?

Our Response: We thank the reviewer for this insightful comment. In addition to the suppression of microcrystalline domains, other structural and physicochemical factors may also contribute to the enhanced nanofiltration performance. Specifically, the reduced thickness of the selective layer shortens the transport pathway for water molecules, thereby facilitating water permeation. Meanwhile, the enhanced negative surface charge can strengthen Donnan exclusion toward multivalent anions, contributing to improved selectivity. We have added further discussion in the revised manuscript as follows:

Line 185: Furthermore, the ultralow-temperature system successfully reduced the thickness of the polyamide layer from 65 nm to 32 nm **(Fig. 2d and Supplementary Fig. 7)**. The reduced thickness of the selective layer shortens the transport pathway for water molecules, thereby facilitating water permeation.

Line 242: The increased carboxyl group content endows A-NF with a more negative surface potential **(Supplementary Fig. 10)**, which is beneficial for the rejection of divalent anions by strengthening Donnan exclusion effects and thereby contributes to enhanced membrane selectivity.

2- What is the key difference between this method and existing ultra-low-temperature interfacial polymerization approaches, and what are its main advantages?

Our Response: We thank the reviewer for this important question. In previously reported ultra-low-temperature interfacial polymerization approaches, lowering the aqueous-phase temperature below 0 °C generally leads to freezing of the aqueous solution. Under such conditions, monomer diffusion within the solid phase is significantly hindered, and the resulting solid–liquid interfacial polymerization process shows limited compatibility with existing industrial membrane fabrication lines.

In our work, the introduction of methanol enables the aqueous phase to remain in the liquid state even at −20 °C, allowing interfacial polymerization to proceed under a fully liquid–liquid environment. This provides a more controllable polymerization process and is expected to offer improved scalability and industrial applicability, since simply lowering the aqueous-phase temperature is straightforward to implement in current membrane manufacturing processes.

3- The method used to characterize the polyamide density appears interesting and effective, but the current description is too brief. Please provide a more detailed description of this method.

Our Response: We thank the reviewer for recognizing the interest and potential effectiveness of our polyamide density characterization method. In this method, the density of the polyamide is indirectly determined by suspending the polyamide fragments in salt solutions. When the polyamide remains stably suspended, the density of the corresponding salt solution is considered approximately equal to that of the polyamide. To improve clarity and reproducibility, we have substantially expanded the methodological description in the revised manuscript. We believe this method may also serve as a generally applicable approach for determining the density of polyamide materials. The revised methodology is provided as follows (Supporting information: Polyamide density measurement method).

The nanofiltration membrane was immersed in N,N dimethylformamide to dissolve the polysulfone substrate and obtain the separated polyamide layer. The polyamide layer was then transferred to ultrapure water for complete washing. The polyamide layer was then transferred to a saturated aqueous solution of $NaNO_3$. At this point, the solution density was greater than the polyamide density, causing it to float on the surface. Ultrapure water was added to the solution to dilute it and reduce its density, and the floating state of the polyamide was observed. As the solution density decreased, the polyamide gradually transitioned from a floating state to a suspended state, at which point the density of the polyamide became equal to that of the solution. The density of the polyamide could then be obtained by measuring the density of the solution using an analytical balance.

4- A-NF has demonstrated excellent anti-fouling performance in both laboratory-scale studies and actual water treatment applications. What is the main mechanism responsible for the improved antifouling performance?

Our Response: We thank the reviewer for this insightful question. We believe that the improved antifouling performance of A-NF originates from different mechanisms for organic fouling and inorganic scaling.

For organic fouling, the enhanced resistance is mainly attributed to the smoother membrane surface of A-NF, which reduces the adhesion tendency of foulants under cross-flow filtration conditions. The QCM-D results further support this interpretation, showing weaker foulant adsorption and interaction on the A-NF surface. Moreover, the enhanced negative surface charge of A-NF promotes stronger electrostatic repulsion toward negatively charged humic acid molecules, further suppressing their adsorption on the membrane surface.

For inorganic scaling, the mitigation effect is primarily related to the high permeation rate of calcium ions through the membrane, which suppresses the local accumulation of calcium species near the membrane surface and thereby reduces the likelihood of gypsum scale formation. This mechanism is also consistent with numerous previous studies reported in the literature.

To clarify these points, we have added further discussion regarding the enhanced antifouling and anti-scaling performance in the revised manuscript as follows:

Line 352: This enhanced scaling resistance is mainly attributed to the higher calcium ion permeation of A-NF, which mitigates local ion accumulation and suppresses surface supersaturation, as well as to its smoother surface morphology, which reduces potential nucleation sites for gypsum crystallization

Line 359: This antifouling advantage is expected to be further enhanced under practical cross-flow filtration conditions, where hydrodynamic shear can effectively suppress foulant accumulation. In addition, the enhanced negative surface charge of A-NF strengthens electrostatic repulsion toward negatively charged humic acid molecules, further contributing to its reduced foulant adsorption.

5- In the practical wastewater evaluation, the salt selectivity of A-NF shows a noticeable decline compared to laboratory conditions. What are the reasons for this behavior?

Our Response: We thank the reviewer for this important question. Compared with the laboratory-scale tests using simplified feed solutions, the practical wastewater contains much more complex components, including dissolved organic matter and various coexisting ions. Previous studies have demonstrated that the presence of organic matter in water can interfere with ion transport behavior and reduce the salt selectivity of nanofiltration membranes [38]. Therefore, the decline in salt selectivity observed during practical wastewater treatment is mainly attributed to the increased complexity of the feed composition under real operating conditions. To clarify this point, we have added additional discussion in the revised manuscript as follows:

Line 390: The slightly reduced selectivity compared with laboratory-scale measurements can be attributed to higher concentration polarization and the competitive adsorption of feed constituents under continuous crossflow operation, which partially screens membrane surface charge and weakens Donnan exclusion during Cl^-/ SO_4^{2-} separation [38].

[38] Liu, W. et al. Salting-Out Effect-Enhanced Organics Adsorption Diminishes the Chloride/Sulfate Separation Selectivity of Nanofiltration Membranes For High Salinity Wastewater. Environmental Science & Technology 60, 5867-5876 (2026).

6- Residual TOC is present in the influent (Fig. 4b). How effective is A-NF at removing residual TOC in practical water treatment applications?

Our Response: We thank the reviewer for this valuable question. We have supplemented the manuscript with a comparison of the TOC removal performance of A-NF and NF270 during practical wastewater treatment. The results show that both A-NF and NF270 can effectively reject residual TOC in the real wastewater feed, while A-NF exhibits slightly higher TOC removal efficiency than NF270. The additional data and corresponding discussion have now been incorporated into the revised manuscript.

Line 393: Meanwhile, A-NF achieved a TOC removal efficiency of 94.1% (**Supplementary Fig. 30**), slightly higher than that of NF270 (92.5%), indicating that its superior ion selectivity was maintained without compromising organic matter removal performance.

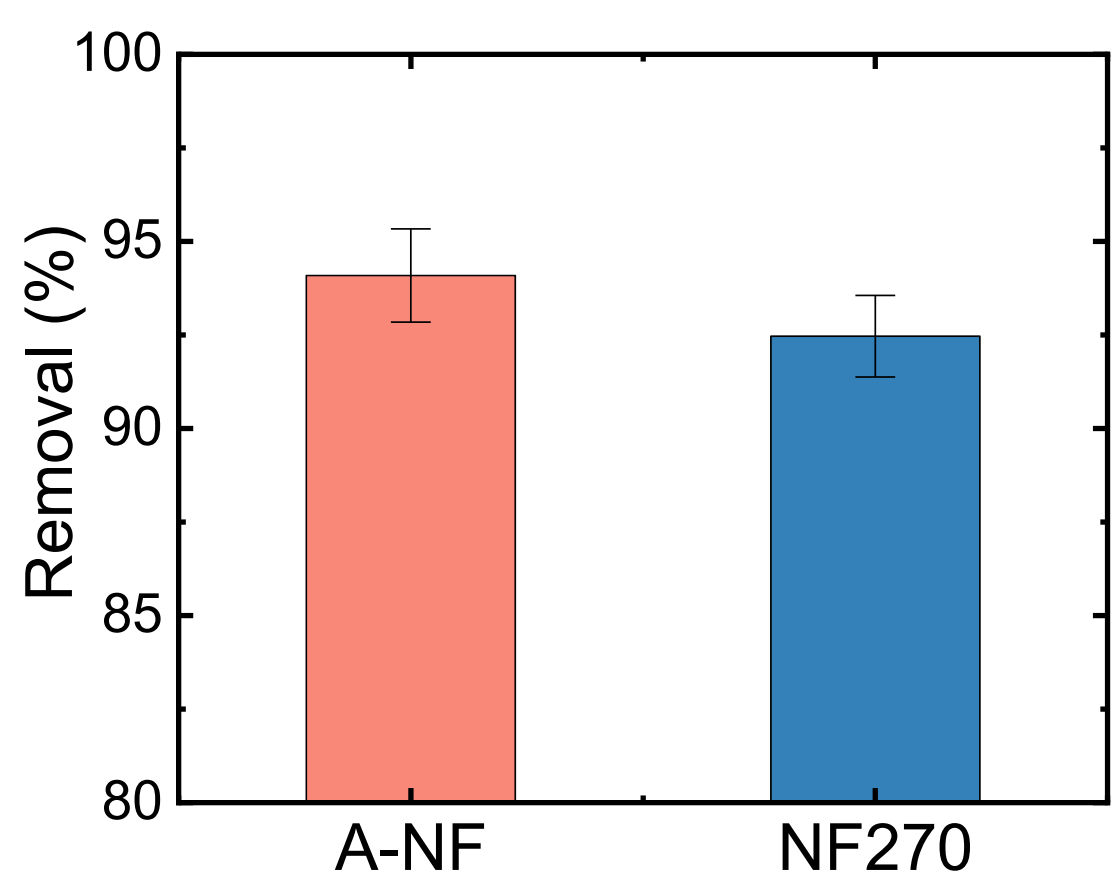

Supplementary Fig. 30: Rejection of Cl- by A-NF, C-NF and NF270 in actual wastewater

Reviewer #2:

Reviewer: This study introduces a dual strategy of co-solvent and low-temperature interfacial polymerization to fabricate NF membranes. By precisely modulating the formation and packing kinetics, amorphous polyamide chains were consequently achieved, leading to enhancement in both water permeability and ion selectivity. While the use of a methanol-water aqueous phase facilitates fine control over the internal architecture of the selective layer, critical aspects, including material cost, process reproducibility, energy consumption, and manufacturability at industrial scale, remain to be systematically evaluated. Addressing these challenges is essential to advance the strategy toward practical implementation and rigorous peer-reviewed publication. Some other issues also are listed below, which should be carefully addressed.

Our Response: We sincerely thank the reviewer for the positive assessment of our work and for raising these important concerns regarding the practical feasibility and scalability of the proposed strategy. In response, we have conducted several additional studies and revised the manuscript accordingly:

[1] We performed additional experiments to directly verify that methanol promotes PIP diffusion at the aqueous-organic interface, providing further mechanistic insight into the co-solvent effect.
[2] We extended the proposed strategy to both PEI-TMC and MPD-TMC systems, demonstrating its applicability beyond the PIP-TMC chemistry.
[3] We conducted a preliminary estimation of the energy consumption and associated carbon emissions of the low-temperature fabrication process to evaluate its potential environmental impact.

We appreciate the reviewer's suggestions regarding additional MD simulations. However, the MD simulations in this work were specifically designed to investigate monomer diffusion and mass transfer behaviors during interfacial polymerization rather than to construct polyamide network structures or quantify cross-linking degrees. Furthermore, the central finding of this study is the regulation of crystalline regions within the polyamide layer, whereas current membrane simulation frameworks are predominantly based on amorphous cross-linked polyamide models and rely mainly on cross-linking degree as the structural descriptor. Due to the lack of established methodologies for accurately constructing and characterizing crystalline polyamide structures, additional simulations such as cross-linking analysis or pressure-driven desalination simulations would be unable to reliably represent the structural features of the membranes studied here and might lead to misleading conclusions. We therefore believe that such simulations are beyond the scope of the present work, although developing simulation models capable of capturing crystalline polyamide structures represents an important direction for future research.、

We would like to emphasize that our findings do not challenge the widely accepted view that crosslinking degree is a key structural parameter governing nanofiltration membrane performance. Rather, we believe that crosslinking remains an important

factor influencing the permeability–selectivity relationship of polyamide membranes. However, the commonly used XPS-derived O/N ratio may not always accurately reflect the intrinsic crosslinking degree of the entire selective layer, particularly for structurally heterogeneous membranes, because XPS probes only the outermost surface region.

From this perspective, our work does not seek to replace the conventional crosslinking-based understanding, but instead introduces an additional structural dimension from the viewpoint of polymer physics and chemistry. Specifically, our results suggest that the crystallinity and internal structural organization of the polyamide selective layer can also play a critical role in determining membrane transport behavior. Therefore, membrane performance should be understood as being governed not only by chemical crosslinking characteristics but also by the physical packing state of polymer chains. We believe that this finding complements the existing framework and provides a new perspective for understanding the structure–performance relationship of polyamide nanofiltration membranes.

We hope that the additional experiments, analyses, and discussions presented in the revised manuscript adequately address the reviewer's concerns regarding the mechanistic understanding, general applicability, and practical feasibility of the proposed strategy. We sincerely appreciate the reviewer's constructive comments and valuable suggestions, which have helped us strengthen the scientific rigor of the study and improve the overall quality and clarity of the manuscript.

1-What was reaction temperature used for the preparation of SC-NF?

Our Response:. Thank you for pointing this out. We apologize for the confusion caused by the naming of the membranes. In our study, SC-NF refers to the membrane prepared at an interfacial polymerization temperature of 0 °C. Specifically, the preparation temperatures for the three membranes are as follows: C-NF at 25 °C, SC-NF at 0 °C, and A-NF at −20 °C.

To avoid further misunderstanding, we have revised the manuscript to clearly define the membrane names and their corresponding preparation temperatures. The revised content is as follows:

Line 169: A series of characterization methods were used to evaluate the effect of ultra-low temperature interfacial polymerization on the structure and morphology of NF membranes. To systematically investigate the influence of interfacial polymerization temperature, three representative temperatures were selected, yielding membranes denoted as C-NF, SC-NF, and A-NF, prepared at 25 °C, 0 °C, and −20 °C, respectively.

2-The results show that the crosslinking degree of A-NF and C-NF is similar, and the thickness of the A-NF membrane has significantly decreased. Please further analyze why the water flux of the membrane has suddenly increased under these conditions? What is

the core mechanism driving this increase in water flux? Does this mean that the traditional conclusion that "crosslinking degree determines permeability" is invalid in this case? Please further verify the core mechanism of the relationship between the dominant structure and performance.

Our Response:. Thank you for this insightful question. The reduction in membrane thickness indeed contributes directly to the enhancement of water permeability, as water permeability is inversely proportional to the selective-layer thickness. However, the increase in permeability cannot be explained solely by the decrease in thickness. To decouple the effect of membrane thickness, we calculated the thickness-normalized water permeability by multiplying the measured permeability by the selective-layer thickness. As shown in Supplementary Fig. 15, the normalized water permeability of A-NF still increases by approximately twofold compared with C-NF. This result suggests that additional structural factors are responsible for the enhanced transport properties.

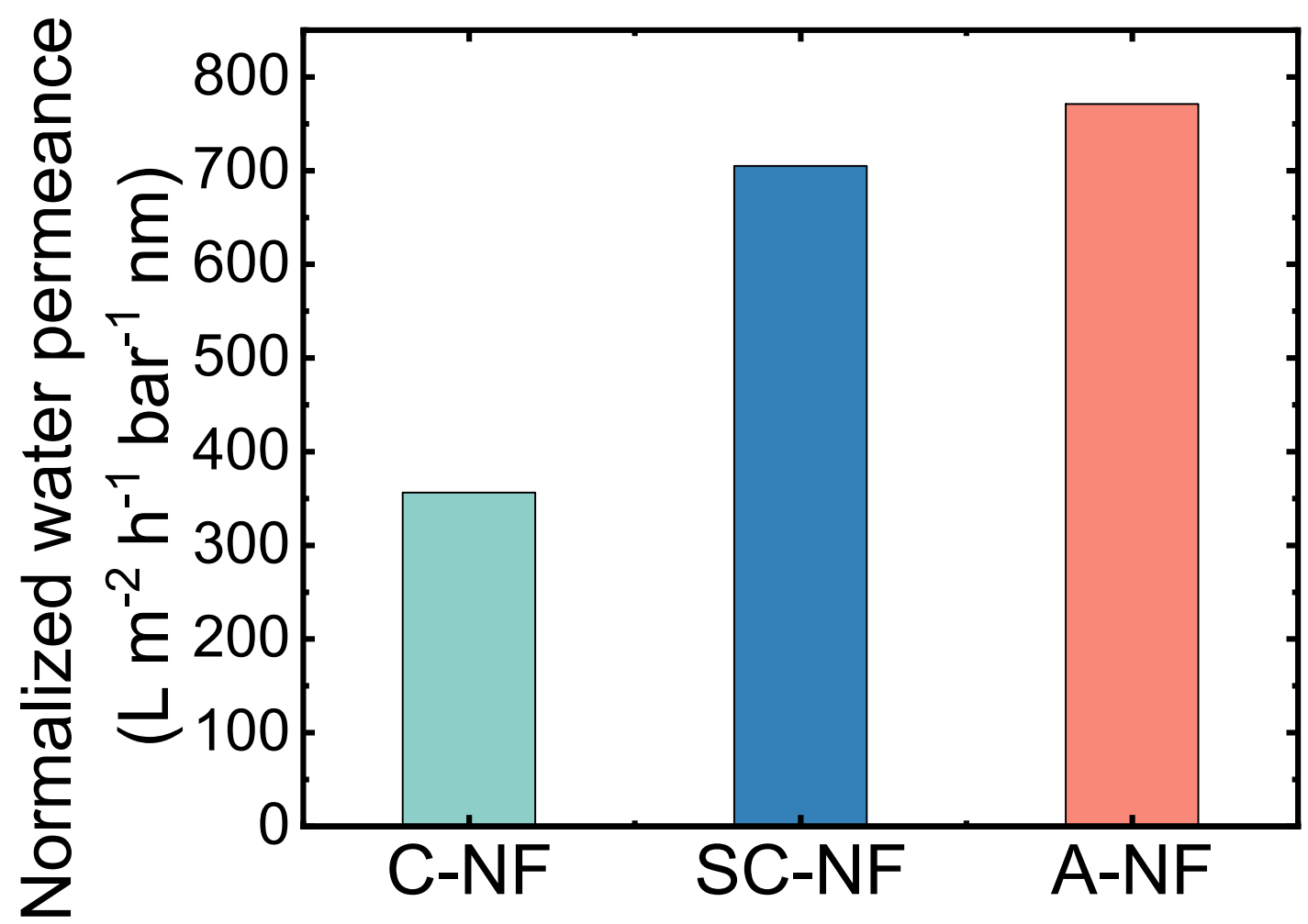

Supplementary Fig. 15: Thickness-normalized water permeance of C-NF, SC-NF, and A-NF. The water permeance was normalized by multiplying the measured permeance by the corresponding selective-layer thickness.

Regarding the relationship between crosslinking degree and permeability, we do not intend to challenge the conventional understanding that crosslinking degree influences membrane transport performance. We agree that crosslinking is an important structural parameter governing permeability–selectivity trade-offs. However, we have concerns regarding the commonly used method for estimating crosslinking degree based on the O/N atomic ratio obtained from XPS measurements. Since XPS probes only the top ~10 nm of the membrane surface, the calculated crosslinking degree may not accurately represent the overall structure of the entire polyamide selective layer, especially for structurally heterogeneous membranes. Indeed, as shown in our data (Supplementary Fig. 8), the calculated crosslinking degree can even yield physically unreasonable values below 0% (Using the measured O/N ratio of 2.03,

the crosslinking degree was calculated according to X=(2−O/N)/(O/N)=-1.4%), highlighting the limitations and potential uncertainty of this estimation method.

In contrast, the etched samples exhibited nearly identical O/N ratios (0.67 for C-NF and 0.69 for A-NF), indicating comparable internal crosslinking degrees. This result suggests that crosslinking is not the dominant factor governing the permeability difference between the two membranes, and that the enhanced water transport of A-NF should primarily arise from other structural characteristics of the selective layer.

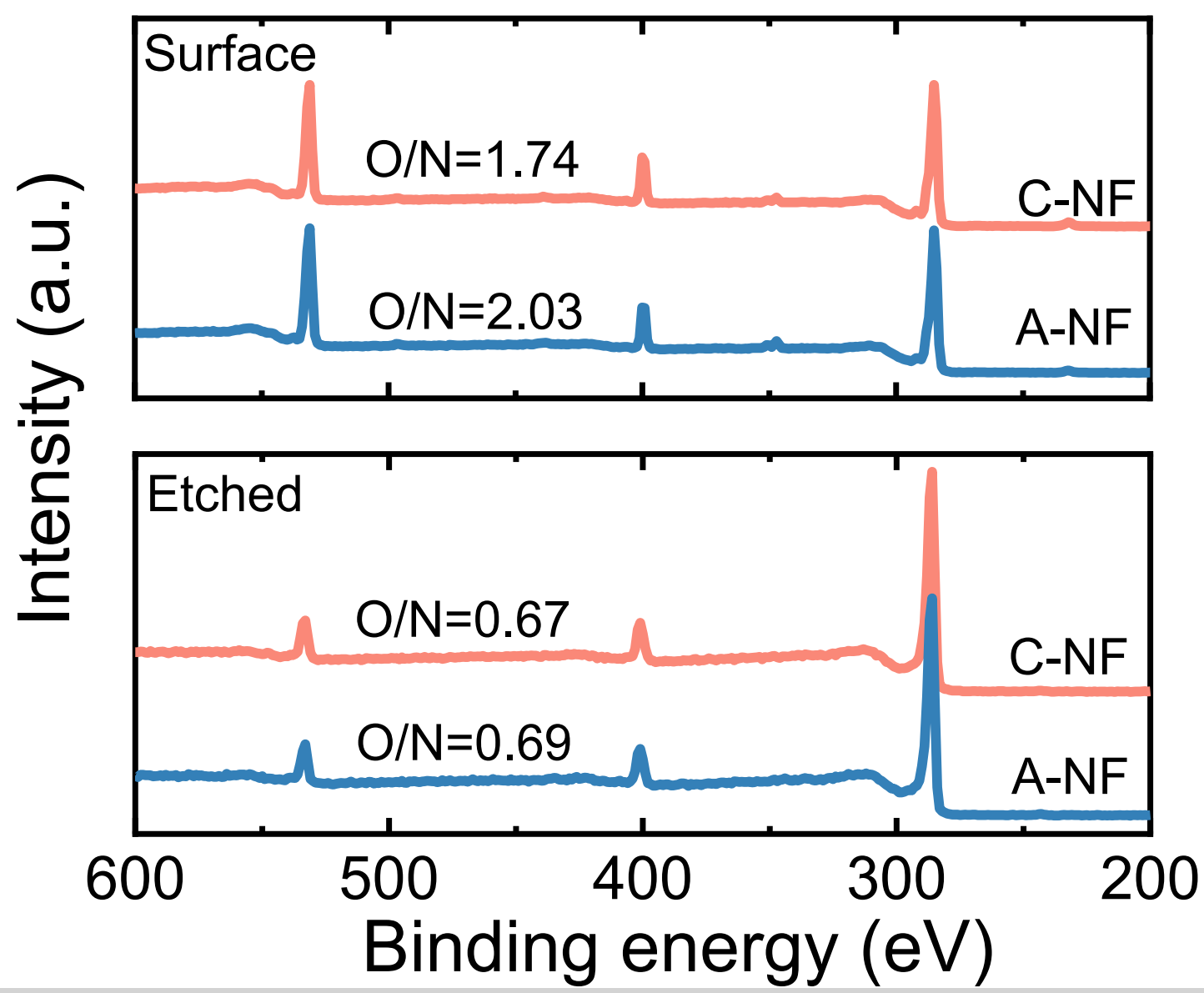

Supplementary Fig. 8: The XPS spectra of A-NF and C-NF surfaces before and after etching.

Therefore, our results suggest that membrane transport performance is not governed solely by the apparent XPS-derived crosslinking degree. Instead, the intrinsic nanoscale morphology and internal structural organization of the polyamide layer, particularly the presence or absence of crystalline domains, may play a more dominant role in determining water transport behavior in this system.

We attribute this improvement primarily to the elimination of the internal crystalline/ordered regions within the polyamide layer. These crystalline domains are generally considered to impose higher transport resistance and hinder the formation of efficient water transport pathways. Their suppression in A-NF likely results in a more favorable transport structure with lower intrinsic resistance to water permeation.

Based on the above discussion, we have revised the manuscript to further strengthen and clarify our viewpoint as follows:

Line 236: A higher oxygen-nitrogen ratio usually indicates a more porous structure in polyamides [26]. However, it should be noted that the surface O/N ratio may not reliably

reflect the intrinsic crosslinking degree of the selective layer, since XPS primarily probes only the outermost surface region, where oxygen-rich terminal groups are more likely to accumulate. Internal XPS analysis shows that A-NF and C-NF have similar oxygen-nitrogen ratios (**Supplementary Fig. 8**), indicating that their bulk chemical composition and crosslinking density remain essentially unchanged..

Line 271: To further decouple the thickness effect, the water permeance was normalized by the corresponding selective-layer thickness (**Supplementary Fig. 15**). Notably, the thickness-normalized water permeance of A-NF remained approximately twofold higher than that of C-NF, indicating that the enhanced water transport cannot be solely attributed to the reduced selective-layer thickness.

3-For polymers with linear configuration, crystallization could be triggered upon applying external pressure for prolonged time. While for polyamide, will this also occur after long-term filtration?

Our Response:. Thank you for this insightful question. From the perspective of polymer physics, although linear polymers may undergo pressure-induced crystallization under prolonged external stress, such behavior is much less likely to occur in highly crosslinked polyamide selective layers during long-term filtration.

First, the polyamide selective layer formed by interfacial polymerization possesses a densely crosslinked three-dimensional network structure rather than a flexible linear chain configuration. The mobility and rearrangement capability of the polymer chains are therefore strongly restricted. Pressure-induced crystallization generally requires sufficient segmental mobility for polymer chains to reorganize into ordered domains, which is difficult for highly crosslinked polyamide networks.

In addition, aromatic polyamide selective layers generally possess high mechanical stiffness and strong resistance to hydraulic compaction, which suppress chain packing and structural rearrangement under applied pressure [1]. Under typical nanofiltration or reverse osmosis operating pressures, the deformation of the polyamide network is generally limited to elastic compression rather than large-scale molecular rearrangement required for crystallization.

Based on these considerations, we believe that the ordered or crystalline regions observed in our work are more likely formed during the rapid interfacial polymerization process itself rather than generated gradually during long-term filtration operation.

[1] Wu, J. et al. Ultrahigh pressure compaction-resistant thin film crosslinked composite reverse osmosis membranes. Nat Commun 16, 8165 (2025). https://doi.org/10.1038/s41467-025-63639-0

4-Since the dual strategy could also reduce the polyamide thickness, which was beneficial for water passing through, then which effect would dominate the great enhancement in

flux, thickness reduction or amorphous structure? How to quantify the contribution of each factor?

Our Response:. Thank you for this important question. The reduction in polyamide thickness indeed facilitates water transport by shortening the transport pathway. However, our results suggest that the enhancement in water flux cannot be explained solely by thickness reduction.

By normalizing the water permeability with respect to the polyamide thickness, we found that the normalized water permeability of A-NF still increased by approximately twofold compared with C-NF. This result indicates that the intrinsic transport capability of the polyamide layer itself was significantly improved, even after eliminating the contribution from thickness variation. Therefore, the amorphous structure appears to play a dominant role in the observed flux enhancement.

We believe that the suppression of crystalline or ordered domains reduces the internal transport resistance within the polyamide network and generates more continuous and less hindered pathways for water transport. In contrast, thickness reduction mainly decreases the diffusion distance. Both factors contribute positively to permeability enhancement, but the thickness-normalized analysis demonstrates that structural evolution toward a more amorphous morphology provides an additional and substantial contribution. We have added further discussion in the revised manuscript to strengthen and clarify this point.

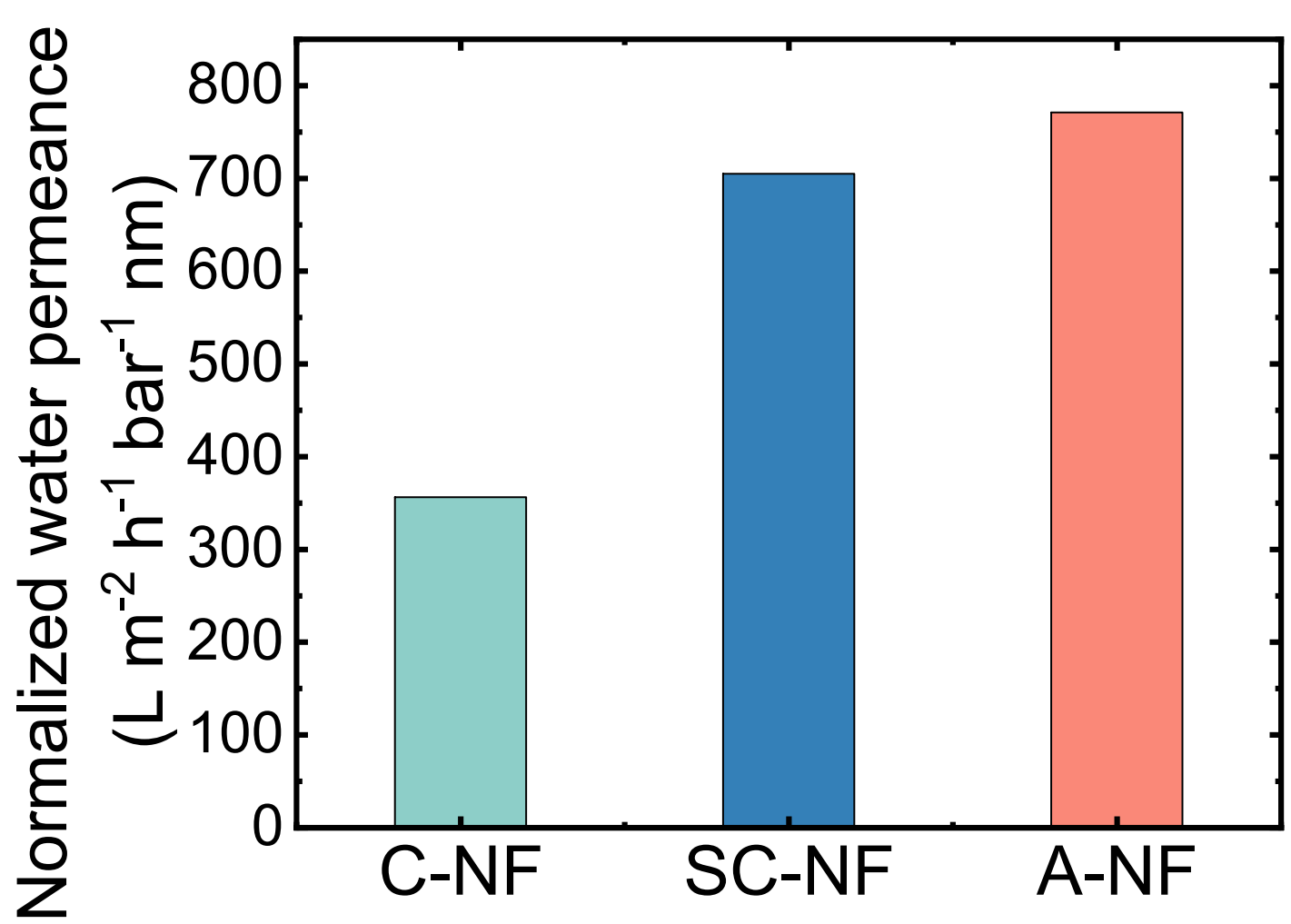

Supplementary Fig. 15: Thickness-normalized water permeance of C-NF, SC-NF, and A-NF. The water permeance was normalized by multiplying the measured permeance by the corresponding selective-layer thickness.

Line 270: To further decouple the thickness effect, the water permeance was normalized by the corresponding selective-layer thickness (**Supplementary Fig. 15**). Notably, the thickness-normalized water permeance of A-NF remained approximately

twofold higher than that of C-NF, indicating that the enhanced water transport cannot be solely attributed to the reduced selective-layer thickness.

5-Although the high permeability of A-NF to Ca^{2+} alleviates membrane fouling contamination, it will result in a large amount of Ca^{2+} migrating to the product water side. In a typical ZLD process, should the issue of new scaling problems caused by this in the subsequent evaporation crystallization unit be considered, as well as the potential problems it brings to the system stability and product purity?

Our Response:. Thank you for this insightful question. We agree that if a feed stream contains a high concentration of Ca^{2+}, the increased passage of Ca^{2+} through A-NF could potentially lead to scaling during the subsequent evaporation and crystallization process, as well as negatively affect salt purity and long-term system stability.

However, in conventional ZLD processes, strict calcium removal is typically required before the wastewater enters the membrane system in order to avoid severe scaling throughout the entire treatment train. Industrial pretreatment processes such as chemical softening and lime-soda precipitation are commonly employed to reduce Ca^{2+} concentration to a safe operating level before membrane concentration.

In our study, we measured the Ca^{2+} concentration in the coal chemical wastewater concentrate used for testing. As shown in the revised manuscript, the Ca^{2+} concentration was only approximately 8 mg L^{-1}, which is extremely low compared with the concentrations of NaCl and Na_2SO_4 in the system. Therefore, the amount of Ca^{2+} transferred to the permeate side is very limited and is unlikely to induce significant scaling or noticeably affect the purity of the recovered salts during the evaporation crystallization process. To clarify this point, we have added additional discussion in the revised manuscript as follows:

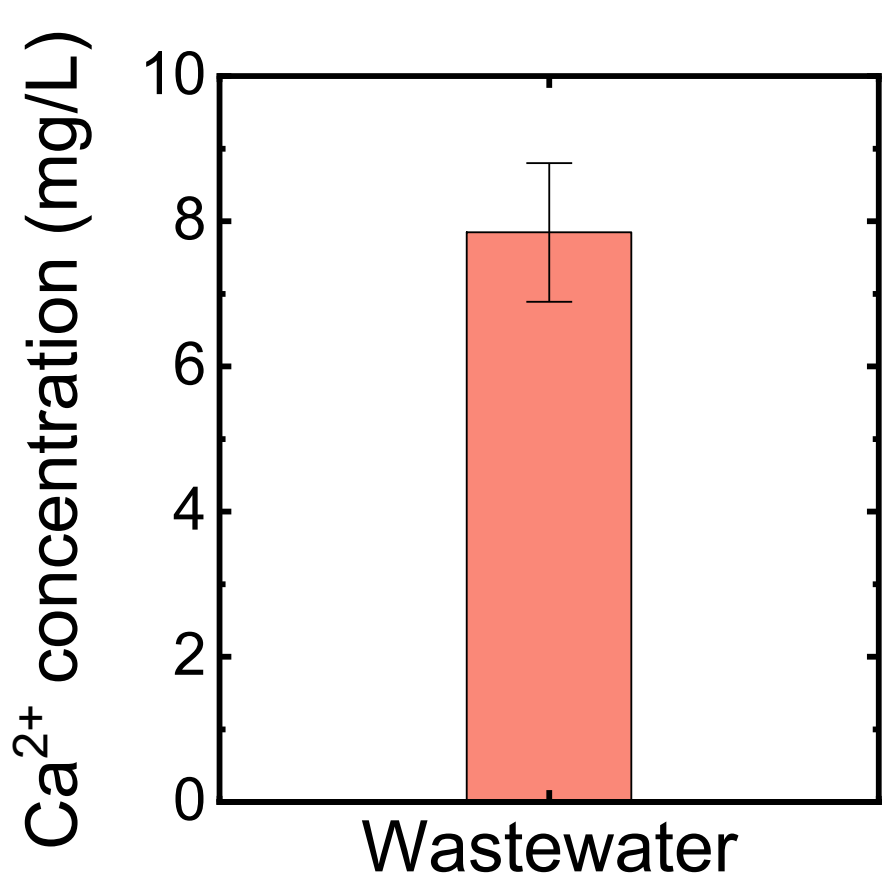

Supplementary Fig. 26: Ca^{2+} concentration in the actual coal chemical wastewater used in this study.

Line 382: It should be noted that the coal chemical wastewater concentrate was subjected to a Ca^{2+} removal pretreatment before membrane filtration, reducing the residual Ca^{2+} concentration to approximately 8 mg/L (**Supplementary Fig. 26**).

6-Is the low-temperature interface polymerization method for membranes applicable to other monomer systems? Does the applicability of this strategy need to be customized and designed based on the specific chemical properties of different monomer structure systems?

Our Response:. Thank you for this insightful comment. To evaluate the general applicability of the low-temperature interfacial polymerization strategy, in addition to the PIP-based system discussed in the main text, we further extended this approach to other aqueous-phase monomers, including polyethyleneimine (PEI) and m-phenylenediamine (MPD).

The results demonstrate that the ultralow-temperature interfacial polymerization strategy is also effective in these different monomer systems. Specifically, in the PEI-based interfacial polymerization system, the ultralow-temperature condition increased water permeability by approximately 50.1% while also delivering a noticeable improvement in salt rejection performance from 89.3% to 94.6%. In contrast, for the MPD-based system, the water flux increased by 51% under the same strategy.

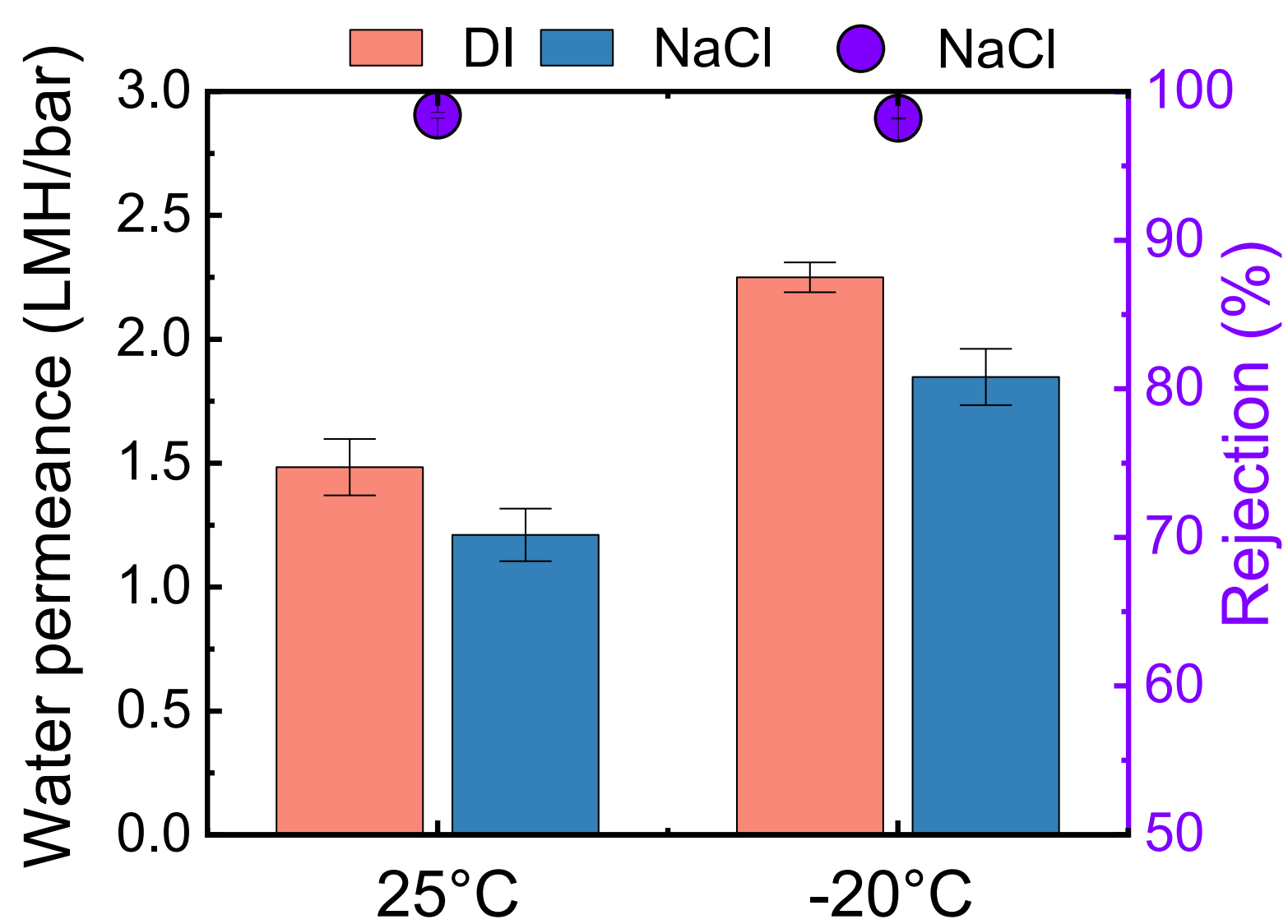

Supplementary Fig. 16: Separation performance of MPD-based nanofiltration membranes prepared by interfacial polymerization at different temperatures (25 °C and −20 °C), including water permeance and NaCl rejection. Applied pressure: 20 Bar, NaCl concentration: 2 g/L

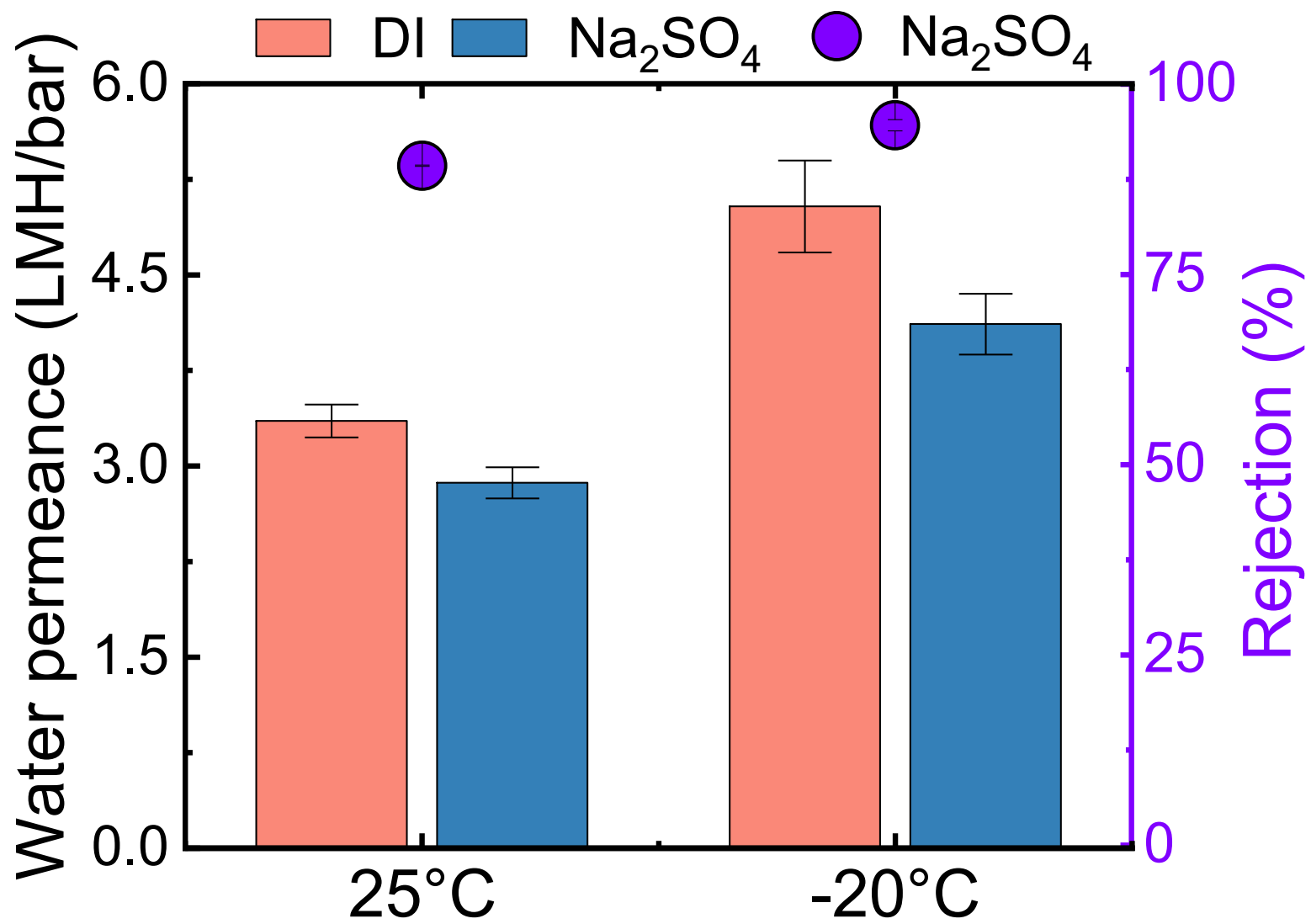

Supplementary Fig. 17: Separation performance of PEI-based nanofiltration membranes prepared by interfacial polymerization at different temperatures (25 °C and −20 °C), including water permeance and Na_2SO_4 rejection. Applied pressure: 6.9 Bar, Na_2SO_4 concentration: 10mM

These results suggest that the low-temperature interfacial polymerization method is broadly applicable to various interfacial polymerization systems for the fabrication of polyamide TFC membranes. Moreover, the strategy appears to be particularly effective for PIP-based nanofiltration membrane fabrication, where a more pronounced enhancement in water permeability and separation performance can be achieved. We have incorporated the additional interfacial polymerization systems and the corresponding discussion into the revised manuscript.

Line 276: The applicability of the ultralow-temperature interfacial polymerization strategy was further validated using PEI- and MPD-based systems (detailed fabrication procedures for these additional membrane systems are provided in the **Supplementary Methods**). As shown in **Supplementary Figs.16-17**, both systems exhibited substantially improved water permeability under ultra-low-temperature conditions. In particular, the PEI-based membrane achieved a 50.1% increase in water permeance together with improved salt rejection (89.3% to 94.6%), while the MPD-based membrane showed a 51% increase in water flux. These results confirm the broad applicability of this strategy across different interfacial polymerization systems, although the most pronounced permeability–selectivity enhancement was observed for the PIP-based nanofiltration membrane.

7-The basal membrane was first immersed in the aqueous phase at -20°C, and then transferred to the oil phase at room temperature for interface polymerization. This operation process could introduce a significant temperature gradient. Does the author quantitatively evaluate the impact of this temperature transition from the following three aspects: (1) the concentration distribution process of PIP monomers in the aqueous phase boundary layer; (2) the local adsorption conformation and spatial distribution of

TMC at the interface; (3) the spatial uniformity of the crosslinking density of the polyamide network? It is recommended to consider the interference of this thermal-dynamic coupling process on the stability of the interface, which affects the controllability and batch reproducibility of the amorphous membrane structure.

Our Response:. We thank the reviewer for this insightful comment. We agree that a temperature gradient is inevitably introduced when the substrate, pre-equilibrated in the aqueous phase at −20 °C, is transferred into the organic phase at room temperature. Indeed, the central objective of this work is to develop a water-phase temperature regulation strategy for the fabrication of high-performance amorphous nanofiltration membranes. In this approach, it is critical that the substrate remains at an ultralow temperature immediately before contact with the organic phase, whereas the subsequent temperature evolution during interfacial polymerization is considerably more complex.

To verify the thermal state of the substrate prior to interfacial polymerization, infrared thermal imaging was employed to monitor the substrate temperature. The results confirmed that the substrate remained within the ultralow-temperature range before immersion into the organic phase, ensuring the effectiveness of the proposed temperature-regulation strategy.

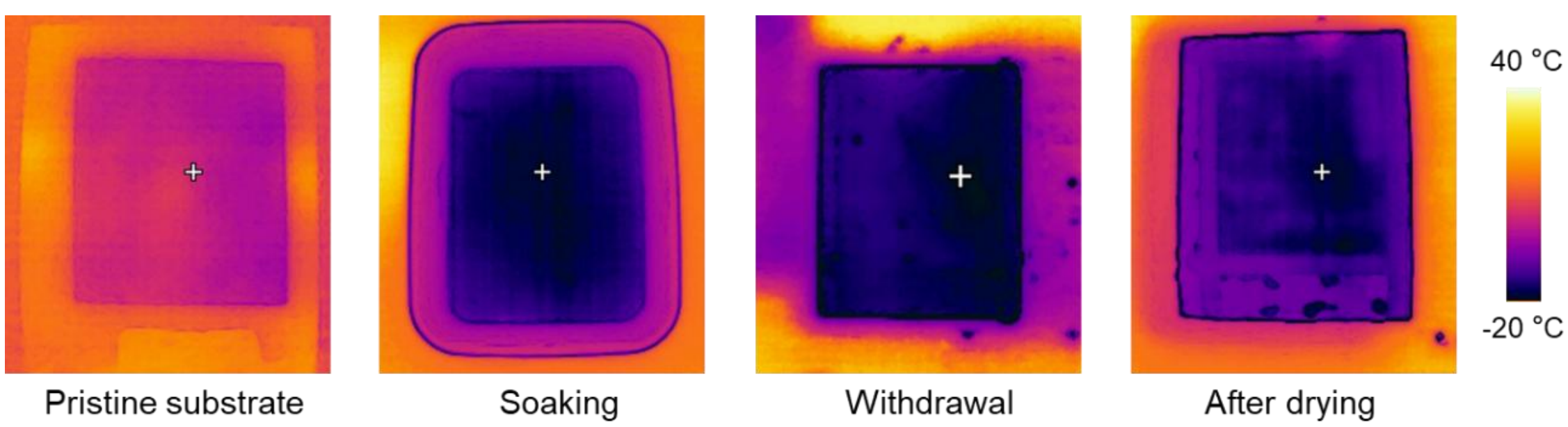

Supplementary Fig. 31: Infrared thermal images of the membrane substrate at different stages of the ultralow-temperature treatment

Line 478: The membranes were immersed in 1 wt% PIP and 30% methanol aqueous solution at -20°C for 2 min, drained, and then contacted with 0.15 wt% TMC in *n*-hexane for 30 s to form the polyamide selective layer. Infrared thermal imaging measurements confirmed that the membrane substrate retained an ultralow temperature immediately prior to immersion in the n-hexane phase (**Supplementary Fig. 31**).

Regarding the concentration distribution of PIP in the aqueous boundary layer and the local adsorption configuration/spatial distribution of TMC at the interface, these processes occur within a transient and nanoscale interfacial region during rapid interfacial polymerization, making their direct experimental characterization highly challenging. In the present work, we therefore focused on evaluating the resulting polyamide structure rather than tracking these dynamic interfacial processes in situ.

Specifically, due to the self-limiting nature of interfacial polymerization, the crosslinking density is typically not completely uniform across the thickness of the polyamide layer, with the outer surface generally exhibiting a lower crosslinking degree than the inner region. We characterized the chemical structure of the polyamide layer by XPS (XPS provides primarily qualitative information on surface crosslinking characteristics). The XPS results therefore reflect the relatively lower crosslinking degree at the membrane surface.

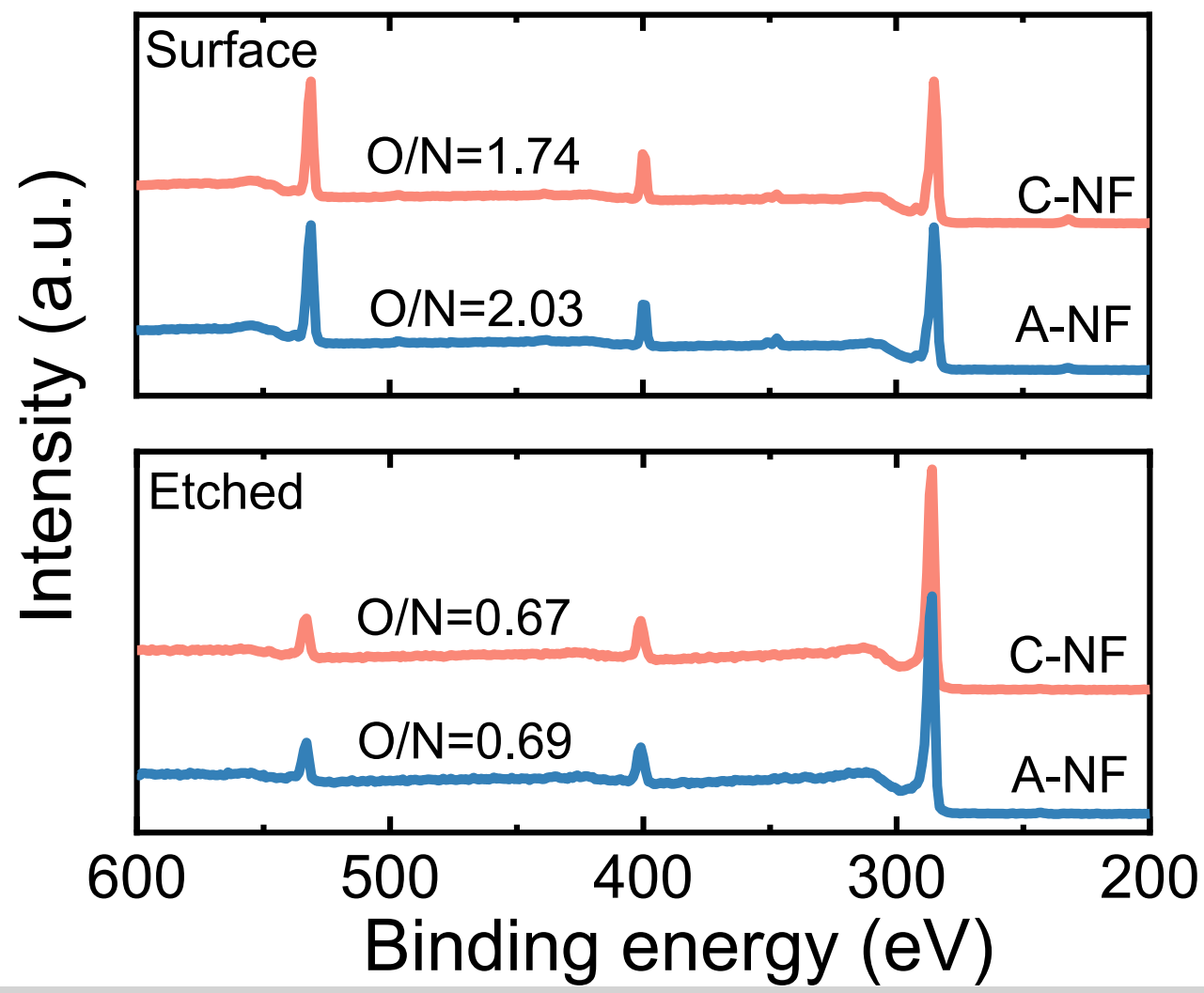

Supplementary Fig. 8: The XPS spectra of A-NF and C-NF surfaces before and after etching.

In addition, to ensure the reliability and reproducibility of the amorphous membrane structure, the crystallographic characterization was performed on polyamide samples obtained by peeling the active layers from approximately 1 m^2 of membranes prepared in multiple independent batches. Therefore, the reported crystallographic results represent the average characteristics of multiple membrane batches, demonstrating good batch-to-batch reproducibility of the amorphous structure obtained through the proposed strategy.

8-The claim of a > 400% increase in water permeability should be accompanied by a comparative analysis with existing literature to contextualize the performance enhancement.

Our Response:. Thank you for this valuable comment. We would like to clarify that the reported >400% increase in water permeability refers specifically to the enhancement relative to the control membrane prepared under otherwise identical fabrication conditions within the same membrane system. Since membrane performance is highly dependent on factors such as monomer chemistry, interfacial polymerization conditions, substrate properties, and post-treatment protocols, direct comparison of relative permeability enhancement across different membrane fabrication strategies reported in the literature may not be meaningful or directly

comparable. To provide broader context, we instead compared the absolute separation performance of A-NF, including both water permeance and separation factor, with representative state-of-the-art nanofiltration membranes reported in the literature. The comparison shows that A-NF achieves competitive and leading overall performance among reported nanofiltration membranes.

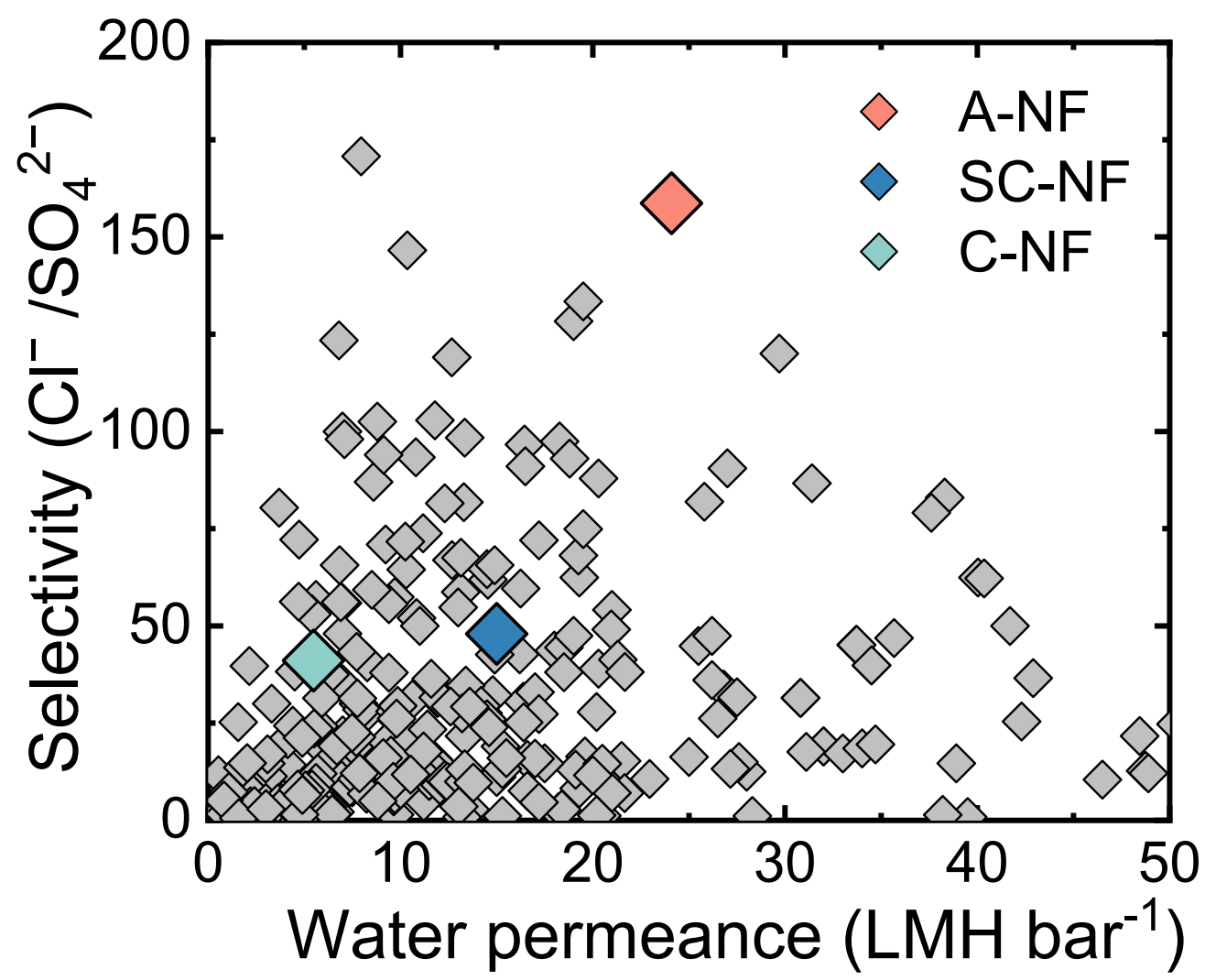

Fig. 3b: Summary of water permeance as well as NaCl/Na_2SO_4 selectivity of NF membranes reported in literature and in market. The gray diamond symbols represent literature data points corresponding to the membranes listed in Supplementary Table 1.

9-The manuscript often refers to "ultra-smooth" surfaces and "defect-free" structures, but more detailed quantitative data should be provided to substantiate these claims. How are ultra-smooth surfaces defined internationally?

Our Response:. Thank you for this valuable comment. In the original manuscript, the term “ultra-smooth” was used because the surface roughness of A-NF was significantly reduced compared with that of C-NF according to the AFM characterization results. In addition, the term “defect-free” was used because the proposed ultralow-temperature liquid–liquid interfacial polymerization strategy effectively eliminates the crystalline regions within the polyamide layer. In our view, these crystalline regions can be regarded as structural defects in the polyamide matrix, as they hinder efficient water transport through the membrane.

We appreciate the reviewer’s suggestion regarding the terminology. We have revised the description from “ultra-smooth” to “smooth” throughout the manuscript to avoid potential overstatement.

10-This work focuses on water permeability and salt rejection, but long-term stability and fouling resistance are only briefly addressed. Furthermore, 800 min long-term operation time is too short and not convincing.

Our Response:. Thank you for this valuable comment. We would like to respectfully clarify that a 120-h continuous operation test had already been included in the original manuscript to evaluate the long-term operational stability of the A-NF membrane, although this may not have been sufficiently emphasized in the previous version.

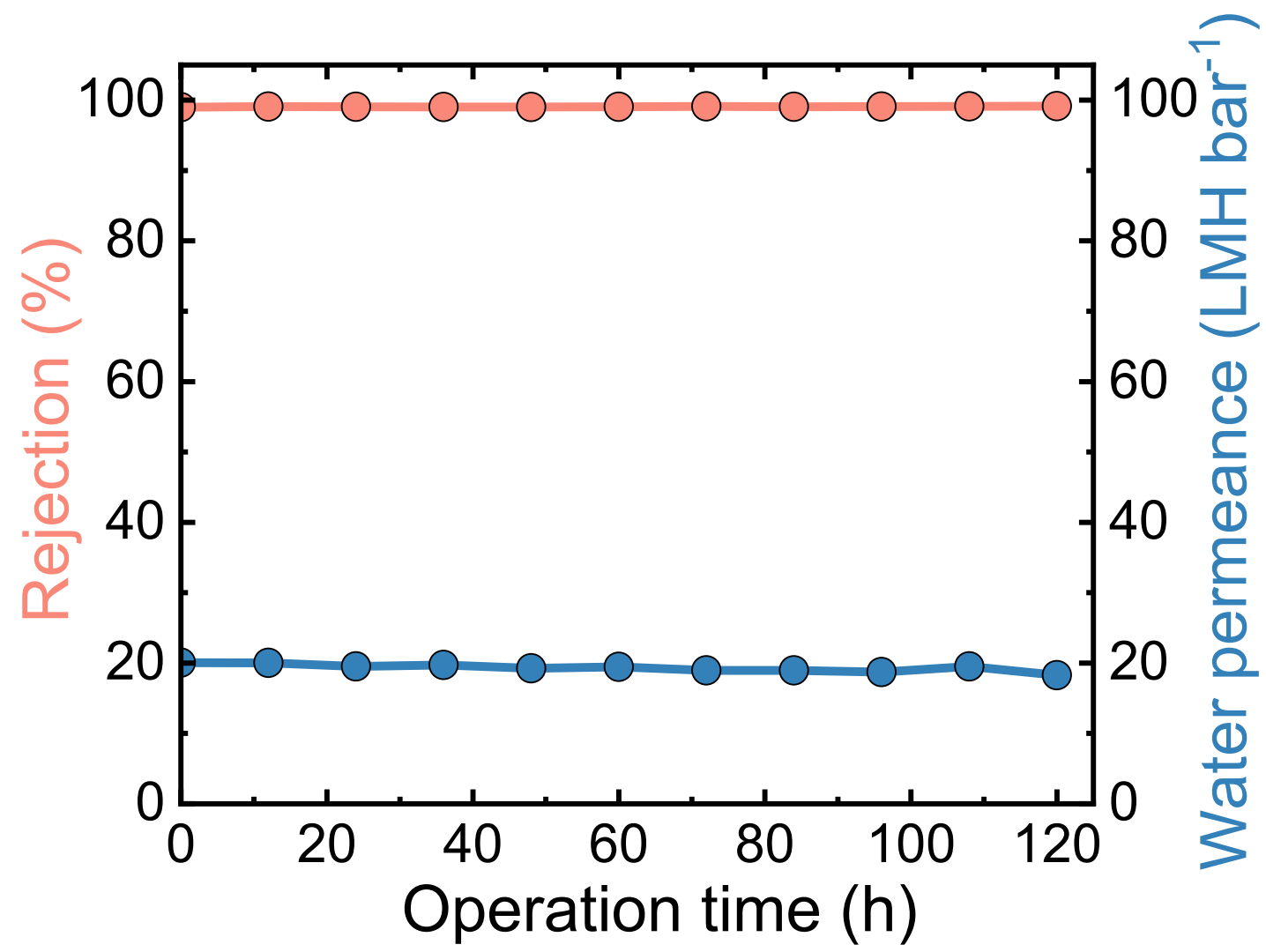

Supplementary Fig. 18: Continuous separation performance of A-NF.

In addition, the antifouling performance was systematically investigated using both organic fouling and inorganic scaling systems. Specifically, humic acid was employed as a model organic foulant, while an inorganic scaling system was used to evaluate scaling resistance. Furthermore, QCM-D analysis was conducted to provide in situ insights into the fouling behavior and interfacial interactions on the membrane surface.

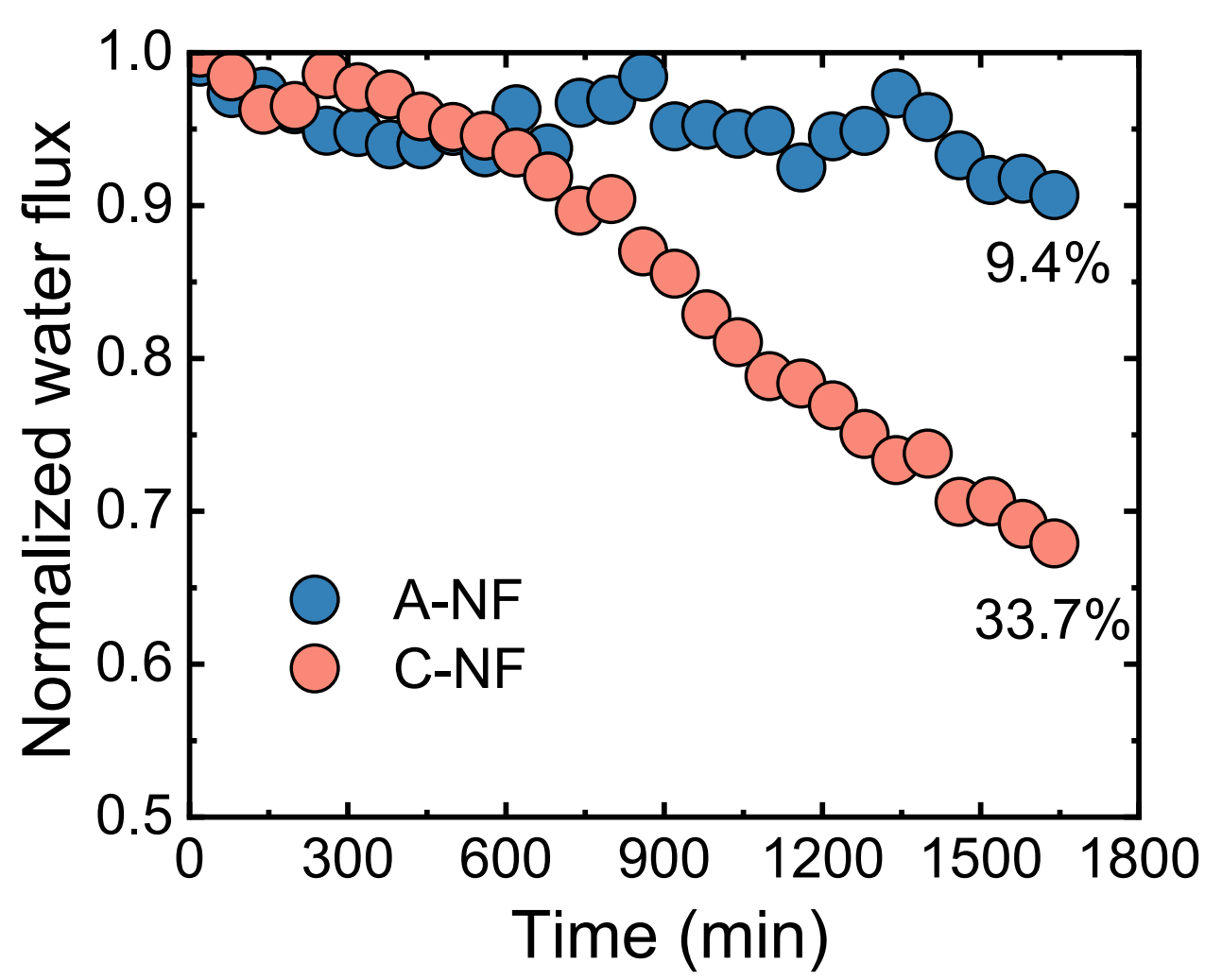

Fig. 3e: Anti-scaling performance of A-NF and C-NF with feed solution containing 15 mM $CaCl_2$, 10 mM Na_2SO_4, and 10 mM NaCl; the gypsum ($CaSO_4 \cdot 2H_2O$) saturation index (SI) of this solution is 0.54.

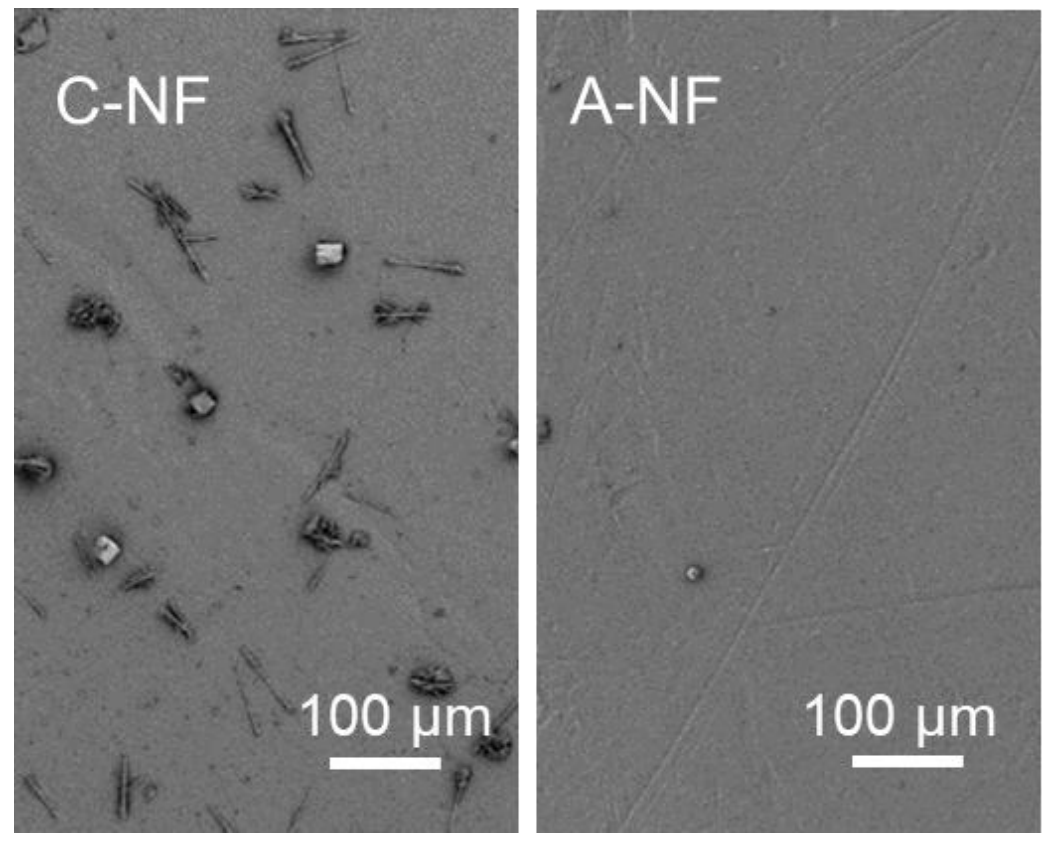

Supplementary Fig. 23: Surface SEM images of C-NF and A-NF after long-term anti-scaling test.

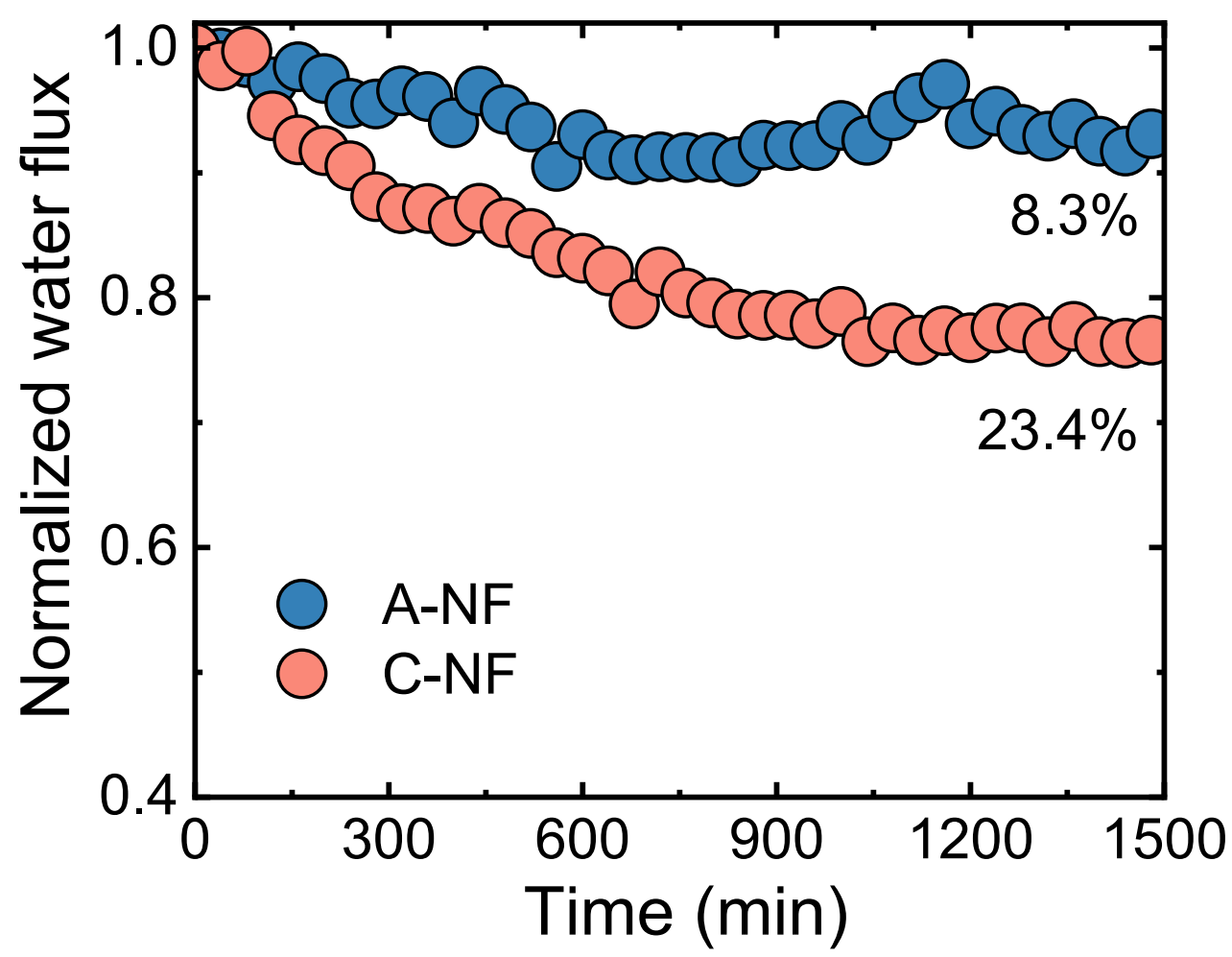

Fig. 3f: Anti-organic fouling performance of A-NF and C-NF. Humic acid concentration: 50 mg L^{-1}. $CaCl_2$ concentration: 50 mg L^{-1}.

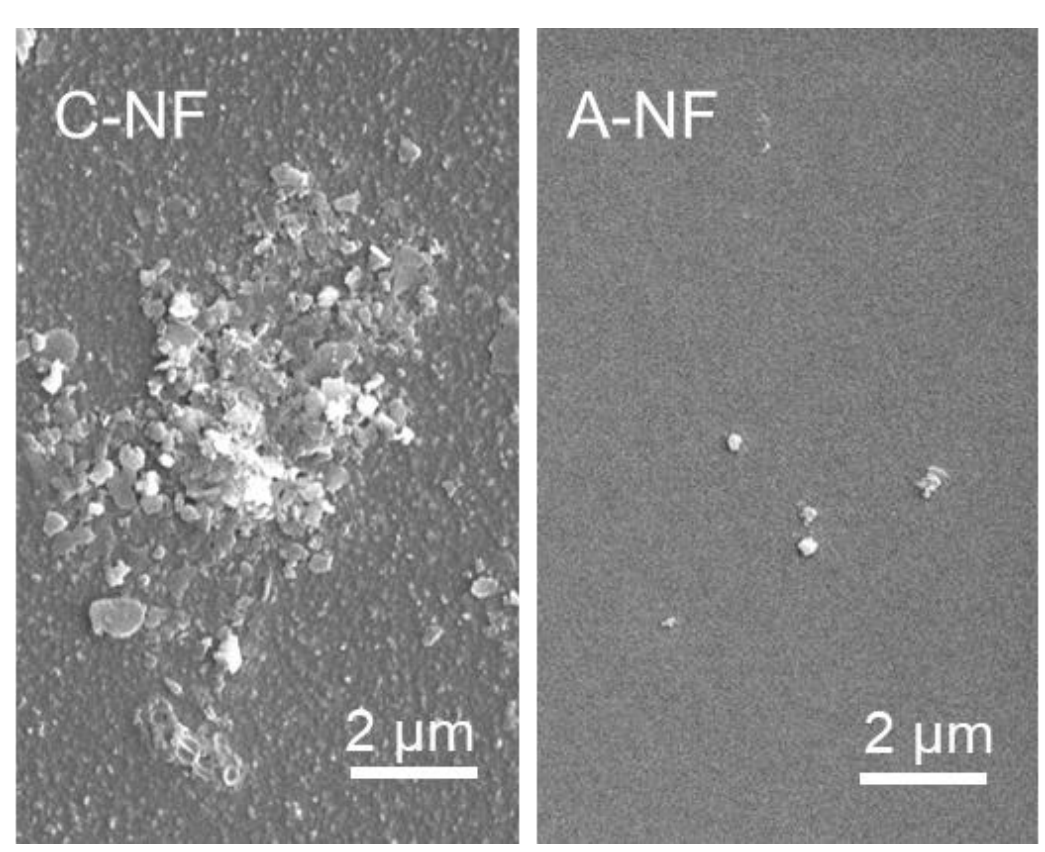

Supplementary Fig. 25: Surface SEM images of C-NF and A-NF after long-term anti-fouling test.

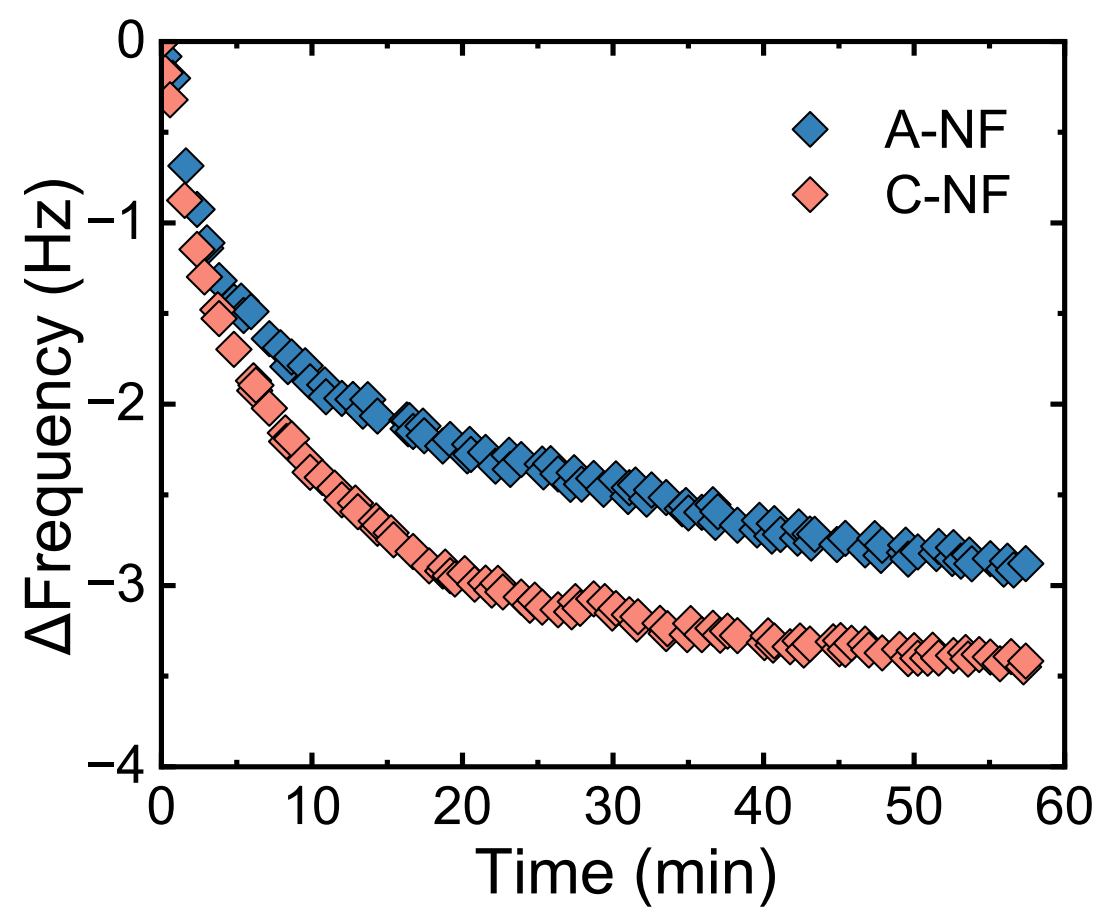

Supplementary Fig. 24: Real time in-situ monitoring of humic acid deposition (i.e., fouling) using QCM.

Moreover, during the treatment of real coal chemical wastewater containing complex foulants such as organic matter and silica species, the A-NF membrane still exhibited excellent antifouling performance and operational stability. To avoid misunderstanding and improve clarity, we have revised the manuscript to further strengthen and highlight the discussion on the long-term stability and antifouling properties of the membrane:

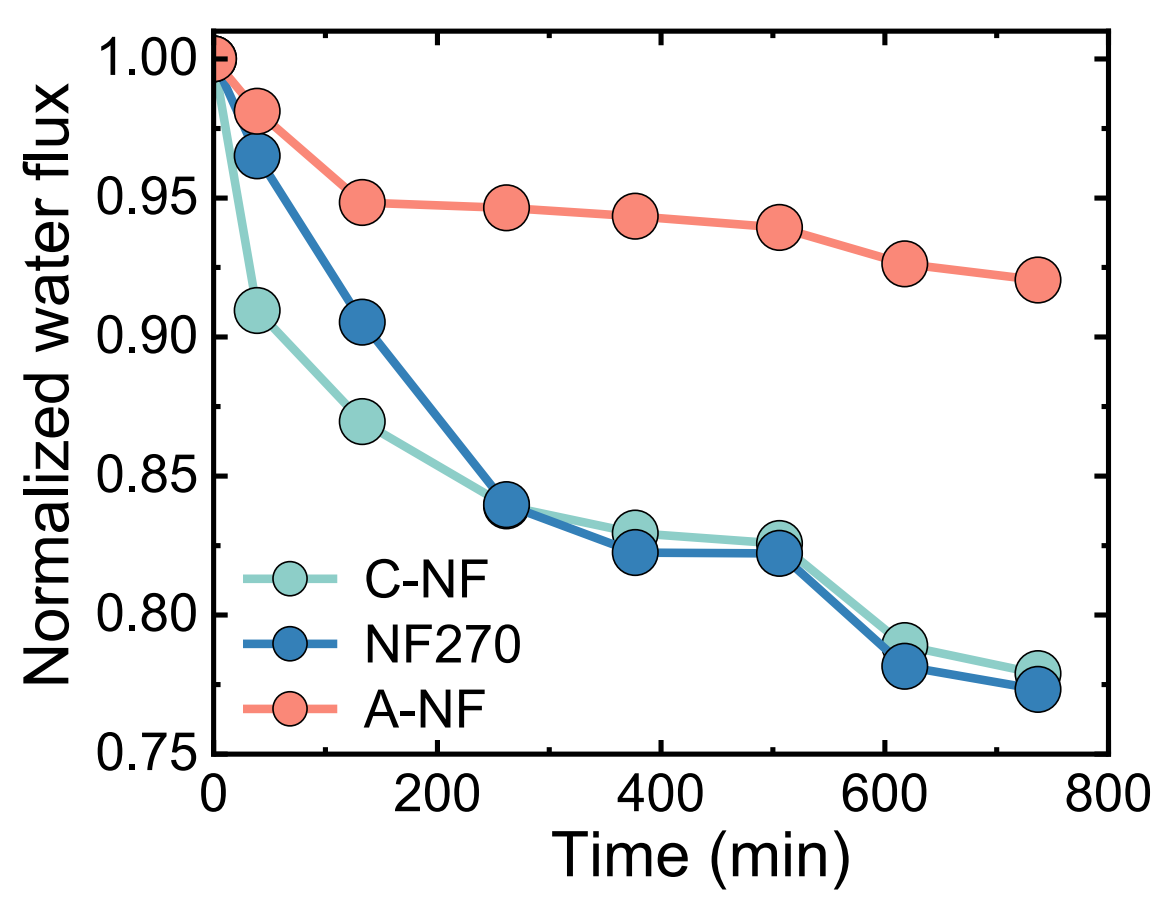

Fig. 4c: Comprehensive anti-fouling performance evaluation of A-NF, C-NF and NF270 during long-term operation.

Line 301: The stability of NF membranes under continuous operation is an important parameter for evaluating their practical viability. As shown in **Supplementary Fig. 18**, the Na_2SO_4 rejection of A-NF remained stable during 120 hours of continuous operation.

Line 342: NF membrane fouling leads to a sharp decline in water flux and necessitates higher operating pressures, which drives up energy consumption and costs while compromising both separation efficiency and system longevity. The smooth surface

of A-NF results in lower adhesion of foulants. Furthermore, the high permeability of A-NF to Ca^{2+} alleviates inorganic scaling as well as organic fouling.

11-In the MD simulations, how did the authors determine the degree of cross-linking? Do the experimental crosslinking data align with the simulation results? Please provide details.

Our Response:. Thank you for this insightful comment. We would like to respectfully clarify that the MD simulations in this work were not intended to model the polyamide network structure or determine the degree of cross-linking. Instead, the simulations were designed to investigate the diffusion and mass transfer behaviors of monomers under different temperature conditions, thereby providing molecular-level insights into how the low-temperature process influences interfacial polymerization. Therefore, no simulated cross-linking degree was calculated or compared with experimental crosslinking data in the current study.

12-In addition to the MD simulations of interfacial polymerization, the authors should conduct pressure-driven desalination simulations to better illustrate the desalination mechanism.

Our Response:. Thank you for this valuable suggestion. We agree that pressure-driven desalination simulations could further strengthen the mechanistic understanding of this work. However, performing such simulations for the present system remains highly challenging due to the limitations of current polyamide membrane modeling approaches.

As mentioned in the reviewer's previous comment, most existing MD simulation frameworks for polyamide membranes are constructed based on amorphous cross-linked network models, where the membrane structure is primarily described by the degree of cross-linking. In contrast, the key difference between A-NF and C-NF in our work is believed to originate from the regulation of crystalline regions within the polyamide layer rather than solely from differences in cross-linking density.

At present, there is still a lack of well-established methodologies capable of accurately constructing and characterizing crystalline polyamide structures formed during interfacial polymerization. Therefore, conventional pressure-driven desalination simulations based on amorphous models may not accurately capture the structural characteristics and transport behavior revealed in this work, and could potentially lead to misleading conclusions.

Nevertheless, we fully agree that this is an important future research direction. Indeed, the discovery and regulation of crystalline regions in polyamide membranes presented in this study may provide a new perspective for the development of next-generation membrane simulation models.

13-The hypothesis about the role of methanol in enhancing the diffusion of PIP should be supported by experimental verification.

Our Response:. We thank the reviewer for this insightful comment. To experimentally verify the role of methanol in PIP diffusion, we performed 2D DOSY measurements to determine the diffusion coefficients of PIP in pure aqueous solution and in 30% methanol–water solution. As shown in Supplementary Fig. 2, the diffusion coefficient of PIP in the 30% methanol–water system is lower than that in pure water, indicating that the bulk diffusion of PIP is somewhat slower in the presence of methanol.

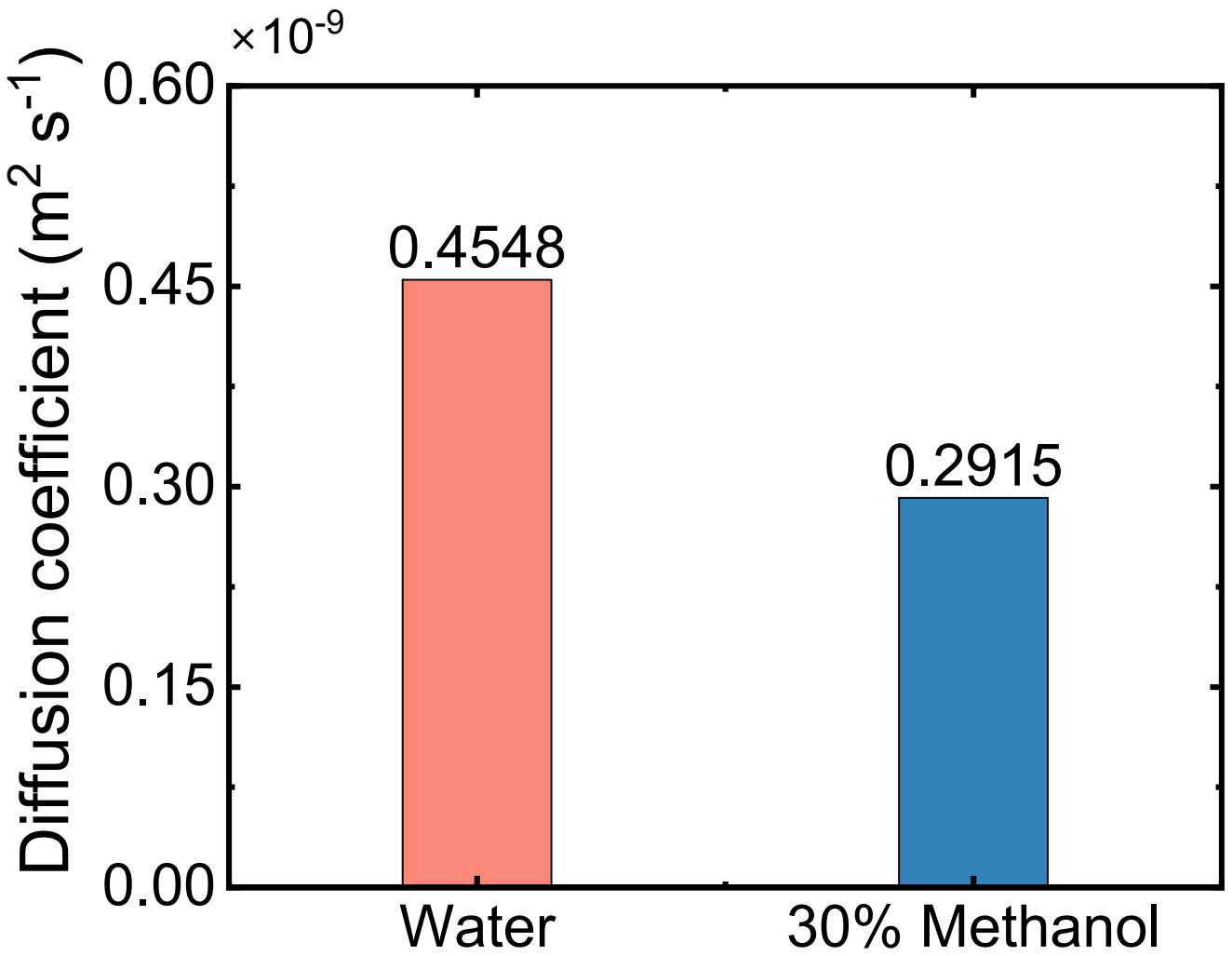

Supplementary Fig. 2: The diffusion coefficient of PIP in 30% methanol aqueous solution and water as quantified by 2D DOSY NMR.

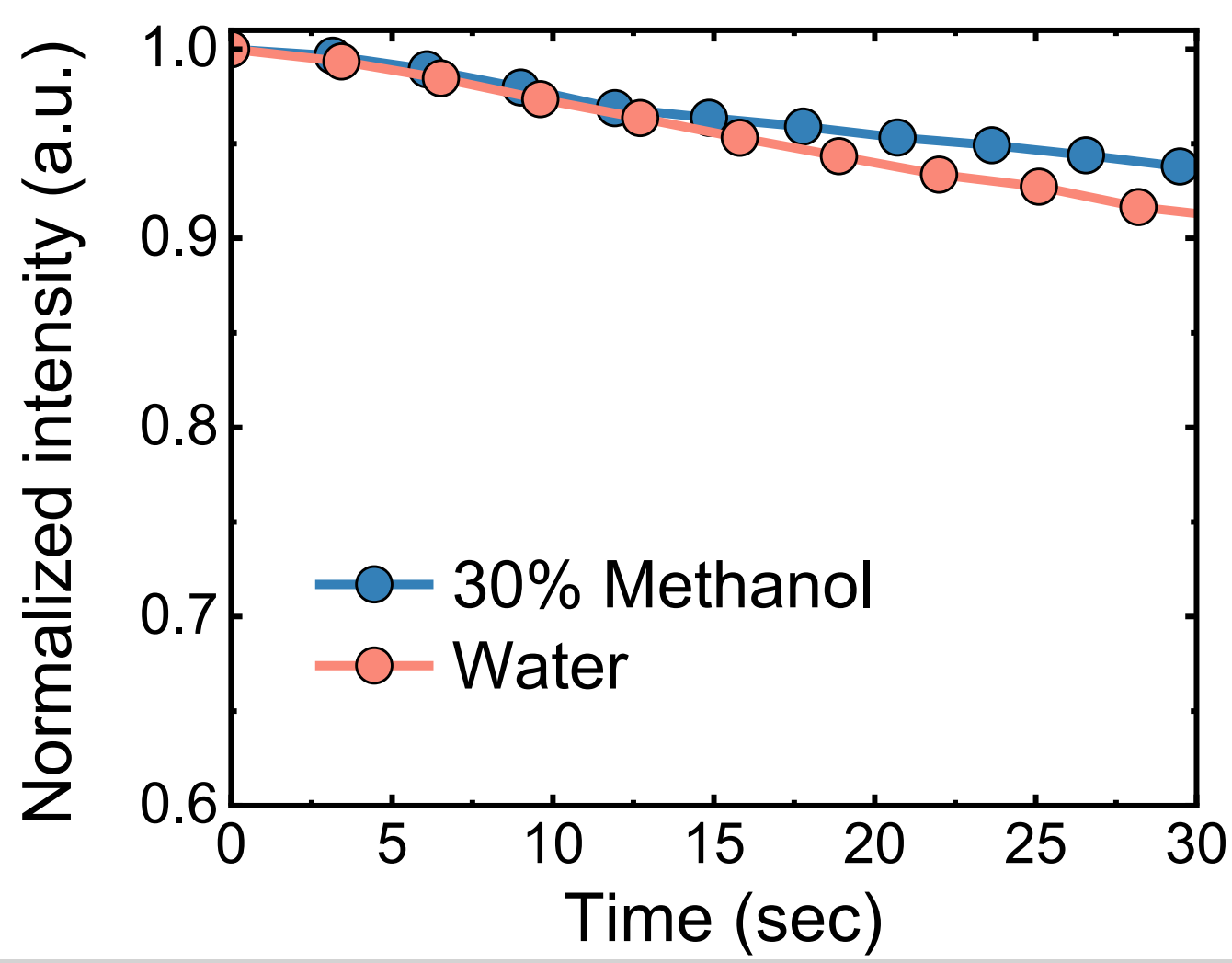

Supplementary Fig. 3: Changes of PIP concentration over time during interfacial polymerization in 30% methanol aqueous solution and water.

Interestingly, when n-hexane was introduced to construct the interfacial diffusion system (Supplementary Fig. 3), the concentration of PIP diffusing into the *n*-hexane

phase within the same time period was comparable for the pure aqueous system and the 30% methanol–water system. These results suggest that methanol likely facilitates the interfacial transfer of PIP by altering the physicochemical properties of the aqueous–organic interface, such as reducing interfacial tension and modifying interfacial molecular interactions. This experimental evidence supports our hypothesis that methanol promotes the effective transport of PIP across the water/hexane interface, thereby accelerating interfacial polymerization. Based on these supplementary experimental results, we have revised the corresponding discussion in the manuscript to clarify this mechanism:

Line 146: Experimental results further support this interpretation. Although 2D DOSY measurements showed a lower bulk diffusion coefficient of PIP in the 30% methanol–water solution than in pure water (**Supplementary Fig. 2**), the decrease in PIP concentration in the aqueous phase during the interfacial diffusion experiments was comparable for the two systems (**Supplementary Fig. 3**). This result suggests that the reduced bulk diffusivity of PIP in the methanol-containing solution does not lead to a corresponding reduction in its overall transport toward the reaction interface. Therefore, the influence of methanol on interfacial polymerization is unlikely to be governed solely by bulk diffusion. Instead, methanol may modify the interfacial environment and molecular interactions at the water/organic boundary, consistent with the MD simulations and supporting its role in promoting interfacial polymerization.

14-At the end of this manuscript, the authors conducted a LCA analysis on industrial wastewater treatment, which was totally meaningless to me. Instead, the authors should focus more on assessing the energy consumption required to maintain the low temperature during large-scale production and its corresponding carbon footprint, comparing with traditional methods to further verify the scalability and actual energy cost of this technology. Moreover, the unit price of the A-NF membrane sheet prepared at -20°C should been included for comparison with the traditional NF membranes?

Our Response:. We thank the reviewer for this valuable comment. We agree that, compared with the general life cycle assessment of industrial wastewater treatment, a more direct evaluation of the energy consumption associated with low-temperature membrane fabrication is more relevant for assessing the practical scalability of our strategy. We would like to clarify that the original LCA analysis was intended to highlight the potential sustainability benefits arising from crystal-structure engineering of the membrane, particularly the improvements in selectivity, water permeance, and antifouling performance, which are highly relevant to practical industrial wastewater treatment. At the same time, we fully agree that a quantitative assessment of the additional cooling energy input is essential for evaluating the overall feasibility of this low-temperature fabrication strategy.

Specifically, due to the lack of access to industrial-scale operational energy consumption data, which are typically proprietary and commercially confidential, it is not currently possible to provide a comprehensive assessment of the overall energy consumption and carbon footprint for large-scale production. Therefore, based on a

simplified thermodynamic estimation, we focused on quantifying the additional cooling energy required for maintaining the low-temperature condition during the short-duration interfacial polymerization process. Based on a simplified thermodynamic estimation, cooling 100 L of precursor solution from 25 °C to −20 °C requires a theoretical cooling load of approximately 5.2 kWh. Considering a practical refrigeration coefficient of performance (COP) of 1.5–2.0, the corresponding actual electricity consumption is estimated to be 2.6–3.5 kWh per cooling cycle. Assuming a typical electricity carbon emission factor of 0.45–0.55 kg CO_2-eq kWh^{-1}, the associated carbon footprint is estimated to be approximately 1.2–1.9 CO_2-eq per batch. Considering that each batch can produce a substantial membrane area under practical fabrication conditions, the additional cooling energy and carbon footprint allocated per unit membrane area would be minimal, indicating that the overall increase in energy consumption and carbon emissions is very limited.

It should be noted that this simplified estimation does not account for heat exchange with the surrounding environment or other thermal losses during refrigeration. Consequently, the actual energy consumption may be somewhat higher than the calculated value. However, considering the relatively low cooling load, these factors are not expected to substantially alter the overall conclusion regarding the limited additional energy demand associated with the low-temperature fabrication process.

Rough calculation process:

Volume of precursor solution: V=100 L
Mass of solution: m～100 kg(assuming the density of the solution is approximately equal to that of water)
Specific heat capacity: $Cp=4.18$ kJ kg^{-1} K^{-1} (the specific heat capacity of a 30% methanol–water solution is slightly lower than that of pure water, and therefore this calculation provides a conservative estimation of the cooling load)

Temperature decrease: $\Delta T=25-(-20)=45$ °C
The theoretical cooling load is: $Q= m\ Cp\ \Delta T=$ 18810 kJ= 5.23 kWh

Considering a practical refrigeration coefficient of performance (COP) of 1.5–2.0:
$E=Q/COP=$ 2.62 kWh−3.49 kWh

Assuming a typical electricity emission factor: 0.45–0.55 kg CO_2-eq kWh^{-1}
The carbon footprint is: 1.2−1.9 kg CO_2-eq per batch

Regarding the membrane production cost, we would like to note that accurate industrial manufacturing cost data are generally proprietary and constitute core commercial information of membrane manufacturers, making it difficult to provide a precise quantitative comparison of unit price at this stage. Nevertheless, based on the limited additional cooling energy estimated above and considering that the proposed low-temperature fabrication strategy does not require substantial modification of existing interfacial polymerization equipment or production lines, the incremental

fabrication cost is expected to be minimal. Therefore, the additional economic burden associated with the −20 °C fabrication condition is unlikely to significantly affect the overall cost competitiveness of the A-NF membrane. Based on the above discussion, we have revised the corresponding section of the manuscript to incorporate the energy-consumption estimation, carbon-footprint analysis, and additional discussion on the economic feasibility of the proposed low-temperature fabrication strategy:

Line 425: However, despite its promising performance in wastewater treatment applications, further systematic studies are still needed to evaluate its scalability and behavior under full-scale operational conditions. The additional energy consumption associated with the low-temperature process was also evaluated (**Supplementary Methods**). The results indicate that these additional inputs are expected to be relatively small, suggesting that the proposed strategy remains promising from both practical and sustainability perspectives.

Reviewer #3:

Reviewer: This work presents a novel approach to membrane fabrication. Specifically, the phase inversion process is conducted a very low temperature of -20°C by doping the water phase with methanol, thereby avoiding the water freeze at normal freeze point. The resultant membrane demonstrates superior processing performance with increased flux, rejection, and fouling characteristics. These are attributed to the fact that the low temperature in the bath helps to slow down the interfacial polymerisation process and results in a thinner and smoother membrane, while maintaining NF pore size. This is a very nice piece of work and is definitely of interest to readers, I commend the authors on this front.

There are some issues with the work. Firstly, there are no stated aims at the front of the paper as would be expected. What is provided is a set of conclusive remarks based on the results which have not been presented at this point. This needs to be rationalised. Secondly, there is something very strange going on in this paper in that two experiments are reported in the text of the paper, yet the figures in the supplementary data file report on three. This has to be sorted out as confusion is introduced throughout the paper as a result. Thirdly, while I understand that authors like to sell their work, there is a lot of sensationalism in the text as provided that is not warranted. There are many areas to the text where the authors infer that the membrane is industrially significant or relevant, when the reality of the situation is that the membrane has not been tested under industrial conditions or for appropriate timescales. This language needs to be toned down, and the claims made in a more appropriate fashion. Afterall, this membrane currently only exists at the size of a postage stamp…

Finaly, this work leaves me with one major question. The authors have doped the feed solution with methanol to operate at -20°C. Why not -10°C or -30°C? If the work were to be repeated at these temperatures would a better membrane result? There has been a massive amount of time and effort placed on characterisation of the single experiment membrane when I feel a range of experimental temperatures and less characterisation may have been more beneficial. I guess this could be a subject for future work (which is not mentioned at all).

My recommendation is publication following corrections.

Our Response: We sincerely thank the reviewer for the positive and encouraging comments. We are pleased that the reviewer recognizes the novelty and potential interest of our work. We have carefully considered all the comments and have revised the manuscript accordingly. Our detailed responses are provided below.

First, we acknowledge that the original Introduction did not clearly present the aims of this work and instead included statements that resembled conclusions. To address this, we have reorganized the Introduction section, and now clearly state the research motivation, knowledge gap, and specific objectives at the end of the section.

We also apologize for the confusion caused by the inconsistency between the main text and the Supporting Information. In fact, our study was conducted at three temperature conditions (25 °C, 0 °C, and −20 °C). Due to unclear presentation in the original manuscript, this was not properly conveyed, leading to misunderstanding. We have now clarified the full experimental design in the main text, ensured consistency with the Supporting Information, and revised the relevant descriptions and figure captions accordingly.

Line 169: A series of characterization methods were used to evaluate the effect of ultra-low temperature interfacial polymerization on the structure and morphology of NF membranes. To systematically investigate the influence of interfacial polymerization temperature, three representative temperatures were selected, yielding membranes denoted as C-NF, SC-NF, and A-NF, prepared at 25 °C, 0 °C, and −20 °C, respectively.

In this context, regarding the choice of operating temperature, we would like to further clarify that −20 °C was selected because it effectively slows down the interfacial polymerization process while remaining experimentally feasible through methanol-assisted freezing point depression. The comparison across these three temperatures already provides insight into the temperature-dependent membrane formation behavior. We agree that exploring intermediate or even lower temperatures (e.g., −10 °C or below −20 °C) could offer additional understanding, and this has now been explicitly included as a future research direction in the revised manuscript.

Line 447: The use of low-freezing-point solvents to depress the aqueous-phase freezing point expands the temperature-tuning window for interfacial polymerization, opening opportunities for further optimization of membrane architecture and the development of membranes with even higher separation performance.

In addition, we appreciate the reviewer’s concern regarding overly strong or sensational wording. We agree that some statements overstated the current level of applicability. Therefore, we have carefully revised the manuscript to remove exaggerated expressions, toned down claims regarding industrial relevance, and clearly stated the current limitations, including membrane scale and testing conditions, to ensure a more balanced and rigorous presentation.

Line 295: Taken together, these results suggest that A-NF achieves a competitive balance between water permeance and ion selectivity, comparing favorably with advanced NF membranes reported in the literature. As discussed below, the exceptional water permeance and selectivity of A-NF suggest that this membrane may also perform well in salt fractionation from reverse osmosis concentrate for zero liquid discharge (ZLD) industrial wastewater treatment.

Line 425: However, despite its promising performance in wastewater treatment applications, further systematic studies are still needed to evaluate its scalability and behavior under full-scale operational conditions.

We again thank the reviewer for the constructive feedback, which has helped us significantly improve the quality of the manuscript.

1- Title: I think the word 'membranes' is missing from the end of the title, i.e. should the title be 'Suppressing Polyamide Crystallization Unlocks Ultrapermeable and Highly Selective Nanofiltration Membranes'?
Front page. Please check affiliation d, it is posted as c (c is listed twice).
Abstract: Line 38 has a random (167) in the text line. Please remove.
Line 47. Should the section title be 'Introduction' rather than 'Main'?
Line 90,93,99. Please avoid the use of personal pro-nouns, i.e. I, we…etc. Scientific text should be written in the third person. I have also noticed this issue throughout the rest of the text. Please address all instances.

Our Response:. We thank the reviewer for the careful reading of the manuscript and for these helpful suggestions. We have addressed all the points as detailed below.

Title: We appreciate the reviewer's suggestion. We have revised the title to "Suppressing Polyamide Crystallization Unlocks Ultrapermeable and Highly Selective Nanofiltration Membranes" to improve clarity and completeness.

Front page (affiliation d): Thank you for pointing this out. The affiliation labeling error has been corrected, and the duplicated "c" has been fixed in the revised manuscript.

Abstract (Line 38): We thank the reviewer for pointing this out. The "(167)" was originally intended to indicate a specific separation factor. However, we recognize that its presentation in the original version was unclear and could be mistaken as an error. We have therefore revised the expression in the Abstract.

Line 37: Comprehensive structural characterization confirms the formation of a homogeneous amorphous network, enabling a fourfold increase in water permeability (24.1 L m^{-2} h^{-1} bar^{-1}) and a threefold improvement in Cl^-/SO_4^{2-} selectivity compared with room-temperature controls, surpassing state-of-the-art NF performance..

Section title (Line 47): We appreciate the reviewer's comment. The section title "Main" is intentionally retained, as it is a format commonly used in journals within the Nature family. Therefore, we have not made changes to this part.

Use of personal pronouns (Lines 90, 93, 99, and throughout the text): We thank the reviewer for highlighting this issue. The manuscript has been carefully revised to remove the use of personal pronouns (e.g., "we", "I") and to ensure consistent use of third-person scientific writing throughout.

Line 91: In this work, high-performance NF membranes were designed by adjusting the aqueous-phase composition to lower the liquid–liquid interfacial polymerization temperature to −20 °C. A combination of polymer characterization techniques, including small-angle X-ray scattering, differential scanning calorimetry, and X-ray

diffraction, was employed to analyze the polyamide selective layer and quantify temperature-dependent changes in its crystalline structure. The water permeability, ion selectivity, and antifouling performance of the ultra-low-temperature NF membrane were evaluated through comparison with room-temperature NF membranes and representative state-of-the-art NF membranes reported in the literature. In addition, real high-salinity industrial wastewater was used to assess the membrane potential for practical wastewater treatment applications. This work provides a new perspective on NF membrane design by shifting the focus toward the intrinsic physicochemical properties of polyamide materials and highlighting the fundamental role of polymer structure regulation in the fabrication of high-performance membranes.

Line 109: During the synthesis of the NF membrane, a 30% methanol aqueous solution was used as the aqueous phase, allowing the reaction medium to remain liquid at an ultra-low temperatures of –20 °C

Line 118: As shown in **Figs. 1b and 1c**, hexane phase (right side) and water phase (left side) were constructed at different temperatures.

Line 191: In addition to conventional morphological characterization, crystallographic characterization methods were used to evaluate the effect of the ultra-low temperature polymerization system on the crystal structure of the polyamide NF membrane

Line 207: To further elucidate the effects of ultra-low-temperature liquid-liquid interfacial polymerization on the polyamide crystalline structure, C-NF, SC-NF, and A-NF were characterized using two-dimensional small-angle X-ray scattering (2D-SAXS) patterns

Line 247: The density characterization of the polyamide layer provides a new perspective on its structure. The density of the polyamide selective layer was determined using a liquid suspension method, as shown in **Supplementary Fig. 12**.

Line 399: The salt separation performance of an A-NF-based nanofiltration system was evaluated at a water recovery of 70%

Line 406: A life cycle assessment and global-warming-potential analysis were conducted to evaluate the water treatment costs and environmental impact of implementing A-NF within the salt separation and crystallization system (**Supplementary Tables 2–5**).

2- Lines 90 to 104: All of the text in this section represents conclusive remarks relating to the work presented in this paper before the results have actually been presented. Please rework this section of the text to deliver the aims of the paper and not the conclusions from the work. The aims should reflect the gap in current scientific knowledge and how the work will address those gaps.

Our Response:. We thank the reviewer for this insightful comment. We agree that the original text contained conclusive statements presented too early. In response, we have completely rewritten this section to clearly focus on the aims of the study rather than the conclusions. The revised version now explicitly identifies the existing knowledge gaps and outlines how this work is designed to address them. The specific modifications are provided below.

Line 91: In this work, high-performance NF membranes were designed by adjusting the aqueous-phase composition to lower the liquid–liquid interfacial polymerization temperature to −20 °C. A combination of polymer characterization techniques, including small-angle X-ray scattering, differential scanning calorimetry, and X-ray diffraction, was employed to analyze the polyamide selective layer and quantify temperature-dependent changes in its crystalline structure. The water permeability, ion selectivity, and antifouling performance of the ultra-low-temperature NF membrane were evaluated through comparison with room-temperature NF membranes and representative state-of-the-art NF membranes reported in the literature. In addition, real high-salinity industrial wastewater was used to assess the membrane potential for practical wastewater treatment applications. This work provides a new perspective on NF membrane design by shifting the focus toward the intrinsic physicochemical properties of polyamide materials and highlighting the fundamental role of polymer structure regulation in the fabrication of high-performance membranes.

3- Line 107: What is 'PIP' the acronym has been introduced with no explanation.

Our Response:. We thank the reviewer for pointing this out and apologize for the lack of clarity. "PIP" refers to piperazine. We have now clearly defined this acronym at its first occurrence in the revised manuscript.

Line 106: The diffusion of piperazine (PIP) molecules from the aqueous phase to the organic phase has a decisive influence on the structure of NF membranes prepared by interfacial polymerization.

4- Line 166 to 169: The surface roughness of the membranes is being discussed and figure 2c clearly indicates that the relative roughness of the resulting membranes is 21.1 nm and 2.2 nm respectively. What then is supplementary figure 3 showing with an Ra = 12.9 nm? The caption gives no information at all in relation to this phenomenon and the value is half way between the 25°C and -20°C data. This is confusing, please explain what is happening.

Our Response:. We thank the reviewer for this careful observation and apologize for the confusion caused by our unclear presentation. As noted above, our study actually includes three temperature conditions (25 °C, 0 °C, and −20 °C). The roughness value shown in Supplementary Fig. 3 (Ra = 12.9 nm) corresponds to the membrane prepared at 0 °C, which represents an intermediate state between the 25 °C and −20 °C samples, as correctly inferred by the reviewer.

We acknowledge that this was not clearly explained in the original manuscript. We have now revised the figure caption and the corresponding text to explicitly indicate this and avoid further misunderstanding.

Line 170: A series of characterization methods were used to evaluate the effect of ultra-low temperature interfacial polymerization on the structure and morphology of NF membranes. To systematically investigate the influence of interfacial polymerization temperature, three representative temperatures were selected, yielding membranes denoted as C-NF, SC-NF, and A-NF, prepared at 25 °C, 0 °C, and −20 °C, respectively.

5- Line 173 to 177. In this section of text you state that the membrane thickness is reduced from 65nm to 32nm as a result of the process. I assume that this is the fabrication of the membrane at 25°C and -20°C (note I have to assume). Then when I look at supplementary figure 5 there are three sets of plots related to this data set. These are labelled C-NF, SC-NF, and A-NF. This is very confusing as the main paper is indicating there are two sets of data, the supplementary information is telling me there are three. Similarly, the legend descriptors are not explained anywhere in the paper, I have found A-NF on line 192 but this acronym is not introduced. The other two are not explained anywhere. Lines 429-430 infer that the A-NF is the amorphous membrane and C-NF is the crystalline membrane – is this the case as I don't like to guess? I see no mention of SC-NF anywhere. Please rationalise this situation by ensuring that the methods are fully explained and the figure captions are complete, i.e. they explain the figure in full detail. Why is there a third plot when only two experiments are reported?

Our Response:. We thank the reviewer for this important comment and apologize for the confusion caused by our unclear presentation. As also noted in the previous comment, the ambiguity arises from insufficient clarification of both the experimental conditions and the membrane nomenclature in the original manuscript.

In fact, our study includes three temperature conditions (25 °C, 0 °C, and −20 °C), rather than two. The three sets of data shown in Supplementary Fig. 5 therefore correspond to these three membranes. Specifically, C-NF, SC-NF, and A-NF denote the membranes prepared at 25 °C (crystalline), 0 °C (semi-crystalline), and −20 °C (amorphous), respectively. The reviewer's inference regarding A-NF and C-NF is correct.

We acknowledge that these acronyms were not clearly defined at their first occurrence and that SC-NF was not properly introduced, which led to confusion. In addition, the main text did not clearly present the full set of experimental conditions, giving the impression that only two membranes were studied. These issues have now been corrected through revised definitions at their first occurrence and clearer presentation of the complete experimental conditions throughout the revised manuscript.

6- Line 260 introduces figure 3b. There appears to be a great many grey diamonds on this plot that make no logical sense and are not reported in the legend or caption. What are these data points? Also, at this point supplementary table 1 is introduced. Firstly, why not give the column titles proper text, i.e. specific flux (Lm-2h-1bar-1) rather than A? Secondly, this table is very dubious. What I mean by this is that rejection of salts will depend on applied pressure, concentration, and pH to name but a few factors. Therefore, to make a fair comparison, all of the data sets reported would need to be experiments conducted at the same experimental conditions. Thus, the claim that the membrane has higher selectivity is not substantiated by this table. So, I have to ask myself what is the point of this table? In addition, there are some reports in the table where the specific flux is significantly higher than that observed in this study. I would have to question if these studies are actually characterising NF membranes as fluxes this high would suggest that they are indeed UF membranes. Again, what is the purpose of this table as it does not validate the membrane characteristics obtained in this work?

Our Response:. We thank the reviewer for the careful evaluation and valuable comments. Regarding Fig. 3b, we apologize for the lack of clarity. The grey diamond symbols represent data points collected from previously published literature, which correspond directly to the entries listed in Supplementary Table 1. We acknowledge that this was not clearly explained in the original figure legend and caption. We have now revised the figure and its caption to explicitly define these symbols and improve clarity.

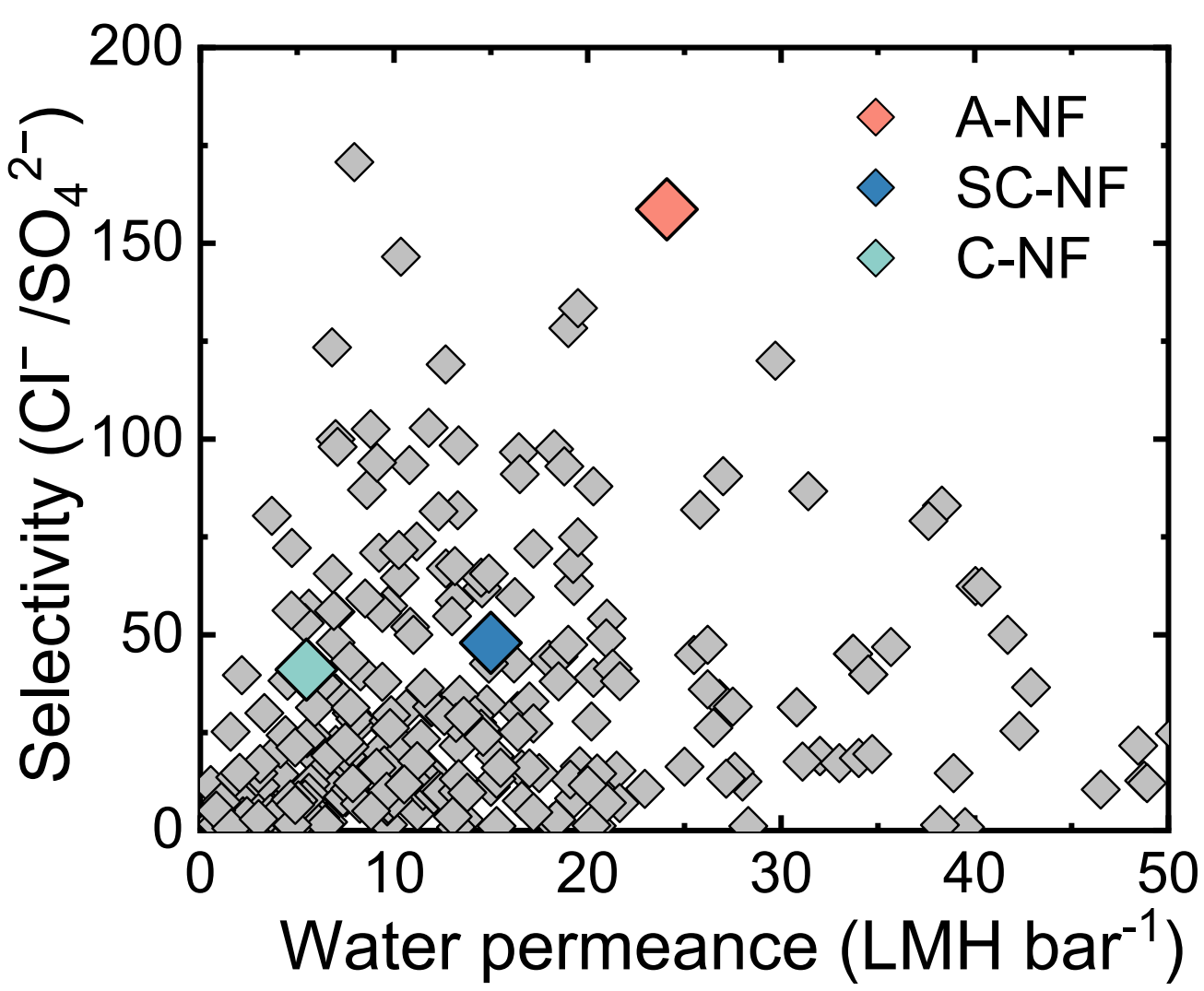

Fig. 3b: Summary of water permeance as well as NaCl/Na_2SO_4 selectivity of NF membranes reported in literature and in market. The gray diamond symbols represent literature data points corresponding to the membranes listed in Supplementary Table 1.

Concerning Supplementary Table 1, we appreciate the reviewer's comments. First, we agree that the column headings were not sufficiently descriptive. We have therefore replaced the simplified symbols with explicit terms such as specific flux (L m^{-2} h^{-1} bar^{-1}) to improve readability.

We also agree with the reviewer that membrane performance, particularly salt rejection, is strongly dependent on experimental conditions such as pressure, feed concentration, and pH. Therefore, a strictly quantitative comparison across different studies is inherently limited. The purpose of including this table was not to provide a rigorous one-to-one comparison, but rather to offer a qualitative benchmarking context to position the performance of our membrane relative to representative reports in the literature. Such comparisons under varying conditions are commonly adopted in the field to provide a general perspective [1-2].

[1] Xu, X. *et al.* Nanofiltration membranes with ultra-high negative charge density for enhanced anion sieving and removal of organic micropollutants. *Nature Water* **3**, 704-713 (2025). https://doi.org/10.1038/s44221-025-00440-9

[2] Zhao, C. *et al.* Polyamide membranes with nanoscale ordered structures for fast permeation and highly selective ion-ion separation. *Nature Communications* **14** (2023). https://doi.org/10.1038/s41467-023-36848-8

We agree that some of the reported water permeabilities listed in the table are exceptionally high and may appear comparable to those typically associated with ultrafiltration membranes. However, recent advances in nanofiltration membrane design have indeed enabled NF membranes to achieve ultrahigh water permeabilities while maintaining nanofiltration-level ion selectivity, as exemplified by Ref. 2 and Ref. 3. These membranes are therefore classified as NF membranes based on their molecular/ionic separation characteristics rather than water permeability alone.

[2] Zhao, C. *et al.* Polyamide membranes with nanoscale ordered structures for fast permeation and highly selective ion-ion separation. *Nature Communications* **14** (2023). https://doi.org/10.1038/s41467-023-36848-8

[3] Gan, B. *et al.* Fractal-inspired ultrapermeable membranes for electricity-free portable nanofiltration. *Nature Water* **4**, 68-77 (2026). https://doi.org/10.1038/s44221-025-00551-3

The primary purpose of including this comparison table is not to validate the NF characteristics of our membrane, but rather to benchmark the performance of A-NF against representative state-of-the-art NF membranes reported in the literature. Although some reported membranes exhibit higher water permeability than A-NF, their ion selectivity is often compromised. By considering both permeability and selectivity simultaneously, A-NF demonstrates a competitive overall separation performance and compares favorably with advanced NF membranes reported to date. We thank the reviewer again for these insightful comments, which have helped us significantly improve the rigor and clarity of our data presentation. We have clarified this point in the revised manuscript:

Line 292: The increased SO_4^{2-} rejection and decreased Cl^- rejection enhance the Cl^-/ SO_4^{2-} selectivity of A-NF compared to C-NF. As shown in **Fig. 3b** and **Supplementary Table 1**, the selectivity of A-NF is superior to that of state-of-the-art NF membranes reported in the literature and to that of various commercial NF benchmarks. Taken together, these results suggest that A-NF achieves a competitive balance between water permeance and ion selectivity, comparing favorably with advanced NF membranes reported in the literature.

7- Lines 266 to 272: Here you indicate that the long-term performance of NF membranes is important. I totally agree. How is 120 hours long term performance? This is 5 days of data, i.e. a working week. This is not long term at all. Please amend the text accordingly to remove the claim. Supplementary figure 14 is introduced at this point. Can you explain what the grey shadow is on the water flux data set? Is this an error bar? If it is just image and not representative of actual data then please remove as it creates confusion. In addition, most commercial NF membranes operate up to around 30-40 bar pressure, and your text indicates 'wide range of operating pressures'. This is misleading as the work is limited to the low operating pressure range as detailed in the caption on the figure. Out of interest, does this membrane offer superiority in the high-pressure ranges as well? Also of note, your figure shows a random -20°C, surely this is not the operating temperature of the experiment. Please remove and add actual conditions to the caption.
I have now seen supplementary figure 16 (SF16). Now I am confused as this figure and SF14 are surely showing the same data set with SF16 showing an increased data set. Why are both needed?

Our Response:. We thank the reviewer for the careful evaluation and constructive comments. Regarding the description of long-term performance, we agree that 120 hours cannot be considered truly long-term in the context of practical membrane operation. Our intention was to demonstrate the relative stability of the membrane over an extended laboratory testing period. We acknowledge that this wording was inappropriate. In the revised manuscript, we have modified the relevant statements to avoid overstating the significance.

Line 301: The stability of NF membranes under continuous operation is an important parameter for evaluating their practical viability. As shown in **Supplementary Fig. 18**, the Na_2SO_4 rejection of A-NF remained stable during 120 hours of continuous operation.

Regarding Supplementary Fig. 14, we apologize for the lack of clarity. The grey shadow in the water flux data represents the experimental error (standard deviation). This has now been explicitly clarified in the figure caption to avoid confusion.

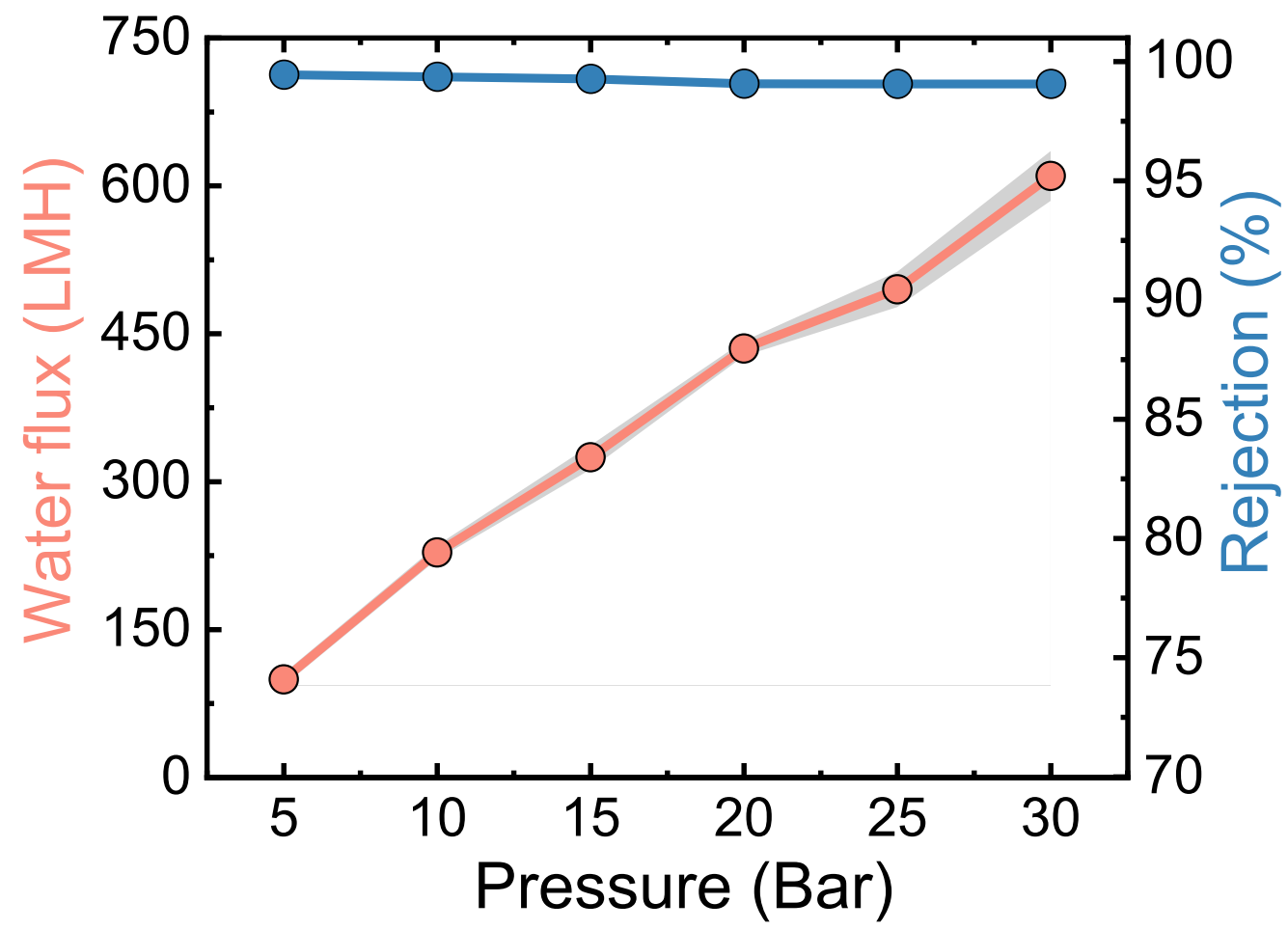

Supplementary Fig. 19: Stability of A-NF under different operating pressures. The grey shadow in the water flux data represents the experimental error (standard deviation).

Regarding the apparent overlap between Supplementary Fig. 14 and Fig. 16, we apologize for the confusion caused by the original presentation. These two figures correspond to two separate stability evaluations conducted under different operating pressures. Supplementary Fig. 14 presents stability tests under relatively low-pressure conditions, which are commonly adopted in laboratory-scale nanofiltration studies. In contrast, Supplementary Fig. 16 shows the membrane performance under higher-pressure conditions, which were included to assess its potential applicability in more practical or industrially relevant scenarios. To avoid redundancy and improve clarity, Supplementary Fig. 14 has been removed from the revised manuscript, and the corresponding text has been revised accordingly.

Line 303: In addition, the performance of A-NF was evaluated over a wide range of operating pressures (**Supplementary Fig. 19**). As the operating pressure increased from 5 to 30 bar, the water flux increased nearly linearly, while the Na_2SO_4 rejection remained at around 99%. These results demonstrate that A-NF can maintain stable separation performance under the elevated pressures commonly employed in practical nanofiltration applications.

We also thank the reviewer for pointing out the inappropriate “−20 °C” label in the figure. This value refers to the fabrication condition rather than the operating temperature. It has now been removed, and the correct experimental conditions have been clearly stated in the revised figure caption.

8- Lines 311 to 322. The inference is made here that the new membrane demonstrates better anti-fouling properties. From the data provided, I agree with these statements. However, is it fair to infer from these experiments that the membrane ‘is advantageous for applications involving industrial wastewaters’ when the conditions of these experiments are far from those that would be typical of seawater desalination or industrial

effluents? I feel the language used needs to be reflective of what has been done and not sensationalised without appropriate data to back up these statements.

Our Response:. We thank the reviewer for this thoughtful comment and for recognizing the observed anti-fouling performance. We agree that it is important to avoid overstating applicability based solely on laboratory-scale tests. We recognize that these tests are still limited in scope and do not fully represent the wide range of conditions encountered in industrial applications. Therefore, we have carefully revised the relevant statements to avoid overgeneralization.

Line 342: NF membrane fouling leads to a sharp decline in water flux and necessitates higher operating pressures, which drives up energy consumption and costs while compromising both separation efficiency and system longevity. The smooth surface of A-NF results in lower adhesion of foulants. Furthermore, the high permeability of A-NF to Ca^{2+} alleviates inorganic scaling as well as organic fouling.

9- Again, as with lines 329 to 336, 1500 minutes is one day of operation. Significant fouling occurs over time, and this experiment is not based over a long time period. So, while I agree that the demonstrated effect of less fouling is there, the text needs to be contextualised in light of the demonstrated operation and not inferred that there is superior industrial performance when this has not been demonstrated.

Our Response:. We thank the reviewer for this important comment and for recognizing the observed anti-fouling behavior. We agree that a testing duration of 1500 minutes (~1 day) cannot be considered representative of long-term operation in practical applications. In response, we have revised the relevant text to better contextualize these results, avoiding any implication of demonstrated industrial superiority, and the language has been adjusted to clearly reflect the limited duration of the experiments.

Line 342: NF membrane fouling leads to a sharp decline in water flux and necessitates higher operating pressures, which drives up energy consumption and costs while compromising both separation efficiency and system longevity. The smooth surface of A-NF results in lower adhesion of foulants. Furthermore, the high permeability of A-NF to Ca^{2+} alleviates inorganic scaling as well as organic fouling.

10- Methods: Where was the ultrapure water obtained?

Line 459: Why is the symbol ‘S’ used for membrane area rather than ‘A’ as would be expected? Why then is specific flux termed ‘A’ when papers would normally use ‘J’ for flux and ‘P’ for permeance? Why add confusion by swapping from normal nomenclature.

Line 463: Again, why is symbol ‘Co’ used in place of the typically expected ‘Cp’ the concentration in the permeate?

Line 542: Reference 20 has an anomalous '1', please remove.

Our Response:. We thank the reviewer for the careful reading and helpful comments. The ultrapure water used in this study was produced using a laboratory ultrapure water system, and the specific model has now been added to the revised manuscript.

Regarding the notation used in the Methods section, we acknowledge the reviewer's concern. The symbols adopted in this work are commonly used in membrane literature. We have carefully revised the manuscript to standardize the symbols and align them more closely with widely accepted conventions where appropriate, and ensured that all variables are clearly defined at their first occurrence.

Finally, we thank the reviewer for pointing out the error in Reference 20. The anomalous "1" has been removed in the revised manuscript.

Line 471: Unless otherwise stated, all solutions used in this work were prepared with 18-MΩ ultrapure deionized water (PURELAB flex Ultrapure Water System).

Line 511: The water permeance (*P*) of the membrane (L m^{-2} h^{-1} bar^{-1}) was calculated by

$$P = \Delta V/(A\Delta t\Delta P) \quad (1)$$

where ΔV is the volume of permeate water (L) collected during a time interval of Δt (h), *A* is the effective filtration area (m^2), and ΔP is the applied pressure (bar), respectively. The rejection efficiency (*R*) was calculated according to

$$R = (1 - C_p/C_0) \times 100\% \quad (2)$$

where C_0 and C_p are the salt concentrations in the feed liquid and the filtrate, respectively.

Reviewer #1:

Questions addressed. Can be accepted now.

Our Response: Thank you very much for your time and careful evaluation. We sincerely appreciate your positive assessment and support.

Reviewer #2:

better

Our Response: We sincerely thank the reviewer for their positive evaluation of our revised manuscript. We appreciate the constructive feedback provided during the review process, which significantly contributed to improving the clarity and quality of our work.

Reviewer #3:

Dear Editor and authors
I have now taken the time to read through the responses to the original review questions that I made and am happy that the reviewers have taken on board all of the points raised. I am happy for the paper to progress to publication. I would like to thank the authors for the rigorous responses which made review far easier.

Our Response: We sincerely thank the reviewer for their positive comments and thoughtful evaluation. We are grateful for your support and are pleased that the revised version is now considered suitable for publication.
